# The Role of Nurses in Rehabilitation in Primary Health Care for Ageing Populations: A Secondary Analysis from a Scoping Review

**DOI:** 10.1177/23779608241271677

**Published:** 2024-09-23

**Authors:** Viola Lorenz, Vanessa Seijas, Heidrun Gattinger, Claudia Gabriel, Margrieta Langins, Satish Mishra, Carla Sabariego

**Affiliations:** 1 Faculty of Health Sciences and Medicine, 30731 University of Lucerne, Lucerne, Switzerland; 2 Center for Rehabilitation in Global Health Systems, WHO Collaborating Center, 30731 University of Lucerne, Lucerne, Switzerland; 3 590708 Swiss Paraplegic Research, Nottwil, Switzerland; 4 IPW Institut für Angewandte Pflegewissenschaft, 30826 OST Eastern Switzerland University of Applied Sciences, St. Gallen, Switzerland; 5 Clinic for Neurology and Neurorehabilitation, Care Development and Quality of Care, 30748 Cantonal Hospital Lucerne, Lucerne, Switzerland; 6 Nursing and Midwifery Policy Adviser, Health Workforce and Service Delivery Unit, Division of Country Health Policies and Systems, 93773 WHO Regional Office for Europe, Copenhagen, Denmark; 7 Disability, Rehabilitation, Palliative and long-term Care, Health Workforce and Service Delivery Unit, Division of Country Health Policies and Systems, 93773 WHO Regional Office for Europe, Copenhagen, Denmark

**Keywords:** ageing population, healthy ageing, nursing, primary health care, rehabilitation

## Abstract

**Introduction:**

With the ageing of the global population and the rise in noncommunicable diseases, there is an increased need for rehabilitation services, especially those that address the specific needs of ageing populations. Through their proximity to patients, nurses play a critical role in providing rehabilitation interventions for older adults in primary health care. However, they are not yet established as typical rehabilitation providers and further research is needed to clarify their role and competencies to optimize rehabilitation interventions for ageing populations.

**Objectives:**

Therefore, this secondary analysis of a scoping review aims to describe the role of nurses in the provision of rehabilitation interventions to ageing populations in primary health care.

**Methods:**

This review was carried out following the Preferred Reporting Items for Systematic reviews and Meta-Analyses extension for Scoping Reviews. It is built on all inclusion and exclusion criteria from the primary analysis and focused on studies including nurses and taking place in primary health care. Data analysis included descriptive statistics, as well as qualitative analysis on the role of nurses.

**Results:**

A total of 68 studies from high- and upper-middle income countries were included. Nurses typically had a managerial and clinical role (76%) and worked in multidisciplinary teams (54%), most often with physical therapists. Nurses provided 355 interventions, with assessments (n = 106; 30%), and coordination and management of the rehabilitation process (n = 105; 30%) being the most frequent ones. They had 117 different job titles and little information was available about their educational background.

**Discussion:**

This paper contributes to a better understanding of the key role nurses play in providing rehabilitation interventions to ageing populations in primary health care. Matching nurses’ competencies with their level of proficiency is essential to ensure quality care in rehabilitation.

## Introduction

The world is currently facing two rapid trends. First, the population is ageing worldwide: in 2021, the proportion of people aged 65 years and over was 10% and is expected to rise to 16% by 2050 (United Nations Department of Economic and Social Affairs Population Division, 2022). This has been observed for several years in high-income countries (HICs), which is why they have the highest proportion of older people. However, it is of particular concern that the increase is also occurring in low- and middle-income countries (LMICs), even at a faster pace (United Nations Department of Economic and Social Affairs Population Division, 2022). At the same time, the incidence and prevalence of noncommunicable diseases (NCDs) are also rising. NCDs—namely cardiovascular diseases, cancer, chronic respiratory diseases, and diabetes—are currently the largest contributors to mortality and disability worldwide (World Health Organization, 2022a). According to the World Bank (2020), they account for 74% of all deaths worldwide, with 88% in HICs and 70% in LMICs in 2020 (World Bank, 2020). LMICs also continue to have a high prevalence of communicable diseases, resulting in a double burden of diseases (World Health Organization, 2022a). These two trends lead to people living longer and suffering more from complex and chronic diseases, requiring health systems to adapt their services to meet the changing needs of ageing populations.

According to the World Health Organization (WHO), at least 142 million older people worldwide are unable to carry out basic activities, such as dressing themselves or taking their medications, and would benefit from rehabilitation services (World Health Organization, 2020a). To achieve this, the WHO and the United Nations (UN) launched the “UN Decade for Healthy Ageing 2021–2030” (mentioned as “the decade” in this paper) and urged for a global call for action. In their report, healthy ageing was defined for the first time as “*the process of developing and maintaining the functional ability (FA) that enables well-being in old age*” (World Health Organization, 2020a). The FA, or functioning, is the primary outcome of rehabilitation and results from the interaction of a person's intrinsic capacity (IC)—all mental and physical abilities—with their environment (World Health Organization, 2015). As people age, the IC increasingly declines, bringing with it complex diseases that are difficult to manage and costly to health systems (Fong, 2019; World Health Organization, 2015). Rehabilitation is further defined as “*a set of interventions designed to optimize functioning and reduce disability in individuals with health conditions in interaction with their environment*” (World Health Organization, 2017). Therefore, rehabilitation is considered as a key strategy for achieving healthy ageing in the decade and should be systematically integrated into all levels of care (World Health Organization, 2020a).

Primary health care (PHC) is the heart of health systems and represents the level of care at which health services are coordinated with one another. It is considered the gateway to health care because people can access specialized health services through it (World Health Organization, 2018b). Through its central role, a strong PHC is “*the most inclusive and cost-effective way*” to achieve universal health coverage (UHC) and sustainability development goal 3 (SDG3): “*to ensure healthy lives and promote well-being for all at all ages*” (World Health Organization & United Nations Children’s Fund (UNICEF), 2019). PHC includes various health services from “*health promotion to disease prevention, treatment, rehabilitation, palliative care, and more*” (World Health Organization & United Nations Children’s Fund (UNICEF), 2018). Although rehabilitation is an essential health service at this level, it is still insufficiently integrated into PHC because some misconceptions persist, such as considering rehabilitation as “*a fallback strategy when preventive or curative interventions fail*” or “*a luxury or optional health service for those who can afford it*” or that it is “*only disability-specific*” (World Health Organization, 2018a). In May 2023 the first-ever World Health Assembly resolution on rehabilitation was unanimously adopted. The resolution urges all member states to expand rehabilitation to all levels of care, from primary to tertiary to ensure the availability and affordability of quality and timely rehabilitation services (World Health Assembly, 2023).

To achieve desired outcomes, rehabilitation services must be provided by multidisciplinary teams in which nurses play an essential role (European Observatory on Health Systems and Policies et al., 2019; World Health Organization, 2018a). Nurses are the largest professional group in health systems, accounting for 61% of all health workers in the WHO European Region (World Health Organization, 2022c). Their initial clinical assessment forms the basis for further treatment and is critical to health care delivery, as they are often the only health professional a patient sees (World Health Organization, 2020b). Regarding the principle of teamwork in rehabilitation, it is of great importance to describe the competencies and roles of health workers with different educational backgrounds. A clear role definition can potentially avoid overlap and insufficient or inappropriate use of the competencies between health workers and also within the same profession (Gutenbrunner et al., 2022). Rehabilitation nursing was organized as a specialty practice in 1964, with the goal to “*manage and promote the care of persons with disability and chronic health conditions across the continuum of care through special knowledge and expertise*” (Vaughn et al., 2022). In 1984, the Certified Rehabilitation Registered Nurse designation was recognized by the American Nurses Association. In 2014, the “ARN Competency Model of Professional Rehabilitation Nursing” was published, providing “*a lens through which professional nurses can view their practice in multiple settings across the healthcare continuum*” (Association of Rehabilitation Nurses, 2014, S. 11). Despite of these efforts, there is still a gap between the relevance and evaluation of the role of nursing in rehabilitation (Gutenbrunner et al., 2021; Nolan & Nolan, 1999). The role of nurses in rehabilitation is not always recognized and is therefore not supported (Nelson & Gordon, 2004). Given the two global trends mentioned above and the added value of rehabilitation nursing, it is critical to better understand the nurses’ role in rehabilitation of ageing populations in PHC to achieve UHC and the SDG3 (World Health Organization & United Nations Children’s Fund (UNICEF), 2018).

We therefore conducted a secondary analysis of a recent scoping review (Seijas et al., 2024) with the objective of describing the role of nurses in the provision of rehabilitation interventions to ageing populations in PHC.

## Methods

This secondary analysis of a scoping review (Seijas et al., 2024) will explore the following research question: Which role do nurses have in the provision of rehabilitation interventions to ageing populations in PHC? To focus the research question, the population, concept, and context framework recommended for scoping reviews by the Joana Bridge Institute (JBI) (Aromataris & Munn, 2020) was elaborated as follows: population: ageing populations, concept: nurses’ role in providing rehabilitation interventions, context: PHC and community-based care.

### Study Design

This study is a secondary analysis of a scoping review titled “*Rehabilitation delivery models to foster healthy ageing—a scoping review*” (Seijas et al., 2024) which aimed to provide a systematic overview of rehabilitation delivery models used to optimize the IC and functioning/functional ability of older adults (Seijas et al., 2024); the protocol and addendum of the primary review is available in Appendix A in the online supplementary file 1. This secondary analysis was conducted in accordance with the JBI methodology for scoping reviews (Aromataris & Munn, 2020) and the Preferred Reporting Items for Systematic reviews and Meta-Analyses extension for Scoping Reviews (PRISMA-ScR) (Tricco et al., 2018). The checklist is available in Appendix A in the online supplementary file 2. To increase research transparency and avoid duplicates, the study protocol has been registered in the Open Science Forum and the preprint is available on Research Square. The secondary analysis protocol is available in Appendix A in the online supplementary file 3. As no human subjects are involved, no ethical approval was required. The study design has been chosen based on Kazi et al. (2021) recommendations, which states that scoping reviews are a useful method for mapping data by selected key topics, enabling broad capture of information and identifying research gaps in the literature.

### Eligibility Criteria

All inclusion and exclusion criteria were built based on the original criteria and adapted to answer the secondary analysis objectives and are described below.

#### Population

Study population with a “mean age of 50 years or over” was included. The evidence that countries with similar levels of age-related burden experience different onsets of ageing was considered. When the mean age was not available, studies that target multiple age-related diseases, whose incidence rates increase quadratically with age were included (Chang et al., 2019). Diseases from selected clusters defined by Kuan et al. (2021) were used (Seijas et al., 2024).

#### Concept/Intervention

Studies describing or testing models of care, service delivery methods, modes of service delivery, care services, care programs, and organization of care were included. Studies should have aimed to enable healthy ageing through improving functioning and IC and reducing the experience of disability of older people living with a health condition. Nurses should have been part of the rehabilitation team. We excluded studies that focus on describing needs, functional patterns, disability, risk factors, or protective factors of the ageing population. Also studies that have only other types of outcomes such as morbidity, mortality, disease control-related outcomes, interventions adherence, interventions’ perceived quality, and willingness to continue, enjoyment, participation, health services use, caregivers’ burden, implementation barriers, or health workers perceptions were excluded (Seijas et al., 2024).

#### Context/Setting

We only included studies in which interventions occurred at the PHC level, or a combination of PHC and specialized care levels. Studies that were strictly limited to interventions taking place at specialized care levels only were excluded.

#### Publication Type

Primary studies with a quantitative, qualitative, or mixed-methods design were included. Books, book chapters, narrative reviews, systematic reviews, meta-analyses, position papers, guidelines or recommendations, letters to the editor, conference proceedings, or retraction letters were excluded (Seijas et al., 2024).

#### Language and Publication Year

We included studies published in English from January 2015 to the end of May 2022.

### Information Sources and Search Strategy

No new search strategy has been developed for this secondary analysis and no new literature search has been conducted. Search concepts, terms, and the full electronic search strategies for each bibliographic database queried are available in Appendix A in the online supplementary file 4.

### Study Selection Process

For the primary review, three researchers independently screened and reviewed the titles and abstracts, with half being double-checked. Training rounds were conducted to ensure consistent decision-making, with 90% agreement achieved after three sessions. The full text of selected records was then reviewed for eligibility, with any disagreements resolved by discussion among the authors. In this secondary analysis, two additional criteria were applied to identify relevant studies using two variables obtained during the data extraction process of the primary review: first, the variable “*level of care*” was filtered by “*multiple levels of care*” and “*primary health care*.” Then, the variable “*health workers*” was filtered by “*nurses*.” After this process, VL double-checked all titles and abstracts of the selected studies to ensure that they met the criteria and that no errors were made in the data extraction of the primary review.

### Data Extraction Process

All variables extracted in the primary review were included. However, the variable “*Role or task shifting*” was completely revised with a focus on nurses. In addition, 38 new variables were added to the data extraction form based on the “*WHO Operational Framework for Primary Health Care Transforming Vision into Action*” (World Health Organization & United Nations Children’s Fund (UNICEF), 2020). All variables for this review are presented in more detail in Appendix A in the online supplementary file 5. The quality of the selected studies was not critically assessed. The authors considered that, as the aim of this review was not to analyze the effectiveness of the interventions or the appropriateness of the role of nurses in the provision of rehabilitation, the assessment of the risk of bias was not critical to the interpretation of the results.

### Data Synthesis

Data synthesis included quantitative and qualitative analysis. For the quantitative part, descriptive statistics were calculated using Microsoft Excel (Version 2021 for Windows) for the characteristics of the studies’ target population, the characteristics of the rehabilitation interventions, and the characteristics of the nurses’ role. Consistent with the primary review, categorization of the interventions was based on the “*International Classification of Health Interventions*” (ICHI) (World Health Organization, 2022b) and the “*WHO Packages of Interventions for Rehabilitation*” included in the “*Universal Health Coverage Compendium*” (UHC) (World Health Organization, n.d.). The interventions corresponded to the “*action level*” in the ICHI and UHC taxonomies (Seijas et al., 2024). To define the levels of care and mode of service delivery, the “*International Classification of Service Organization in Rehabilitation*” (ICSO-R 2.0) (Gutenbrunner et al., 2020), the “*Effective Practice and Organisation of Care Taxonomy of Health Systems Interventions*” (The Cochrane Effective Practice and Organisation of Care (EPOC) group, 2015), and the WHO definition of models of care (Tello et al., 2019) were adopted (Seijas et al., 2024). Papers were categorized as PHC when articles self-identified as PHC, when rehabilitation interventions were provided solely by PHC workers—including nurses or general practitioners—in a traditional PHC setting (home or community) or when interventions provided did not require complex equipment or specialized training.

For this review, data were categorized according to whether study participants could decide which rehabilitation program they received and if they were involved in its design. Furthermore, single interventions of rehabilitation programs were extracted, as well as the location where the interventions took place (e.g., rural, urban setting). The role of nurses was analyzed descriptively in terms of the content of the interventions. First, text passages describing the role of nurses were copied and pasted into the data extraction form. Then, matching was performed with the categorized rehabilitation interventions from the primary review, and only those that were either directly delivered by nurses or involved nurses were considered and recorded in the new variable. Interventions were further classified as “*managerial*” if nurses had administrative tasks and/or held a managerial position. If nurses were involved in clinical practice, for example, setting person-centered goals with the patients, they were classified as “*clinical*.” If nurses performed a combination of both, they were classified as “*managerial and clinical*.” Then, it was assessed if and what kind of task-shifting took place toward nurses. For this, the scope of practice of rehabilitation nurses was based on the “*ARN's Core Curriculum*” (Edwards, 2000) and task-shifting was categorized as enhancement, innovation, and substitution in accordance with the European Commission (European Commission Directorate-General for Health and Food Safety, 2019). For the nurses’ communication, any available information was extracted openly. The nurses’ training and work independence were also extracted openly. The nurses’ job titles were copied and pasted into the data extraction form and categorized using an inductive and descriptive approach. Finally, nurses’ competencies were determined based on the interventions they provided in the four domains of the updated Association of Rehabilitation Nurses (ARN) Competency Model of Professional Rehabilitation Nursing (mentioned as the “updated ARN Model” in this paper) (Vaughn et al., 2022).

## Results

### Study Selection and Characteristics

**Figure 1.**
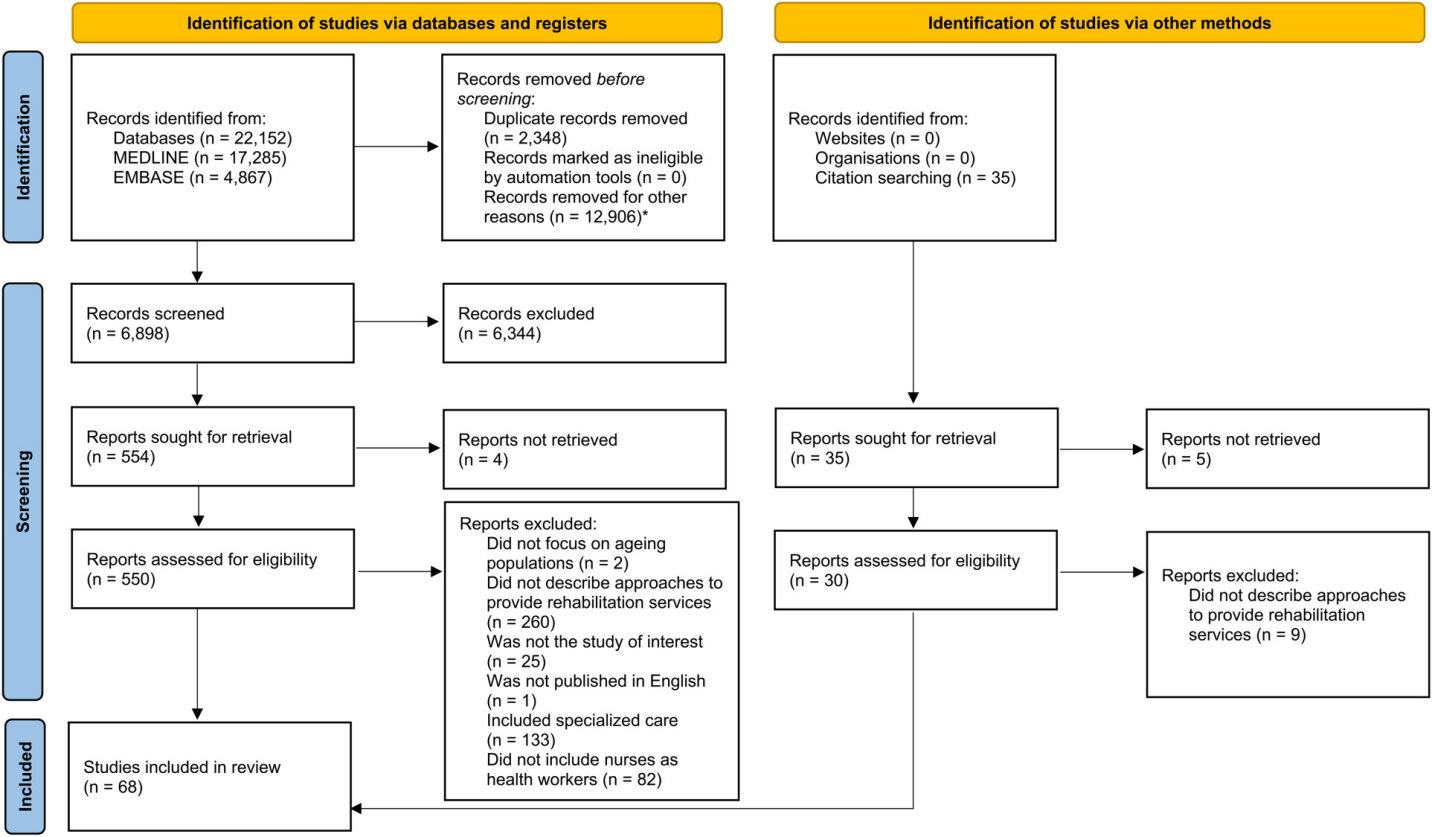
PRISMA flow diagram.

The primary search identified 22,152 studies, of which 68 studies, with a total of 35,572 participants, were included after applying the eligibility criteria for this secondary analysis. The PRISMA flow diagram was generated online according to Haddaway et al. (2022) and is provided in Figure 1. An overview of all the included studies is available in Appendix B in the online supplementary file 1. Most of the studies were published in 2016 (*N* = 19; 28%) and were randomized controlled trials (RCTs) (*N* = 38; 56%). All studies were conducted in HICs (*N* = 57; 84%) and upper-middle income countries (*N* = 11; 16%). Most of the studies included integrated care (*N* = 40; 59%). The main characteristics of the included studies are presented in Table 1.

**Table 1.** Main Characteristics of the Included Studies.

Category	Details	*N*	%
Year of publication	2016	19	28
	2018	15	22
	2015	12	18
	2021	8	12
	2017	4	6
	2020	4	6
	2019	3	4
	2022	3	4
Study design	Randomized controlled trials^a^	38	56
	Quasiexperimental design	8	12
	Cohort study^b^	8	12
	Model development	4	6
	Mixed methods	2	3
	Pretest posttest	2	3
	Qualitative study	3	4
	Narrative model description	1	1
	Intervention study (not specified)	1	1
	Case-control study	1	1
Countries	Netherlands	17	25
	China^c^	10	15
	Australia	6	9
	United States	5	7
	Spain	4	6
	Sweden	4	6
	China (Hong Kong)	3	4
	Denmark	3	4
	South Korea	3	4
	United Kingdom^d^	3	4
	Japan	2	3
	Korea	2	3
	Mixed countries^e^	2	3
	Italy	1	1
	Turkey	1	1
	New Zealand	1	1
	Canada	1	1

*Note.* The percentages were calculated from *N* = 68 (all included studies).

^a^ Including one pilot randomized controlled trial, and one cluster-controlled trial.

^b^ Including one prospective cohort study.

^c^ Including one study from Taiwan.

^d^ Including one study from Scotland.

^e^ Including one study from Switzerland/Netherlands and one study from United Kingdom, Greece, Croatia, Netherlands, and Spain.

### Characteristics of the Studies’ Target Population

The population median of all the included studies was 214 participants (min: 1; max: 8,217). The mean age of the included participants was 74 years (median: 76.7; min: 51.9; max: 87). Participants received rehabilitation interventions mostly due to a general decline in functioning (*N* = 35; 51%). Neurological (*N* = 9; 13%) and cardiovascular diseases (*N* = 8; 12%) were the most frequent health condition areas. Stroke (*N* = 7; 10%), chronic obstructive pulmonary disease (*N* = 6; 9%), and hip fracture or post hip arthroplasty (*N* = 5; 7%) were the most common health conditions. The main characteristics of the studies’ target population are presented in Table 2.

**Table 2.** Main Characteristics of the Studies’ Target Population.

Category	Details	*N*	%
Age-related inclusion	Not specified	21	31
	Older than 65	17	25
	Not applicable	8	12
	Older than 60	6	9
	Older than 18	6	9
	Older than 75	5	7
	Older than 70	3	4
	Older than 80	1	1
	Ceiling age at 75	1	1
	Ceiling age at 70	1	1
Participants’ sex predominance	Similar	28	41
	Female predominance	26	38
	Male predominance	10	15
	Not specified	4	6
Target population	People with a decline in functioning	35	51
	People with a single health condition	29	43
	People with more than two health conditions	4	6
Health condition area	Neurological	9	13
	Cardiovascular	8	12
	Musculoskeletal	7	10
	Respiratory	6	9
	Not specified	2	3
	Metabolic	1	1
Health conditions	Stroke	7	10
	Chronic obstructive pulmonary disease	6	9
	Hip fracture or posthip arthroplasty	5	7
	Coronary heart disease	3	4
	Heart failure	3	4
	Not specified	3	4
	Cardiovascular disease not specified	2	3
	Cognitive impairment, incl. dementia/aphasia	2	3
	Inflammatory arthritis	1	1
	Diabetes	1	1
	Osteoarthritis	1	1

*Note*. The percentages were calculated from *N* = 68 (all included studies).

### Characteristics of Rehabilitation Interventions

Most studies had only one mode of service delivery (*N* = 37; 54%), with the outpatient mode being the most common one (*N* = 31; 46%). There was not much data available on the geographical location of the intervention. However, 10 studies (15%) took place in rural (Barker et al., 2016; Blom et al., 2016; Deng et al., 2020; Duncan et al., 2018; He et al., 2018; Lou et al., 2014; Oh et al., 2021; Tarazona-Santabalbina et al., 2016; Taube et al., 2018; Van Lieshout et al., 2018) and 10 studies (15%) in urban settings (Everink et al., 2017, 2018; Franse et al., 2018; Inzitari et al., 2018; Kono et al., 2016; Mas et al., 2016; Noh et al., 2021; Valdivieso et al., 2018; L. Zhang et al., 2017; P. Zhang et al., 2018). Only seven studies (10%) reported explicitly to have involved the ageing population in the design of the rehabilitation intervention (Everink et al., 2018; Inzitari et al., 2018; Kidd et al., 2015; King et al., 2018; Looman et al., 2016; Shinkai et al., 2016; Van Der Weegen et al., 2015). Half of the rehabilitation programs were carried out by multidisciplinary teams (*N* = 37; 54%). Health workers were included in the provision of all interventions (*N* = 68; 100%). In addition, eight studies were provided by informal caregivers and family members (12%), and four further studies by peers and volunteers (6%). Nurses collaborated most often with physical therapists (*N* = 26; 38%). Most interventions featured multiple sessions (*N* = 53; 78%), and the components were most frequently prespecified and adapted considering the patient's needs (*N* = 27; 40%). The main characteristics of the rehabilitation interventions are provided in Table 3.

**Table 3.** Main Characteristics of the Rehabilitation Interventions.

Category	Details	*N*	%
Mode of service delivery	Outpatient	31	46
	Inpatient	22	32
	Home	21	31
	Community	16	24
	Telerehabilitation	10	15
	Eldercare	4	6
Rural or urban	Not specified	47	69
	Urban	10	15
	Rural	10	15
	Rural and urban	1	1
Service provider	Health workers	68	100
	Informal caregivers and family	8	12
	Peers and volunteers	4	6
Health workers	Nurses	68	100
	Physical therapists	26	38
	General practitioners	24	35
	Physicians	17	25
	Occupational therapists	15	22
	Social workers	14	21
	Other physicians	13	19
	Dieticians	13	19
	Geriatricians	8	12
	Physical rehabilitation medicine physicians	5	7
	Psychologists	5	7
	Speech and language therapists	4	6
	Community health workers	1	1
	Optometrists	1	1
	Neurologists	1	1
	Exercise professionals	1	1
Provision of the interventions’ components	Adapted components considering patients’ needs and prespecified components	27	40
	Adapted components considering patients’ needs	22	32
	Prespecified components	19	28

*Note*. The percentages were calculated from *N* = 68 (all included studies).

### Characteristics of the Nurses’ Role

Nurses typically had a clinical and managerial role (*N* = 52; 76%). The training they received was only described in 28 studies (41%). They were trained, for example, on assessing patients’ needs (Markle-Reid et al., 2018), using care plans with an integrative approach (Blom et al., 2016) or planning discharge (P. Zhang et al., 2018). A table summarizing all information about the nurses’ training is available in Appendix B in the online supplementary file 2. Unfortunately, the studies did not provide any information on how nurses collaborated with other rehabilitation providers. Therefore, it was not possible to extract data on the nurses’ communication and determine their degree of work independence. However, 26 studies (38%) mentioned that interventions were nurse-led (Bleijenberg et al., 2016; Buurman et al., 2016; Cameron-Tucker et al., 2016; Clevenger et al., 2018; Coskun & Duygulu, 2022; Everink et al., 2017; Finlayson et al., 2018; Franse et al., 2018; Hoedemakers et al., 2022; Wong et al., 2022; etc.). Out of these, ten interventions focused on transitional care, where nurses helped to coordinated and provided interventions to the ageing population across different levels of care (Buurman et al., 2016; Coskun & Duygulu, 2022; Everink et al., 2017, 2018; Finlayson et al., 2018; Ko et al., 2019; Lindhardt et al., 2019; Smith & Fields, 2020; Wong et al., 2022; P. Zhang et al., 2018). Task-shifting occurred in 17 studies (25%) and was not always carried out the same way. It mostly enfold as enhancement (*N* = 7; 41%), when nurses, for example, led the coordination of the rehabilitation process (Coskun & Duygulu, 2022), or the comprehensive geriatric assessment (Buurman et al., 2016; King et al., 2018). It could be interpreted as substitution (*N* = 6; 35%), when nurses provided education and counseling for physical activity and therapeutic exercise (Ko et al., 2019; Tseng et al., 2021). Finally, in three studies (18%) task-shifting occurred as innovation when nurses used new digital technologies (van Dijk-de Vries et al., 2015). The main characteristics of the nurses’ role are presented in Table 4. More information on rehabilitation interventions provided by nurses, as well as on nurses’ job titles and nurses’ competencies can be found in the following subsections.

**Table 4.** Main Characteristics of the Nurses’ Role.

Category	Details	*N*	%
Nurses’ role (*N* = 68)	Managerial and clinical	52*	76*
	Clinical	12*	18*
	Not specified	4*	6*
Nurse-led interventions (*N* = 68)	No	41*	60*
	Yes	26*	38*
	Not applicable	1*	1*
Task shifting toward nurses (*N* = 68)	No	51*	75*
	Yes	17*	25*
Rehabilitation intervention category (*N* = 355)	Assessments	106**	30**
	Coordination and management of the rehabilitation process	105**	30**
	Restorative and compensatory interventions	78**	20**
	Education and counseling	51**	14**
	Social care and support	19**	5**
	Training in the use of assistive products	2**	1**
Nurses’ job title category (*N* = 117)	Information about nurses’ function	24***	21***
	Information about nurses’ working place	23***	20***
	Not applicable^a^	22***	19***
	Information about nurses’ education	19***	16***
	Information about nurses’ medical expertise	16***	14***
	Multiple information^b^	13***	11***

^a^ This applied when studies only used the job title “Nurse”.

^b^ Multiple information types were combining working place and education (e.g., Community care registered nurse), the medical expertise and education (e.g., Geriatric nurse practitioner), the function and working place (e.g., Head nurse in the geriatric ward), the function and education (e.g., Registered practice nurse), and the medical expertise and education (e.g., Respiratory nurse specialist).

*The percentages were calculated from *N* = 68 (all included studies).

**The percentages were calculated from *N* = 355 (total number of interventions).

***The percentages were calculated from *N* = 117 (total of job titles).

### Rehabilitation Intervention Provided by Nurses

In total, nurses provided 355 interventions with assessments (*N* = 106; 30%) and coordination and management of the rehabilitation process (*N* = 105; 30%) being the most frequent ones. In terms of assessments, nurses evaluated most frequently person-centered goals (*N* = 32; 9%) and functioning (*N* = 21; 6%). They further coordinated and managed the rehabilitation process most frequently through follow-up visits (*N* = 25; 7%) and case management (*N* = 23; 6%). Nurses further provided education, counseling, and skills training, most often for self-care and self-management (*N* = 26; 7%). As restorative and compensatory interventions, nurses provided multicomponent care, which was often not further specified (*N* = 25; 7%) and therapeutic exercises (*N* = 18; 5%). Among those were, for example, deep breathing exercise, leg abduction, changing position, sitting exercise, taking wheelchair, and walking with a walker (Ko et al., 2019). In one study they aimed to facilitate postoperative mobility (Tseng et al., 2016). Nurses provided the exercise in hospital and or at home, thus assuring the continuity in care transition (Tseng et al., 2021). Two studies (1%) reported that nurses provided psychological interventions, such as individualized consultation for patients at-risk for depression (Tseng et al., 2015), or behavioral activation and antidepressant treatment sessions, which were supervised by a psychiatrist (Bekelman et al., 2015). Nurses also provided social care, as emotional (*N* = 4; 1%) and peer support (*N* = 3; 1%). Finally, nurses provided training in the use of assistive products in two studies (1%). Table 5 gives an overview of all rehabilitation interventions provided by nurses.

**Table 5.** Overview of All Rehabilitation Intervention Types Provided by Nurses.

Categories	Intervention types	*N*	%	*N* total	% total
Assessments	Person-centered goals	32	9	106	30
	Functioning (overall)	21	6		
	Comprehensive geriatric assessment	12	3		
	Emotional functions	8	2		
	Fall risk	6	2		
	Frailty	6	2		
	Environment	5	1		
	Health status	5	1		
	Medication	5	1		
	Cognitive functions	4	1		
	Family and caregivers’ needs, knowledge and skills	2	1		
Coordination and management of the rehabilitation process	Follow-up visits	25	7	105	30
	Case management	23	6		
	Discharge planning	15	4		
	Not further specified	14	4		
	Health status monitoring	12	3		
	Monitoring of functional ability	12	3		
	Home visits	4	1		
Restorative and compensatory interventions	Multicomponent rehabilitation care or not specified	25	7	72	20
	Therapeutic exercises	18	5		
	Motivational interventions	7	2		
	Cognitive training	4	1		
	Management of incontinence	3	1		
	Behavioral interventions	3	1		
	Problem solving skills training	3	1		
	Management in the use of medication	2	1		
	Psychological interventions	2	1		
	Social skills training	2	1		
	ADL training	2	1		
	Therapeutic recreation	1	0		
Education and counseling	Education and skills training for self-care and self-management	26	7	51	14
	Education and skills training for caregivers	13	4		
	Education and counseling about healthy lifestyle behaviors	6	2		
	Education and counseling for physical activity and therapeutic exercise	3	1		
	Education and skills training for activities of daily living	2	1		
	Education and counseling about healthy diet and nutritional requirements	1	0		
Social care and support	Social care and support plan	12	3	19	5
	Emotional support	4	1		
	Peer support	3	1		
Training of assistive products	Provision and training in the use of assistive products	2	1	2	1

*Note.* The percentages were calculated from *N* = 355 (total numbers of interventions).

ADL = Activities of Daily Living.

### Nurses’ Job Titles

The studies described 117 different nurses’ job titles and the most frequent one was simply “Nurse” (*N* = 22; 19%). Despite the diversity of job titles, it was possible to group them into four broad descriptive categories: 21% of the job titles (*N* = 24) provided information on the nurses’ function, for example, “Practice nurse” (*N* = 7; 6%). Then, 20% of the job titles (*N* = 23) further referred to the nurses’ working place, for example, “Community nurse” (*N* = 9; 8%). In total, 16% of the job titles (*N* = 19) provided information about the nurses’ education, for example, “Nurse practitioner” (*N* = 7; 6%) and 14% of the job titles (*N* = 16) included the nurses’ medical expertise, for example, “Geriatric nurse” (*N* = 5; 4%). Finally, 11% of the job titles (*N* = 13) provided multiple information, for example, “Community care registered nurse” (*N* = 2; 2%). As not much background information was provided about the nurses, it was not possible to determine their level of proficiency in terms of job titles. Also, titles were either not sufficiently specified, such as with “Nurse,” or applied differently in different countries, such as “Practice nurse,” and were not necessarily subject to the same regulations, which would have made further analysis problematic. However, the job titles “Nurse practitioner” and “Advanced Practice Nurse” seemed to be used more consistently and studies indicated that these nurses had more competencies and worked more independently. All nurses’ job titles are presented in Table 6.

**Table 6.** Nurses’ Job Titles Categorized by Information Provided.

Category	Details	*N*	%	*N* total	% total
Information about nurses’ function	Practice nurse	7	6	24	21
	Discharge nurse	2	2		
	Enablement officer	2	2		
	Nurse case manager	2	2		
	Nurse coordinator	2	2		
	Coordinator nurse	1	1		
	Experienced trained research nurse	1	1		
	Intervention nurse	1	1		
	Nurse researcher	1	1		
	Liaison nurse	1	1		
	Psychosocial nurse	1	1		
	Recruiting nurse	1	1		
	Research nurse	1	1		
	Staff nurse	1	1		
Information about nurses’ working place	Community nurse	9	8	23	20
	District nurse	5	4		
	Clinic nurse	1	1		
	Community care nurse	1	1		
	Community health nurse	1	1		
	Municipal nurse	1	1		
	Municipality nurse	1	1		
	Primary health care nurse	1	1		
	Primary care nurse	1	1		
	Public health nurse	1	1		
	Ward nurse	1	1		
Not specified	Nurse	22	19	22	19
Information about nurses’ education	Nurse practitioner (NP)	7	6	19	16
	Registered nurse (RN)	6	5		
	Advanced practice nurse (APN)	2	2		
	Certified nurse assistant (CNA)	1	1		
	Licensed practical nurse (LPN)	1	1		
	Advanced practice registered nurse (APRN)	1	1		
	Postgraduate nursing student	1	1		
Information about nurses’ medical expertise	Geriatric nurse	5	4	16	14
	Rehabilitation nurse	2	2		
	Respiratory nurse	2	2		
	Cardiac nurse	1	1		
	Cardiac rehabilitation nurse	1	1		
	Cardiovascular nurse	1	1		
	Asthma/COPD nurse	1	1		
	Heart failure nurse	1	1		
	Rheumatology nurse	1	1		
	Stroke nurse	1	1		
Multiple information	Community care registered nurse (CCRN)	2	2	13	11
	Registered practice nurse	1	1		
	Geriatric nurse practitioner	2	2		
	Gerontology nurse specialist	1	1		
	Advanced practice gerontic nurse (APGN)	1	1		
	Geriatric nurse specialist	1	1		
	Geriatric trained registered nurse	1	1		
	Respiratory nurse specialist	1	1		
	Registered nurse case manager (NCM)	1	1		
	Head nurse in the geriatric ward	1	1		
	Hospital case manager nurse	1	1		

*Note.* The percentages were calculated from *N* = 117 (total of job titles). COPD = chronic obstructive pulmonary disease.

### Nurses’ Competencies

The interventions were categorized with the updated ARN model (Vaughn et al., 2022) to determine which domains of rehabilitation nursing were covered and which nurses’ competencies were met. In the ARN updated model, the domains and competencies are separated with dotted lines because they overlap; thus, it was sometimes difficult to clearly assign the interventions. Out of 355 interventions provided by nurses, 341 interventions (96%) could be categorized in “Promotion of health and successful living” (domain 2) and 250 interventions (70%) in “Nurse-led interventions” (domain 1). In domain 1, nurses used supportive technology to improve quality of life (competency 1.1) through the provision and training in the use of assistive products. They implemented interventions based on best evidence (competency 1.2) through several assessments and restorative and compensatory interventions. They further provided patient and family education (competency 1.3) through counseling and skills training and delivered patient and family centered care (competency 1.5) through the assessment of person-centered goals and social care and support. In domain 2, nurses promoted health and prevented disability (competency 2.1) through assessments, restorative and compensatory interventions, social care, and support. They further fostered self-management (competency 2.2) through education and skills training for self-care and self-management, and education and skills training for caregivers. They also promoted and facilitated safe and effective care transitions (competency 2.3) through coordinating and manageing the rehabilitation process. However, no interventions could be categorized in “Leadership” (domain 3) and “Intra- and Interprofessional team” (domain 4) as no information was provided in the studies. The table containing the categorization of the nurses’ competencies in the model is available in Appendix B in the online supplementary file 3.

## Discussion

This secondary analysis of a scoping review (Seijas et al., 2024) synthesized information from 68 studies in HICs and upper-middle-income countries to map the role of nurses in providing rehabilitation interventions ageing populations in PHC. Participants had a mean age of 74 years and mostly received rehabilitation interventions due to a decline in functioning. In about half of the studies, nurses worked in multidisciplinary teams, mostly with physical therapists. No information was available on how the collaboration between the rehabilitation providers enfolded. In total, nurses provided 355 different rehabilitation interventions and mainly assessed person-centered goals and functioning and provided follow-up visits and case management. Nurses typically had a clinical and managerial role and led 38% of the interventions themselves. In 25% of the studies, nurses took over tasks from other health workers mainly as an enhancement of their role. Nurses had 117 different job titles and the most frequent one was simply “Nurse.” As not much background information was provided about the nurses, it was not possible to further determine their level of proficiency in terms of job titles. However, nurses with the job title “Advanced Practice Nurse” (APN) seemed to have more competencies and worked more independently. According to the updated ARN Model (Vaughn et al., 2022), 96% of the interventions could be assigned to “Promotion of health and successful living” (domain 2). In this role, nurses had the competencies to promote health and prevent disability, foster self-management, and promote and facilitate safe and effective care transitions. Finally, given increased need for rehabilitation in PHC and the central role of nurses in the provision of this health strategy for healthy ageing, further efforts in research and policy are necessary to better describe and sharpen their role considering the different levels of proficiency.

**This secondary analysis shows that nurses in rehabilitation mostly collaborate with physical therapists and take over tasks as an enhancement of their role; however, there was little information regarding the collaboration between rehabilitation providers, limiting the ability to accurately describe how and why tasks were shifted.** The literature states that the most effective rehabilitation outcomes are achieved through collaboration between multidisciplinary teams (Gutenbrunner et al., 2021; World Health Organization, 2017). This was met in half of our studies, and nurses collaborated most frequently with physical therapists. Physical therapists were also the most represented rehabilitation providers in the primary analysis at 45.9% (Seijas et al., 2024). This is not surprising, as physical therapists—in contrast to nurses (Dalley & Sim, 2001; Gutenbrunner et al., 2021)—are considered typical rehabilitation providers alongside occupational, and speech and language therapists, prosthetists and orthotists, psychologists, and physical and rehabilitation medicine doctors (World Health Organization, 2018a, 2019). Despite the close collaboration between physical therapists and nurses, nurses took on additional tasks from them, classified mostly as an enhancement of their role. Ultimately, these nurses held professional titles that usually require additional training, such as Nurse specialist (NS) (Coskun & Duygulu, 2022; Everink et al., 2017, 2018), Nurse practitioner (NP) (Everink et al., 2017, 2018), APN (Buurman et al., 2016; Finlayson et al., 2018), and Clinical nurse specialist (CNS) (Taube et al., 2018). Similarly, the literature states that nurses tend to take over tasks from other professions, mainly due to new (extended) roles (European Commission Directorate-General for Health and Food Safety, 2019). However, this study's classification of task shifting is somewhat limited, as there was not enough information on the nurses’ training, working place, and collaboration with other rehabilitation providers to distinguish whether nurses took over tasks because other professions were not available (as substitution) or because they had the competence to take on more tasks (enhancement) (European Commission Directorate-General for Health and Food Safety, 2019). In addition, task shifting only occurs when tasks are shifted to professions with a lower level of training (Van Tuyl et al., 2021), which is challenging to assess in the case of physical therapists and nurses. Finally, the lack of information on collaboration is also reflected in the nurses’ competency domains of the ARN model, as no interventions could be categorized into “Intra- and interprofessional team.” The reason for this could be that the included studies were mainly RCTs, which focused on the effectiveness of the interventions and omitted information on collaboration, as it was not their focus. Finally, as multidisciplinary rehabilitation teams are critical to patient outcomes, studies targeting a better understanding of the collaboration between nurses—as core element of rehabilitation programs (Gutenbrunner et al., 2021, 2022)—and other rehabilitation providers are needed.

**Nurses play an important role in providing access to health care and coordinating and manageing the rehabilitation process of ageing populations in PHC; thus, they are instrumental to achieving healthy ageing, UHC, and SDG3.** According to the literature, nurses are not yet fully recognized as integral rehabilitation providers and are often overlooked in many conceptual frameworks of rehabilitation (Gutenbrunner et al., 2022). This secondary analysis provides evidence that nurses are essential in rehabilitation for ageing populations in PHC. Through their assessments, they enable older people to access rehabilitation and receive individualized, patient- and family centered care. According to the literature, nurses can make a significant contribution to promoting access and equity in health care, but their potential can only be realized if the barriers that prevent them from taking advantage of the full scope of their education are removed (Flaubert et al., 2021; Nolan & Nolan, 1997). Their role in coordinating and manageing the rehabilitation process—which, for ageing populations, often involves multiple institutions at different levels of care—and guiding this often vulnerable group through the continuum of care is essential to provide integrated, coordinated, and continuous care (Camicia et al., 2014). In this sense, this study shows that nurses actively contribute to promoting healthy ageing. Also, according to the WHO, robust PHC is instrumental in achieving UHC and SDG3 (World Health Organization & United Nations Children’s Fund (UNICEF), 2018). Given the high number of studies included, this secondary analysis emphasizes that nurses are essential in PHC. The International Council of Nurses (ICN) describes that nurses can have a great impact on PHC because they work with families, educators, and community groups and know the patients, their families, their support structures, their health needs, and their personal and financial problems. They also know which services are available and which are lacking, meaning they have a holistic view not only of the patient but also of the services (International Council of Nurses (ICN), 2019). Although nurses seem to be best placed to inform policy, the competency domain “Leadership” of the updated ARN model is not yet fulfilled. Our results stress the importance of involving nurses in policy-making processes.

**The role of nurses in rehabilitation is diverse and includes various job titles and competencies; however, information on educational background was lacking, making it impossible to determine and further analyze nurses’ level of proficiency.** The number of nurses has grown over the past 100 years, and the profession has undergone great transformation (Gutenbrunner et al., 2021). The shift in nursing education from hospital-based diploma to graduate degrees has prepared nurses for higher-skill roles that have expanded their reach and influence (Flaubert et al., 2021). This secondary analysis highlights the current variety of job titles for nurses in rehabilitation and reflects the diverse roles that nurses can assume. Most strikingly, the most common job title, simply “nurse,” is often used indiscriminately, raising questions about whether the scope of practice matches the corresponding training and educational background. This secondary analysis was not able to extract enough information from the studies to explore this question in more detail, and, for example, to analyze the nurses’ competencies in the updated ARN model according to the three levels of proficiency (Vaughn et al., 2022). However, the results confirm that APNs often lead interventions and tend to work more independently and have enhanced competencies. This is consistent with current literature that sees great potential in including APNs in PHC (Pan American Health Organization (PAHO) & World Health Organization, 2018; European Observatory on Health Systems and Policies et al., 2019). For example, one included study provides evidence that the gerontology NS role—as one of the four types of APNs—was highly regarded by the ageing population and other health workers in PHC (King et al., 2018). Finally, given the diversity in the nurses’ role, it is important to harmonize competencies with level of proficiency so that nurses can continue to provide quality care. Studies could include more information on their background or specify their training, for example.

### Strengths and Limitations

Several limitations have been identified that should be considered when interpreting the results. One such limitation is that all the studies were conducted in HICs and upper-middle-income countries, which means that the findings cannot be generalized to a global scale. Additionally, the interventions took place within country-specific political and health systems, making it challenging to compare the roles of nurses across the studies as they are regulated differently across countries. Another limitation is that interventions often had multiple components that were delivered by different health workers, which were not always differentiated, making it hard to extract interventions that were “only” provided by nurses. Another limitation is the categorization of substitution in task shifting, as not much information was available about the collaboration between the health workers and the nurses’ level of proficiency. Finally, the additional data extraction for this secondary analysis was performed by the first author (VL) and double-checked but not independently extracted by the second author (VS). This lack of double and independent data extraction may have resulted in author bias.

### Implications for Practice

This study contributes to a better understanding of the key role nurses play in providing rehabilitation interventions to ageing populations in primary health care. The results show the heterogeneity in nurses’ titles and highlight the importance of multidisciplinary teams in rehabilitation to meet the needs of ageing populations. Recognizing the nurses’ roles in rehabilitation and promoting them more clearly as potential career opportunities for nurses may benefit recruitment and retention in this area of work. It is now important to harmonize nurses’ competencies with their level of proficiency to ensure quality of care in rehabilitation.

## Conclusion

With the ageing of the global population and the rise of NCDs, rehabilitation nursing has become increasingly important in PHC. This secondary analysis addresses the urgent need to better understand and promote the role of rehabilitation nurses in this level of care and to foster their collaboration with other health professionals to meet the specific needs of ageing populations.

## Supplemental Material

sj-docx-2-son-10.1177_23779608241271677 - Supplemental material for The Role of Nurses in Rehabilitation in Primary Health Care for Ageing Populations: A Secondary Analysis from a Scoping Review Supplemental material, sj-docx-2-son-10.1177_23779608241271677 for The Role of Nurses in Rehabilitation in Primary Health Care for Ageing Populations: A Secondary Analysis from a Scoping Review by Viola Lorenz, Vanessa Seijas, Heidrun Gattinger, Claudia Gabriel, Margrieta Langins, Satish Mishra and Carla Sabariego in SAGE Open Nursing

sj-docx-3-son-10.1177_23779608241271677 - Supplemental material for The Role of Nurses in Rehabilitation in Primary Health Care for Ageing Populations: A Secondary Analysis from a Scoping Review Supplemental material, sj-docx-3-son-10.1177_23779608241271677 for The Role of Nurses in Rehabilitation in Primary Health Care for Ageing Populations: A Secondary Analysis from a Scoping Review by Viola Lorenz, Vanessa Seijas, Heidrun Gattinger, Claudia Gabriel, Margrieta Langins, Satish Mishra and Carla Sabariego in SAGE Open Nursing

sj-docx-4-son-10.1177_23779608241271677 - Supplemental material for The Role of Nurses in Rehabilitation in Primary Health Care for Ageing Populations: A Secondary Analysis from a Scoping Review Supplemental material, sj-docx-4-son-10.1177_23779608241271677 for The Role of Nurses in Rehabilitation in Primary Health Care for Ageing Populations: A Secondary Analysis from a Scoping Review by Viola Lorenz, Vanessa Seijas, Heidrun Gattinger, Claudia Gabriel, Margrieta Langins, Satish Mishra and Carla Sabariego in SAGE Open Nursing

sj-docx-5-son-10.1177_23779608241271677 - Supplemental material for The Role of Nurses in Rehabilitation in Primary Health Care for Ageing Populations: A Secondary Analysis from a Scoping Review Supplemental material, sj-docx-5-son-10.1177_23779608241271677 for The Role of Nurses in Rehabilitation in Primary Health Care for Ageing Populations: A Secondary Analysis from a Scoping Review by Viola Lorenz, Vanessa Seijas, Heidrun Gattinger, Claudia Gabriel, Margrieta Langins, Satish Mishra and Carla Sabariego in SAGE Open Nursing

sj-docx-6-son-10.1177_23779608241271677 - Supplemental material for The Role of Nurses in Rehabilitation in Primary Health Care for Ageing Populations: A Secondary Analysis from a Scoping Review Supplemental material, sj-docx-6-son-10.1177_23779608241271677 for The Role of Nurses in Rehabilitation in Primary Health Care for Ageing Populations: A Secondary Analysis from a Scoping Review by Viola Lorenz, Vanessa Seijas, Heidrun Gattinger, Claudia Gabriel, Margrieta Langins, Satish Mishra and Carla Sabariego in SAGE Open Nursing

sj-docx-7-son-10.1177_23779608241271677 - Supplemental material for The Role of Nurses in Rehabilitation in Primary Health Care for Ageing Populations: A Secondary Analysis from a Scoping Review Supplemental material, sj-docx-7-son-10.1177_23779608241271677 for The Role of Nurses in Rehabilitation in Primary Health Care for Ageing Populations: A Secondary Analysis from a Scoping Review by Viola Lorenz, Vanessa Seijas, Heidrun Gattinger, Claudia Gabriel, Margrieta Langins, Satish Mishra and Carla Sabariego in SAGE Open Nursing

sj-docx-8-son-10.1177_23779608241271677 - Supplemental material for The Role of Nurses in Rehabilitation in Primary Health Care for Ageing Populations: A Secondary Analysis from a Scoping Review Supplemental material, sj-docx-8-son-10.1177_23779608241271677 for The Role of Nurses in Rehabilitation in Primary Health Care for Ageing Populations: A Secondary Analysis from a Scoping Review by Viola Lorenz, Vanessa Seijas, Heidrun Gattinger, Claudia Gabriel, Margrieta Langins, Satish Mishra and Carla Sabariego in SAGE Open Nursing

sj-docx-9-son-10.1177_23779608241271677 - Supplemental material for The Role of Nurses in Rehabilitation in Primary Health Care for Ageing Populations: A Secondary Analysis from a Scoping Review Supplemental material, sj-docx-9-son-10.1177_23779608241271677 for The Role of Nurses in Rehabilitation in Primary Health Care for Ageing Populations: A Secondary Analysis from a Scoping Review by Viola Lorenz, Vanessa Seijas, Heidrun Gattinger, Claudia Gabriel, Margrieta Langins, Satish Mishra and Carla Sabariego in SAGE Open Nursing

sj-docx-10-son-10.1177_23779608241271677 - Supplemental material for The Role of Nurses in Rehabilitation in Primary Health Care for Ageing Populations: A Secondary Analysis from a Scoping Review Supplemental material, sj-docx-10-son-10.1177_23779608241271677 for The Role of Nurses in Rehabilitation in Primary Health Care for Ageing Populations: A Secondary Analysis from a Scoping Review by Viola Lorenz, Vanessa Seijas, Heidrun Gattinger, Claudia Gabriel, Margrieta Langins, Satish Mishra and Carla Sabariego in SAGE Open Nursing

sj-docx-11-son-10.1177_23779608241271677 - Supplemental material for The Role of Nurses in Rehabilitation in Primary Health Care for Ageing Populations: A Secondary Analysis from a Scoping Review Supplemental material, sj-docx-11-son-10.1177_23779608241271677 for The Role of Nurses in Rehabilitation in Primary Health Care for Ageing Populations: A Secondary Analysis from a Scoping Review by Viola Lorenz, Vanessa Seijas, Heidrun Gattinger, Claudia Gabriel, Margrieta Langins, Satish Mishra and Carla Sabariego in SAGE Open Nursing
