# Supplementary material for: The Role of Nurses in Rehabilitation in Primary Health Care for Ageing Populations: A Secondary Analysis from a Scoping Review
Source: SAGE Open Nurs. 2024 Sep 23;10:23779608241271677. doi: 10.1177/23779608241271677 (PMC11425760; doi:10.1177/23779608241271677)
Supplement: sj-docx-2-son-10.1177_23779608241271677 - Supplemental material for The Role of Nurses in Rehabilitation in Primary Health Care for Ageing Populations: A Secondary Analysis from a Scoping Review [file sj-docx-2-son-10.1177_23779608241271677.docx]

**The Role of Nurses in Rehabilitation in Primary Health Care for Ageing Populations: A Secondary Analysis from a Scoping Review**

**List of abbreviations**

| ARN | Association of Rehabilitation Nurses |
| --- | --- |
| ANA | American Nurses Association |
| FA | Functional ability |
| IC | Intrinsic capacity |
| HICs | High-income countries |
| ICF | International Classification of Functioning |
| IC | Intrinsic capacity |
| ICN | International Council of Nurses |
| LMICs | Low- and middle-income countries |
| NCDs | Non-communicable diseases |
| PHC | Primary Health Care |
| PRM | Physical and rehabilitation medicine |
| SDG3 | Sustainable Development Goal 3 |
| UN | United Nations |
| UHC | Universal Health Coverage |
| WHO | World Health Organization |
