# Supplementary material for: The Role of Nurses in Rehabilitation in Primary Health Care for Ageing Populations: A Secondary Analysis from a Scoping Review
Source: SAGE Open Nurs. 2024 Sep 23;10:23779608241271677. doi: 10.1177/23779608241271677 (PMC11425760; doi:10.1177/23779608241271677)
Supplement: sj-docx-3-son-10.1177_23779608241271677 - Supplemental material for The Role of Nurses in Rehabilitation in Primary Health Care for Ageing Populations: A Secondary Analysis from a Scoping Review [file sj-docx-3-son-10.1177_23779608241271677.docx]

**The Role of Nurses in Rehabilitation in Primary Health Care for Ageing Populations: A Secondary Analysis from a Scoping Review**

**Supplement material titles**

Appendix A, Supplementary file 1: “Study Protocol from primary scoping review”

Appendix A, Supplementary file 2: “Preferred Reporting Items for Systematic reviews and Meta-Analyses extension for Scoping Reviews (PRISMA-ScR) Checklist”

Appendix A, Supplementary file 3: “Study Protocol”

Appendix A, Supplementary file 4: “Search concepts, terms, and search strategies for each bibliographic database queried”

Appendix A, Supplementary file 5: “Data extraction form”

Appendix B, Supplementary file 1: “Overview of all included studies”

Appendix B, Supplementary file 2: “Nurses’ training”

Appendix B, Supplementary file 3: “Nurses domains and competencies in ARN Modell”
