# Supplementary material for: The Role of Nurses in Rehabilitation in Primary Health Care for Ageing Populations: A Secondary Analysis from a Scoping Review
Source: SAGE Open Nurs. 2024 Sep 23;10:23779608241271677. doi: 10.1177/23779608241271677 (PMC11425760; doi:10.1177/23779608241271677)
Supplement: sj-docx-6-son-10.1177_23779608241271677 - Supplemental material for The Role of Nurses in Rehabilitation in Primary Health Care for Ageing Populations: A Secondary Analysis from a Scoping Review [file sj-docx-6-son-10.1177_23779608241271677.docx]

***Appendix A Supplementary file 3***

**Study protocol**

**The Role of Nurses in the Provision of Rehabilitation Interventions to the Ageing Population in Primary Health Care:
A Secondary Analysis as a Scoping Review**

Last update: 12.02.2024

**List of abbreviations**

| Association of Rehabilitation Nurses | ARN |
| --- | --- |
| Functional ability | FA |
| Intrinsic capacity | IC |
| High-income countries | HICs |
| International Classification of Functioning | ICF |
| Intrinsic capacity | IC |
| International Council of Nurses | ICN |
| Lower-middle income countries | LMICs |
| Non-communicable diseases | NCDs |
| Primary Health Care | PHC |
| Sustainable Development Goals | SDGs |
| United Nations | UN |
| Universal Health Coverage | UHC |
| World Health Organization | WHO |

# **Introduction**

This protocol aims to provide a “road map” for a scoping review, which will be the secondary analysis of the yet unpublished scoping review “Healthy ageing and rehabilitation: Service delivery models used to optimize intrinsic capacity and functional ability”. The proposed review will use and analyze the primary data of this previous review with a narrower thematic focus. This first chapter is about the rationale of the proposed review, its objective, and the definitions that will be considered.

## **Rationale**

The world is currently facing two rapid trends. First, the population is ageing worldwide: in 2021, the proportion of people aged 65 and over was 10% and is expected to rise to 16% by 2050. This has been observed for several years in high-income countries (HICs), which is why they still account for the highest numbers of older people. It is of particular concern that the increase is also occurring in low- and middle-income countries (LMIC), even at a faster pace (United Nations, 2022). At the same time, the incidence and prevalence of non-communicable diseases (NCDs) are also rising. NCDs – namely cardiovascular disease, cancer, chronic respiratory disease, and diabetes are currently the largest contributors to mortality and disability worldwide (World Health Organization, 2022c). According to the World Bank, they account for 74% of all deaths worldwide, with 88% in HICs and 70% in LMICs in 2020 (World Bank, 2020). LMICs also have a high prevalence of communicable diseases, resulting in a double burden of disease (World Health Organization, 2022c). These two trends affect all aspects of societies and require health systems to adapt the delivery of health services to meet the changing needs of the ageing population.

Ageing is associated with a decline of functional ability (FA) and requires care aimed at optimizing it to reduce disability. FA (also called *“functioning”*) results from the interaction of a person’s intrinsic capacity (IC) – all the mental and physical abilities – with their environment (World Health Organization, 2015). It is embodied in the definition of rehabilitation as *“a set of interventions designed to optimize functioning and reduce disability in individuals with health conditions in interaction with their environment”* (World Health Organization, 2017, p. 1). The decline of FA can manifest as an acute or as a subacute insidious condition. In the ageing population, the latter is often not recognized until it is at an advanced stage, when it is difficult to manage and costly for health systems (Fong, 2019; World Health Organization, 2015). In response to this, the WHO and the UN have launched the *“Decade of Healthy Ageing 2020-2030*” (2022) and called for global collaboration to promote healthy ageing. As defined by the WHO, healthy aging is *“the process of developing and maintaining the functional ability that enables well-being in old age”* (World Health Organization, 2015, p. 28). In line with this definition, the WHO recognizes rehabilitation as a tool for achieving healthy aging by focusing on the optimization of FA. Therefore, rehabilitation must be integrated into all levels of care to be accessible to the ageing population.

Primary health care (PHC) is at the heart of health systems and represents the level of care at which health services are coordinated with one another. It is considered the gateway to health care because through it, people can access specialized health services (World Health Organization, 2018b). Through its central role, a strong PHC is *“the most inclusive and cost-effective way”* to achieve Universal Health Coverage (UHC) and the Sustainability Development Goals (SGDs) (World Health Organization & United Nations Children’s Fund, 2019, p. 17). PHC includes various health services from *“health promotion to disease prevention, treatment, rehabilitation, palliative care, and more”* (World Health Organization & United Nations Children’s Fund, 2018, p. 12). Although rehabilitation is considered an essential health service at this level (World Health Organization, 2015), it is still insufficiently integrated because some misconceptions persist, such as *“a fallback strategy when preventive or curative interventions fail, a luxury or optional health service for those who can afford it, and only disability-specific”* (World Health Organization, 2018a, p. 9). Ultimately, the WHO advocates for the integration of rehabilitation into PHC and calls on countries to prioritize it in their health policy agenda.

To achieve desired outcomes, rehabilitation services must be provided by multidisciplinary teams in which nurses play an essential role (European Observatory on Health Systems and Policies et al., 2019; World Health Organization, 2018a). Nurses are the largest professional group in healthcare systems, accounting for over 50% of all healthcare workers worldwide. Their initial clinical assessment forms the basis for further treatment and is critical to health care delivery, as they are often the only health professional a patient sees (World Health Organization et al., 2020). Regarding the principle of teamwork in rehabilitation, it is of great importance to describe the core competencies and roles of professionals with different educational backgrounds. A clear role definition is necessary to avoid overlap, and insufficient or inappropriate use of the different professional groups' competencies. Currently, there is a gap between the imminent relevance and evaluation of the role of nursing in rehabilitation (Gutenbrunner et al., 2021; Nolan & Nolan, 1999). Rehabilitation nursing has been organized as a specialty in 1964. Since then, several concepts and frameworks have been developed (Gutenbrunner et al., 2021), including the ARN Strategic Plan 2022-2025 launched in 2022, with the vision of improving health care delivery by integrating rehabilitation nursing concepts across the continuum of care (Association of Rehabilitation Nursing, 2022a). However, the nurse’s specific role in providing rehabilitation in PHC to the ageing population is not yet well defined and needs to be better understood.

## **Objective**

Therefore, this review builds on the findings of the primary review and aims to describe the role of nurses in the provision of rehabilitation interventions to the ageing population in PHC. To our knowledge, it is the first review to address this knowledge gap. It will explore the following research question: “Which role do nurses have in the provision of rehabilitation interventions to the ageing population in primary health care?”. To focus on the research question, the Population, Concept, and Context (PCC) framework recommended by Joana Bridge Institute (Peters et al., 2020) is elaborated as follows:

- **P**opulation: Ageing population
- **C**oncept: Nurses’ role in providing rehabilitation interventions
- **C**ontext: Primary health care and community-based care

## **Definitions**

In the primary analysis, most of the terms were defined to have a common understanding. This review considers all previous terms equally to ensure consistency and adds new definitions in line with the PCC framework. The new definitions are provided in appendix B, whereas the terms *“healthy ageing”*, *“rehabilitation”* and *“FA”* have already been defined in the introduction.

# **Methods**

This second chapter deals with the methods of the proposed review, which will be conducted in accordance with the JBI methodology for scoping reviews (Peters et al., 2020) and the Preferred Reporting Items for Systematic Reviews and Meta-Analyses Protocols (PRISMA-P) Statement (Tricco et al., 2018). To increase research transparency and avoid duplicates, it has been registered in the Open Science Forum (OSF). As no human subjects will be involved, no ethical approval is required.


## **Methods of the primary analysis**

*Title:* Healthy ageing and rehabilitation: Service delivery models used to optimize intrinsic capacity and functional ability – a scoping review

*Research questions:* Which rehabilitation services are the most relevant to the ageing population? How should they be delivered or who could benefit from them?

*Objective:* To provide an overview of rehabilitation service delivery models used to optimize the ageing population's IC and FA.

*Study design:* Scoping Review according to Preferred Reporting Items for Systematic Reviews and Meta-Analyses extension for Scoping Reviews (PRISMA-ScR) (Tricco et al., 2018)

*Study protocol:* Yes, available.

*Eligibility criteria*: They are mentioned in this protocol in chapter 2.2

*Information source and search strategy:* a comprehensive search strategy was designed by the four authors considering the extensive scope of rehabilitation and substantial difference in how it is understood across countries and settings. The structured search included natural language and Medical Subject Headings, grouped into three concepts: (1) rehabilitation, ICF and FA (2) models of care or health care approaches, and (3) ageing population. Evidence for optimal database combinations was used and the search was conducted in MEDLINE and EMBASE.

*Study selection process*: Only a random sample of 35% of the retrieved records was screened, because of the very high number of included publications. Two researchers and a student assistant independently screened abstracts using Rayyan. 50% of the records were double screened. Training sessions and team meetings were held to ensure consistency in the decision process and to clarify eligibility criteria and discuss open issues. In total, three training rounds were required. Then, three researchers and a student assistant screened the included publications: all full texts were assessed independently and double-checked. Disagreements were resolved in meetings with a third team member.

*Data extraction process*: Four conceptual frameworks (Gutenbrunner et al., 2020; The Cochrane Effective Practice and Organisation of Care (EPOC) group, 2015; World Health Organization, 2022a, 2022b) and input from rehabilitation and health systems research experts were used to develop the data extraction form, which included information regarding studies characteristics, target population, rehabilitation service delivery and rehabilitation interventions. The data extraction process began only after a high agreement (>90%). Three researchers and a student assistant extracted data independently and double-checked the extraction of others. In line with the methodology of scoping review, no quality appraisal or risk of bias was carried out. The goal was to identify service delivery models rather than to assess their effectiveness.

*Data synthesis*: Quantitative analysis (e.g. frequencies) of study characteristics, rehabilitation interventions, and rehabilitation services provision and qualitative analysis to identify rehabilitation service delivery models were carried out.

*Patient and public involvement*: No, has not been included.

## **Study design**

The study design has been chosen following Kazi et al. (2021) which states that scoping reviews are a useful method for mapping data by selected key topics, enabling broad capture of information and identifying research gaps in the literature.

## **Eligibility criteria**

All inclusion and exclusion criteria are listed in Table 1, with the criteria from the primary analysis and new criteria (marked with *) classified according to the PCC format.

Table 1: Inclusion and exclusion criteria according to the PCC format

|  | Inclusion criteria | Exclusion criteria |
| --- | --- | --- |
| Population | | |
| Ageing population | Study population with a mean age of 50 years or over. The evidence that countries with similar levels of age-related burden experience different onsets of ageing is considered. When the mean age was not available, studies that target multiple age-related diseases, whose incidence rates increase quadratically with age were included (Chang et al., 2019). Diseases from selected clusters defined by Kuan et al. (2021) were used. |  |
| Concept | | |
| Nurses’ role in providing rehabilitation interventions* | Studies with models of care, service delivery methods, mode of service delivery, care services, care programs, organization of care. | Studies that focus on the description of needs, functional patterns, disability, risk factors, or protective factors of the ageing population. |
|  | Studies with the goal to enable healthy ageing through improving FA and IC and reducing the experience of disability of the ageing population living with a health condition. | Studies that focus only on other types of outcomes like morbidity, mortality, disease control related outcomes, interventions adherence, interventions' perceived quality, and willingness to continue, enjoyment, participation, health service's use, caregivers’ burden, implementation barriers or health workers perceptions. |
|  | Studies with interventions provided by nurses across all levels of education, working alone or in teams with other healthcare workers or people in the community. | Studies with interventions provided by other health workers or people working alone or in teams without nurses. |
| Context | | |
| Primary health care and Community-based care* | Studies with interventions taking place in the community or at the primary health care level or combine one of these levels with specialized ones to include transitions from one level to another. | Studies with interventions taking place only in a specialized care setting. |
| Study characteristics | | |
| Study type | Primary studies, interventional, observational, or descriptive. | Other studies like books, book chapters, narrative reviews, systematic reviews, meta-analyses, position papers, guidelines or recommendations, letters to the editor, conference proceedings, retraction letters. |
| Language | Studies that are published or translated in English. | Studies that are not available in English. |
| Publication year | Studies that were published between January 2015 until May 2022. | Studies that were published before January 2015 or after May 2022. |

## **Information sources and search strategy**

Since this review will be a secondary analysis of already collected data, no new search strategy will be developed, and no new literature search will be conducted. The summary of the search concepts and terms and the study selection process of the primary analysis is attached in Figure 1 and 2 in appendix A.

## **Study selection process**

The selection of studies will take place in an Excel data extraction form similar to the one from the primary analysis. To restrict the studies according to the new eligibility criteria, two filtering processes will be made. First, the variable *“Level of care”* will be filtered by *“Multiple levels of care”* and *“Primary health care”.* Then, the variable *“Health care workers”* will be filtered by *“Nurses”*. Only the studies resulting from these two filtering processes will be selected.

The author will then review all titles and abstracts of the selected studies to ensure that they align with the criteria. In cases of doubt, the studies will be marked and discussed with the main authors of the primary review to reach consent (100%).

## **Data extraction process**

New data will be extracted by one author in an Excel data extraction form containing the variables and results from the primary analysis. Two frameworks have been used to define new variables. For PHC, the “*WHO Operational Framework for Primary Health Care Transforming Vision into Action”* (World Health Organization & United Nations Children’s Fund, 2020) was considered and for the role of nurses, the “*Professional Rehabilitation Nursing Competency Model”* (Vaughn et al., 2016). However, the latest one has only served as inspiration, as the aim of this review is not to compare the role, but to describe how it unfolds in the rehabilitation interventions. In total, there will be around 90 variables (including those from the primary analysis), which are divided into four main sections in Table 2. In this, the new variables are marked with *.

Table 2: Variables for the data extraction form

| Section 1: General characteristics of the studies | |
| --- | --- |
| Variables | Key, PMID, Title, Publication year, Journal, Authors, Abstract, Country, Study design, Study design (category) |
| Section 2: Characteristics of the studies’ target population | |
| Variables | Target population, Target population (category), Health condition, Health condition area, Age-related inclusion (transformed), Mean age (transformed), Number of participants, Gender (% male), Participants' sex predominance (transformed) |
| Section 3: Characteristics of the rehabilitation interventions in PHC | |
| Variables | Description of the intervention, Intervention summary, Intervention type, Rural or urban?*, Co-design of intervention*, How was the intensity of rehabilitation decided?*, Name of intervention*, Single or multiple types of sessions?*, Session 1*, Session 2*, Session 3*, Session 4*, Session 5*, Session 6*, Session 7*, Session 8*, Time session 1 (in min)*, Time session 2 (in min)*, Time session 3 (in min)*, Time session 4 (in min)*, Time session 5 (in min)*, Time session 6 (in min)*, Time session 7 (in min)*, Time session 8 (in min)*, Time of the longest session (in minutes)*, Average of sessions' time (in minutes)*, Frequency session 1 (per week)*, Frequency session 2 (per week)*, Frequency session 3 (per week)*, Frequency session 4 (per week)*, Frequency session 5 (per week)*, Frequency session 6 (per week)*, Frequency session 7 (per week)*, Frequency session 8 (per week)*, Average of sessions' frequency*, Frequency of the most frequent session (per week)*, Grouped version of all sessions' duration (in minutes) with the frequency (per week)*, Total number of sessions that each patient received*, Total duration of the intervention (in weeks)*, Comments on time and intensity, Paper’s conclusion, Paper's conclusion (transformed), Outcomes, Rehabilitation provider, Multidisciplinary rehabilitation team, Self-management, Health worker rehabilitation provider (filtered)*, Level of care (filtered)*, Mode of service delivery, Aim healthy ageing?, Integrated care?, Role or task shifting, Role or task shifting revised* |
| Section 4: Characteristics of the nurses’ role | |
| Variables | Nurses’ role*, Nurses’ interventions*, Nurses’ role (transformed)*, Nurses’ communication*, Nurses’ title or designation*, Nurses’ training*, Nurses’ work independence*, Additional information about the nurses' role*, Notes, Comments (Examples)* |

In general, all variables from the primary analysis will be included. However, the variable *Role or task shifting”* will be completely revised with a focus on nurses. If further important aspects about the role of nurses emerge during data extraction that cannot be classified into the predefined variables, they will be included in *“Additional information about the nurses’ role”*.

Finally, no critical appraisal of the selected studies will be done because the objective of the review is not to assess the effectiveness of interventions and, therefore, the assessment of study bias is not critical to the findings of this review.

## **Data synthesis**

The synthesis of the data will include quantitative and qualitative analysis. Descriptive statistics (frequencies) will be made in Excel for the studies’ characteristics, studies’ target population, rehabilitation interventions in PHC and nurses’ characteristics. The role of the nurses will be analyzed more in depth with an inductive, basic qualitative content approach in Excel.

## **Data analysis**

The results of the quantitative and qualitative data will be presented in four sections:

1. Characteristics of the included studies
2. Characteristics of the studies’ target population
3. Characteristics of the rehabilitation interventions in PHC
4. Characteristics of the nurses’ role

Each chapter will contain a narrative text accompanied by tables.

**References**

Andrews, M. (2004). *A glossary of terms for community health care and services for older persons* (No. 5; Ageing and Health Technical Report). WHO Center for Health Development.

Association of Rehabilitation Nursing. (2022a). *ARN Strategic Plan 2022-2025*. https://rehabnurse.org/about/strategic-plan

Association of Rehabilitation Nursing. (2022b). *Strategic Plan*. Association of Rehabilitation Nurses. https://rehabnurse.org/about/strategic-plan

Chang, A. Y., Skirbekk, V. F., Tyrovolas, S., Kassebaum, N. J., & Dieleman, J. L. (2019). Measuring population ageing: An analysis of the Global Burden of Disease Study 2017. *The Lancet. Public Health*, *4*(3), e159–e167. https://doi.org/10.1016/S2468-2667(19)30019-2

EPOC Taxonomy. (n.d.). *Effective Practice and Organisation of Care (EPOC)*. Retrieved August 17, 2022, from https://epoc.cochrane.org/epoc-taxonomy

European Observatory on Health Systems and Policies, Sottas, B., Josi, R., Gysin, S., & Essig, S. (2019). Implementing Advanced Practice Nurses in Swiss Primary Care. *Eurohealth*, *25*(4), 25–28.

Fong, J. H. (2019). Disability incidence and functional decline among older adults with major chronic diseases. *BMC Geriatrics*, *19*(1), 323. https://doi.org/10.1186/s12877-019-1348-z

Gutenbrunner, C., Nugraha, B., Gimigliano, F., Meyer, T., & Kiekens, C. (2020). International Classification of Service Organization in Rehabilitation: An updated set of categories (ICSO-R 2.0). *Journal of Rehabilitation Medicine*, *52*(1). https://doi.org/10.2340/16501977-2627

Gutenbrunner, C., Stievano, A., Stewart, D., Catton, H., & Nugraha, B. (2021). Role of Nursing in Rehabilitation. *Journal of Rehabilitation Medicine - Clinical Communications*, *4*, 1000061. https://doi.org/10.2340/20030711-1000061

International Council of Nurses. (1987). *Nursing Definitions*. ICN - International Council of Nurses. https://www.icn.ch/nursing-policy/nursing-definitions

International Council of Nurses. (2002). *Nursing Definitions*. ICN - International Council of Nurses. https://www.icn.ch/nursing-policy/nursing-definitions

Kazi, M., Chowdhury, N., Chowdhury, M., & Turin, T. (2021). Conducting comprehensive scoping reviews to systematically capture the landscape of a subject matter. *Population Medicine*, *3*(December), 1–9. https://doi.org/10.18332/popmed/143831

Kuan, V., Fraser, H. C., Hingorani, M., Denaxas, S., Gonzalez-Izquierdo, A., Direk, K., Nitsch, D., Mathur, R., Parisinos, C. A., Lumbers, R. T., Sofat, R., Wong, I. C. K., Casas, J. P., Thornton, J. M., Hemingway, H., Partridge, L., & Hingorani, A. D. (2021). Data-driven identification of ageing-related diseases from electronic health records. *Scientific Reports*, *11*(1), Article 1. https://doi.org/10.1038/s41598-021-82459-y

Nolan, M., & Nolan, J. (1999). Rehabilitation, chronic illness and disability: The missing elements in nurse education. *Journal of Advanced Nursing*, *29*(4), 958–966. https://doi.org/10.1046/j.1365-2648.1999.00972.x

Peters, M., Godfrey, C., McInerney, P., Munn, Z., Tricco, A., & Khalil, H. (2020). *Chapter 11: Scoping Reviews (2020 version)*. JBI. https://synthesismanual.jbi.global.

The Cochrane Effective Practice and Organisation of Care (EPOC) group. (2015). *Effective Practice and Organisation of Care Taxonomy*. https://epoc.cochrane.org/epoc-taxonomy

Tricco, A. C., Lillie, E., Zarin, W., O’Brien, K. K., Colquhoun, H., Levac, D., Moher, D., Peters, M. D. J., Horsley, T., Weeks, L., Hempel, S., Akl, E. A., Chang, C., McGowan, J., Stewart, L., Hartling, L., Aldcroft, A., Wilson, M. G., Garritty, C., … Straus, S. E. (2018). PRISMA Extension for Scoping Reviews (PRISMA-ScR): Checklist and Explanation. *Annals of Internal Medicine*, *169*(7), 467–473. https://doi.org/10.7326/M18-0850

*UHC Compendium*. (2023). https://www.who.int/universal-health-coverage/compendium

United Nations. (2022). *World Population Prospects 2022: Summary of Results*. Department of Economic and Social Affairs Population Division. https://reliefweb.int/report/world/world-population-prospects-2022-summary-results

United Nations, & World Health Organization. (2022). *Decade of Healthy Ageing (2021-2030)*. https://www.who.int/initiatives/decade-of-healthy-ageing

Vaughn, S., Mauk, K. L., Jacelon, C. S., Larsen, P. D., Rye, J., Wintersgill, W., Cave, C. E., & Dufresne, D. (2016). The Competency Model for Professional Rehabilitation Nursing. *Rehabilitation Nursing*, *41*(1), 33–44. https://doi.org/10.1002/rnj.225

World Bank. (2020). *Derived based on the data from Global Health Estimates 2020: Deaths by Cause, Age, Sex, by Country and by Region, 2000-2019. Geneva, World Health Organization; 2020.* who.int/data/gho/data/themes/mortality-and-global-health-estimates/ghe-leading-causes-of-death

World Health Organization. (2001). *International Classification of Functioning, Disability and Health (ICF)*. https://www.who.int/standards/classifications/international-classification-of-functioning-disability-and-health

World Health Organization. (2015). *World report on ageing and health* (p. 260). Geneva World Health Organization.

World Health Organization. (2017). *Rehabilitation in Health Systems* (p. 92).

World Health Organization. (2018a). *Access to rehabilitation in primary health care: An ongoing challenge* (Technical Series on Primary Health Care, p. 32).

World Health Organization. (2018b). *Integrated Care for Older People: Realigning primary health care to respond to population ageing* (Technical Series on Primary Health Care, p. 24).

World Health Organization. (2019). *Glossary of terms: WHO European Primary Health Care Impact, Performance and Capacity Tool (PHC-IMPACT)* (WHO European Framework for Action on Integrated Health Services Delivery).

World Health Organization. (2022a). *International Classification of Health Interventions (ICHI)*. https://icd.who.int/dev11/l-ichi/en

World Health Organization. (2022b). *Universal Health Coverage Compendium*. https://www.who.int/universal-health-coverage/compendium/

World Health Organization. (2022c). *World health statistics 2022: Monitoring health for the SDGs, sustainable development goals*. World Health Organization. https://apps.who.int/iris/handle/10665/356584

World Health Organization. (2023a). *Primary care*. Integrated Health Services. https://www.who.int/teams/integrated-health-services/clinical-services-and-systems/primary-care

World Health Organization. (2023b). *Primary health care*. https://www.who.int/health-topics/primary-health-care

World Health Organization, Nursing Now, & International Council of Nurses. (2020). *State of the world’s nursing 2020: Investing in education, jobs and leadership*. https://www.who.int/publications-detail-redirect/9789240003279

World Health Organization, & United Nations Children’s Fund. (2018). *A vision for primary health care in the 21st century: Towards universal health coverage and the Sustainable Development Goals* (Technical Series on Primary Health Care, p. 64).

World Health Organization, & United Nations Children’s Fund. (2019). *Report of the Global Conference on Primary Health Care: From Alma-Ata towards Universal Health Coverage and the Sustainable Development Goals* (Global Conference on Primary Health Care, p. 74).

World Health Organization, & United Nations Children’s Fund. (2020). *Operational Framework for Primary Health Care Transforming Vision Into Action* (Technical Series on Primary Health Care, p. 132).

**Appendix**

1. **Methods of the primary analysis**


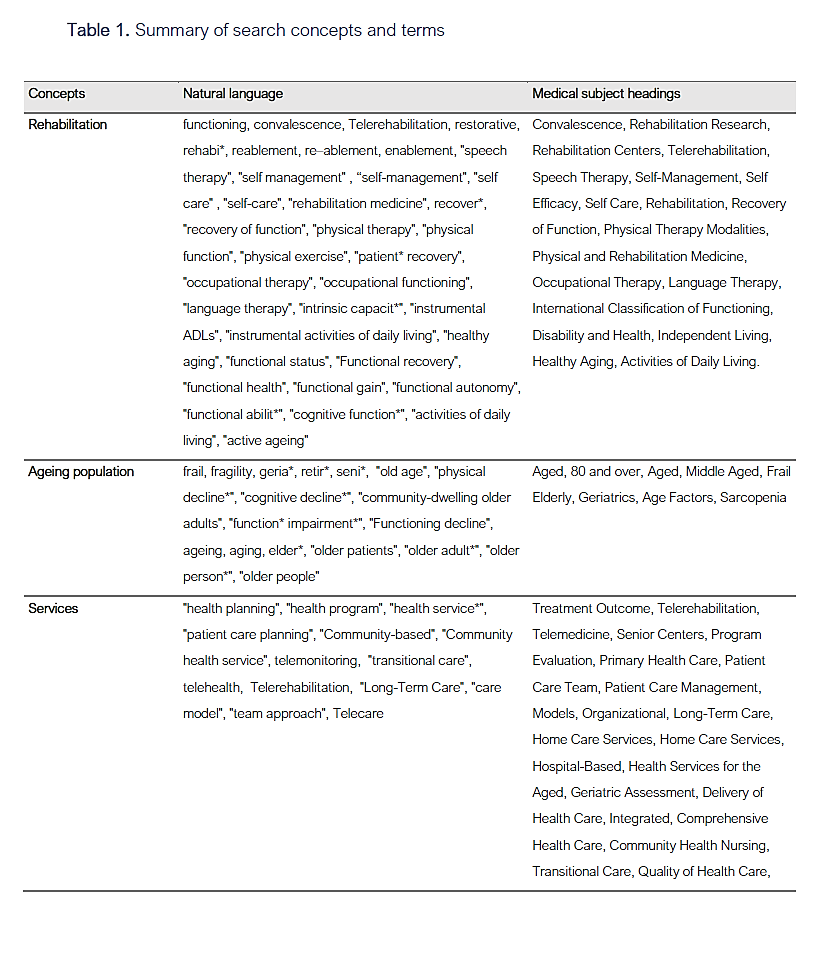
Figure 1: Summary of search concepts and terms of the primary analysis

**Services (continued)**


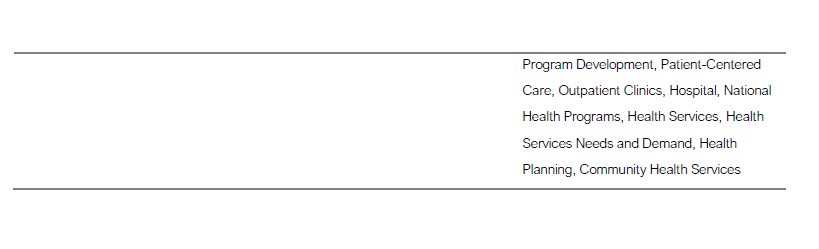


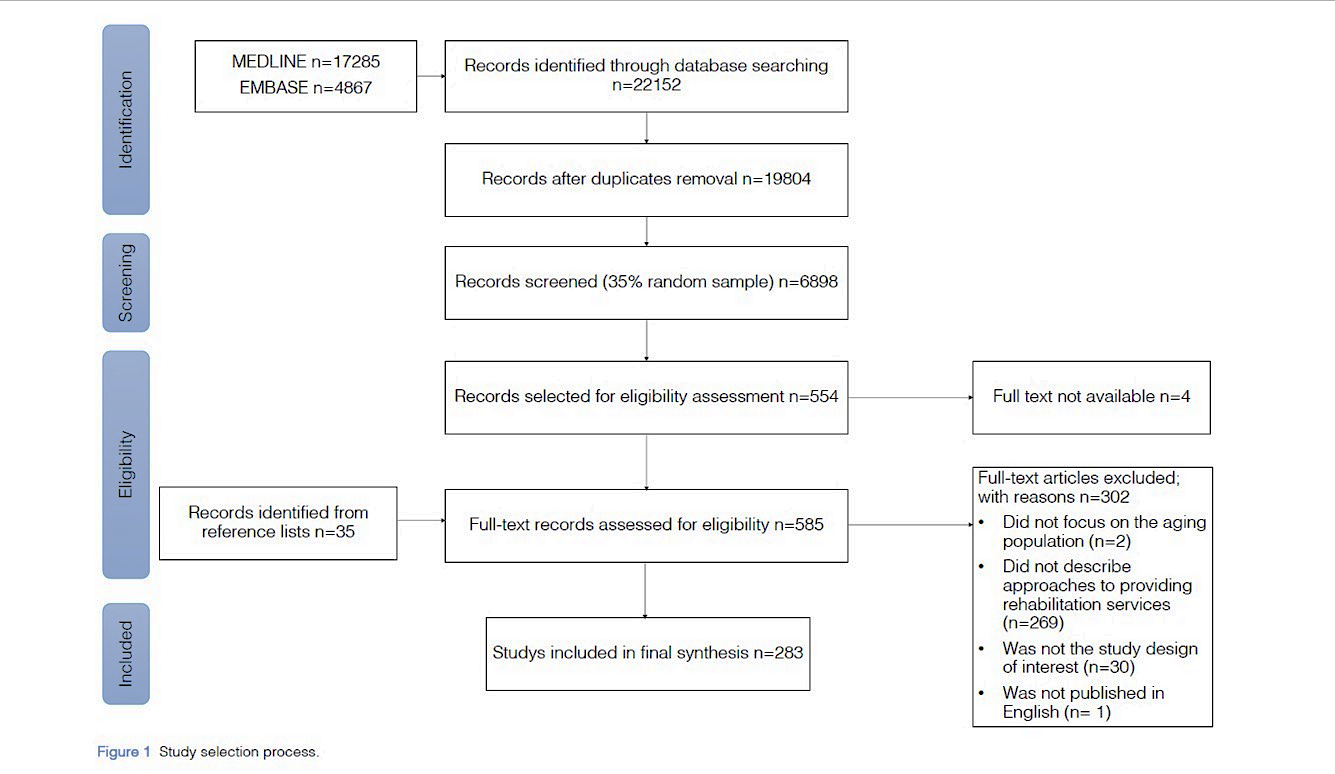
Figure 2: Study selection process of the primary analysis

1. **Definitions**

**Ageing population**

*“It is the shift in the distribution of a country’s population toward older ages. An increase in the population’s mean or median age, a decline in the fraction of the population composed of children, or a rise in the fraction of the population that is elderly*” (World Health Organization, 2015, p. 230)

**Community-based care / community-based services / programs**

*“The blend of health and social services provided to an individual or family in his/her place of residence for the purpose of promoting, maintaining or restoring health or minimizing the effects of illness and disability. These services are usually designed to help older people remain independent and in their own homes. They can include senior centres, transportation, delivered meals or congregate meals sites, visiting nurses or home health aides, adult day care and homemaker services.”* (Andrews, 2004, p. 15)

**Community care**

*“Services and support to help people with care needs to live as independently as possible in their communities.”* (Andrews, 2004, p. 15)

**Nursing**

*“Nursing encompasses autonomous and collaborative care of individuals of all ages, families, groups and communities, sick or well and in all settings. Nursing includes the promotion of health, prevention of illness, and the care of ill, disabled and dying people. Advocacy, promotion of a safe environment, research, participation in shaping health policy and in patient and health systems management, and education are also key nursing roles.”* (International Council of Nurses, 2002)

**Nurse**
*“The nurse is a person who has completed a program of basic, generalized nursing education and is authorized by the appropriate regulatory authority to practice nursing in his/her country. Basic nursing education is a formally recognised programme of study providing a broad and sound foundation in the behavioural, life, and nursing sciences for the general practice of nursing, for a leadership role, and for post-basic education for specialty or advanced nursing practice. The nurse is prepared and authorized (1) to engage in the general scope of nursing practice, including the promotion of health, prevention of illness, and care of physically ill, mentally ill, and disabled people of all ages and in all health care and other community settings; (2) to carry out health care teaching; (3) to participate fully as a member of the health care team; (4) to supervise and train nursing and health care auxiliaries; and (5) to be involved in research.”* (International Council of Nurses, 1987)

**Primary care**

*“It is a model of care that supports first-contact, accessible, continuous, comprehensive and coordinated person-focused care. It aims to optimize population health and reduce disparities across the population by ensuring that subgroups have equal access to services. There are five core functions of primary care: (1) First contact accessibility creates a strategic entry point for and improves access to health services. (2) Continuity promotes the development of long-term personal relationships between a person and a health professional or a team of providers. (3) Comprehensiveness ensures that a diverse range of promotive, protective, preventive, curative, rehabilitative, and palliative services are provided. (4) Coordination organizes services and care across levels of the health system and over time. People-centred care ensures that people have the education and support needed to make decisions and participate in their own care.”* (World Health Organization, 2023a)

**Primary health care (PHC)**

*“Primary health care is a whole-of-society approach to effectively organize and strengthen national health systems to bring services for health and wellbeing closer to communities. It has 3 components: (1) integrated health services to meet people’s health needs throughout their lives (2) addressing the broader determinants of health through multisectoral policy and action (3) empowering individuals, families and communities to take charge of their own health. Primary health care enables health systems to support a person’s health needs – from health promotion to disease prevention, treatment, rehabilitation, palliative care and more. This strategy also ensures that health care is delivered in a way that is centred on people’s needs and respects their preferences. Primary health care is widely regarded as the most inclusive, equitable and cost-effective way to achieve universal health coverage. It is also key to strengthening the resilience of health systems to prepare for, respond to and recover from shocks and crises.”* (World Health Organization, 2023b) The authors in the primary analysis categorized the level of care as PHC when articles self-identified as PHC, when rehabilitation interventions were provided solely by PHC workers, including nurses or general practitioners in a traditional PHC setting (home or community) or when interventions provided did not require complex equipment or specialized training. Studies in which both settings were observed, for example, services starting at a university hospital but continuing with a community exercise program, were classified as *“Multiple levels of care”.*

**Rehabilitation interventions**To achieve a standard categorization of rehabilitation interventions, the authors of the primary analysis used the International Classification of Health Interventions (ICHI) (World Health Organization, 2022a) and the WHO packages of Interventions for rehabilitation (PIR) included in the Universal Health Coverage Compendium (UHC) (*UHC Compendium*, 2023). The term *“rehabilitation intervention”* corresponds to the “action” level in the ICHI and UHC taxonomies. Based on these frameworks, the authors defined six rehabilitation intervention categories: (1) assessment, (2) pharmacological agents (PA), (3) restorative and compensatory approaches, (4) provision of assistive technologies (AT), (5) environmental adaptations (EA), and (6) education and advice. The International Classification of Functioning Disability and Health (ICF)’ linking rules (World Health Organization, 2001) were used to categorize study outcomes.

**Models of care**

To describe the rehabilitation service delivery models, the authors of the primary analysis used the International Classification of Service Organization in Rehabilitation (ICSO-R 2.0) (Gutenbrunner et al., 2020), the Effective Practice and Organisation of Care taxonomy of Health Systems Interventions (EPOC Taxonomy, n.d.) and WHO’s definition of *“model of care”* – *“defined as an evolving conception of how services should be delivered. The evolution of the model of care implies changes to services delivery processes in response, including in the design of care, organization of providers, management of services and continuous performance improvement”* (World Health Organization, 2019)*.*
