# Supplementary material for: The Role of Nurses in Rehabilitation in Primary Health Care for Ageing Populations: A Secondary Analysis from a Scoping Review
Source: SAGE Open Nurs. 2024 Sep 23;10:23779608241271677. doi: 10.1177/23779608241271677 (PMC11425760; doi:10.1177/23779608241271677)
Supplement: sj-docx-7-son-10.1177_23779608241271677 - Supplemental material for The Role of Nurses in Rehabilitation in Primary Health Care for Ageing Populations: A Secondary Analysis from a Scoping Review [file sj-docx-7-son-10.1177_23779608241271677.docx]

# Appendix A Supplementary file 4

## Search concepts, terms, and search strategies for each bibliographic database queried.

| Table S1. Summary of search concepts and terms | | |
| --- | --- | --- |
| Concepts | **Natural language** | **Medical subject headings** |
| Rehabilitation | functioning, convalescence, Telerehabilitation, restorative, rehabi*, reablement, re–ablement, enablement, "speech therapy", "self management" , “self-management", "self care" , "self-care", "rehabilitation medicine", recover*, "recovery of function", "physical therapy", "physical function", "physical exercise", "patient* recovery", "occupational therapy", "occupational functioning", "language therapy", "intrinsic capacit*", "instrumental ADLs", "instrumental activities of daily living", "healthy aging", "functional status", "Functional recovery", "functional health", "functional gain", "functional autonomy", "functional abilit*", "cognitive function*", "activities of daily living", "active ageing" | Convalescence, Rehabilitation Research, Rehabilitation Centers, Telerehabilitation, Speech Therapy, Self-Management, Self Efficacy, Self Care, Rehabilitation, Recovery of Function, Physical Therapy Modalities, Physical and Rehabilitation Medicine, Occupational Therapy, Language Therapy, International Classification of Functioning, Disability and Health, Independent Living, Healthy Aging, Activities of Daily Living. |
| Ageing population | frail, fragility, geria*, retir*, seni*, "old age", "physical decline*", "cognitive decline*", "community-dwelling older adults", "function* impairment*", "Functioning decline", ageing, aging, elder*, "older patients", "older adult*", "older person*", "older people" | Aged, 80 and over, Aged, Middle Aged, Frail Elderly, Geriatrics, Age Factors, Sarcopenia |
| Services | "health planning", "health program", "health service*", "patient care planning", "Community-based", "Community health service", telemonitoring, "transitional care", telehealth, Telerehabilitation, "Long-Term Care", "care model", "team approach", Telecare | Treatment Outcome, Telerehabilitation, Telemedicine, Senior Centers, Program Evaluation, Primary Health Care, Patient Care Team, Patient Care Management, Models, Organizational, Long-Term Care, Home Care Services, Home Care Services, Hospital-Based, Health Services for the Aged, Geriatric Assessment, Delivery of Health Care, Integrated, Comprehensive Health Care, Community Health Nursing, Transitional Care, Quality of Health Care, Program Development, Patient-Centered Care, Outpatient Clinics, Hospital, National Health Programs, Health Services, Health Services Needs and Demand, Health Planning, Community Health Services |

| Table S2. Academic Search Medline search strategy | | |
| --- | --- | --- |
| # | **Query** | **Results** |
| S124 | S43 AND S69 AND S122 + limiters | 17,285 |
| S123 | S43 AND S69 AND S122 | 47,440 |
| S122 | (S70 OR S71 OR S72 OR S73 OR S74 OR S75 OR S76 OR S77 OR S78 OR S79 OR S80 OR S81 OR S82 OR S83 OR S84 OR S85 OR S86 OR S87 OR S88 OR S89 OR S90 OR S91 OR S92 OR S93 OR S94 OR S95 OR S96 OR S97 OR S98 OR S99 OR S100 OR S101 OR S102 OR S103 OR S104 OR S105 OR S106 OR S107 OR S108 OR S109 OR S110 OR S111 OR S112 OR S113 OR S114 OR S115 OR S116 OR S117 OR S118 OR S119 OR S120 OR S121) | 1,459,100 |
| S121 | TI functioning OR AB functioning | 203,555 |
| S120 | TI convalescence OR AB convalescence | 5,210 |
| S119 | TI Telerehabilitation OR AB Telerehabilitation | 1,136 |
| S118 | TI restorative OR AB restorative | 23,889 |
| S117 | TI rehabi* OR AB rehabi* | 187,961 |
| S116 | TI reablement OR AB reablement | 122 |
| S115 | TI re–ablement OR AB re–ablement | 19 |
| S114 | TI enablement OR AB enablement | 649 |
| S113 | TI "speech therapy" OR AB "speech therapy" | 2,659 |
| S112 | TI "self management" OR AB "self management" OR TI "self-management" OR AB "self-management" | 22,395 |
| S111 | TI "self care" OR AB "self care" OR TI "self-care" OR AB "self-care" | 21,067 |
| S110 | TI "rehabilitation medicine" OR AB "rehabilitation medicine" | 1,936 |
| S109 | TI recover* OR AB recover* | 742,299 |
| S108 | TI "recovery of function" OR AB "recovery of function" | 2,500 |
| S107 | TI "physical therapy" OR AB "physical therapy" | 20,119 |
| S106 | TI "physical function" OR AB "physical function" | 16,488 |
| S105 | TI "physical exercise" OR AB "physical exercise" | 17,758 |
| S104 | TI "patient* recovery" OR AB "patient* recovery" | 3,652 |
| S103 | TI "occupational therapy" OR AB "occupational therapy" | 10,884 |
| S102 | TI "occupational functioning" OR AB "occupational functioning" | 1,159 |
| S101 | TI "language therapy" OR AB "language therapy" | 1,396 |
| S100 | TI "intrinsic capacit*" OR AB "intrinsic capacit*" | 865 |
| S99 | TI "instrumental ADLs" OR AB "instrumental ADLs" | 203 |
| S98 | TI "instrumental activities of daily living" OR AB "instrumental activities of daily living" | 4,595 |
| S97 | TI "healthy aging" OR AB "healthy aging" | 5,560 |
| S96 | TI "functional status" OR AB "functional status" | 27,844 |
| S95 | TI "Functional recovery" OR AB "Functional recovery" | 23,888 |
| S94 | TI "functional health" OR AB "functional health" | 2,277 |
| S93 | TI "functional gain" OR AB "functional gain" | 621 |
| S92 | TI "functional autonomy" OR AB "functional autonomy" | 488 |
| S91 | TI "functional abilit*" OR AB "functional abilit*" | 7,554 |
| S90 | TI "cognitive function*" OR AB "cognitive function*" | 72,253 |
| S89 | TI "activities of daily living" OR AB "activities of daily living" | 30,175 |
| S88 | TI "active ageing" OR AB "active ageing" | 290 |
| S87 | (MH "Convalescence") | 3,899 |
| S86 | (MH "Rehabilitation Research") | 232 |
| S85 | (MH "Rehabilitation Centers") | 8,532 |
| S84 | (MH "Telerehabilitation") | 754 |
| S83 | (MH "Speech Therapy") | 6,701 |
| S82 | (MH "Self-Management") | 4,424 |
| S81 | (MH "Self Efficacy") | 23,098 |
| S80 | (MH "Self Care") | 35,221 |
| S79 | (MH "Rehabilitation") | 18,647 |
| S78 | (MH "Recovery of Function") | 58,180 |
| S77 | (MH "Physical Therapy Modalities") | 39,346 |
| S76 | (MH "Physical and Rehabilitation Medicine") | 3,486 |
| S75 | (MH "Occupational Therapy") | 14,210 |
| S74 | (MH "Language Therapy") | 2,040 |
| S73 | (MH "International Classification of Functioning, Disability and Health") | 775 |
| S72 | (MH "Independent Living") | 9,537 |
| S71 | (MH "Healthy Aging") | 1,721 |
| S70 | (MH "Activities of Daily Living") | 70,033 |
| S69 | S44 OR S45 OR S46 OR S47 OR S48 OR S49 OR S50 OR S51 OR S52 OR S53 OR S54 OR S55 OR S56 OR S57 OR S58 OR S59 OR S60 OR S61 OR S62 OR S63 OR S64 OR S65 OR S66 OR S67 OR S68 | 5,821,140 |
| S68 | TI frail OR AB frail | 14,773 |
| S67 | TI fragility OR AB fragility | 18,027 |
| S66 | TI geria* OR AB geria* | 55,508 |
| S65 | TI retir* OR AB retir* | 24,285 |
| S64 | TI seni* OR AB seni* | 65,198 |
| S63 | TI "old age" OR AB "old age" | 27,502 |
| S62 | TI "physical decline*" OR AB "physical decline*" | 441 |
| S61 | TI "cognitive decline*" OR AB "cognitive decline*" | 27,657 |
| S60 | TI "community-dwelling older adults" OR AB "community-dwelling older adults" | 7,180 |
| S59 | TI "function* impairment*" OR AB "function* impairment*" | 25,251 |
| S58 | TI "Functioning decline" OR AB "Functioning decline" | 44 |
| S57 | TI ageing OR AB ageing | 47,107 |
| S56 | TI aging OR AB aging | 203,996 |
| S55 | TI elder* OR AB elder* | 282,288 |
| S54 | TI "older patients" OR AB "older patients" | 44,253 |
| S53 | TI "older adult*" OR AB "older adult*" | 99,963 |
| S52 | TI "older person*" OR AB "older person*" | 12,707 |
| S51 | TI "older people" OR AB "older people" | 33,134 |
| S50 | (MH "Aged, 80 and over") | 1,003,407 |
| S49 | (MH "Aged") | 3,339,004 |
| S48 | (MH "Middle Aged") | 4,669,969 |
| S47 | (MH "Frail Elderly") | 13,772 |
| S46 | (MM "Geriatrics") | 27,118 |
| S45 | (MM "Age Factors") | 6,297 |
| S44 | (MH "Sarcopenia") | 7,326 |
| S43 | S1 OR S2 OR S3 OR S4 OR S5 OR S6 OR S7 OR S8 OR S9 OR S10 OR S11 OR S12 OR S13 OR S14 OR S15 OR S16 OR S17 OR S18 OR S19 OR S20 OR S21 OR S22 OR S23 OR S24 OR S25 OR S26 OR S27 OR S28 OR S29 OR S30 OR S31 OR S32 OR S33 OR S34 OR S35 OR S36 OR S37 OR S38 OR S39 OR S40 OR S41 OR S42 | 833,703 |
| S42 | TI "health planning" OR AB "health planning" | 3,600 |
| S41 | TI "health program" OR AB "health program" | 5,859 |
| S40 | TI "health service*" OR AB "health service*" | 120,965 |
| S39 | TI "patient care planning" OR AB "patient care planning" | 114 |
| S38 | TI "Community-based" OR AB "Community-based" | 68,095 |
| S37 | TI "Community health service" OR AB "Community health service" | 503 |
| S36 | TI telemonitoring OR AB telemonitoring | 1,816 |
| S35 | TI "transitional care" OR AB "transitional care" | 1,668 |
| S34 | TI telehealth OR AB telehealth | 7,883 |
| S33 | TI Telerehabilitation OR AB Telerehabilitation | 1,136 |
| S32 | TI "Long-Term Care" OR AB "Long-Term Care" | 22,967 |
| S31 | TI program | 165,422 |
| S30 | TI "care model" OR AB "care model" | 6,323 |
| S29 | TI "team approach" OR AB "team approach" | 6,898 |
| S28 | TI Telecare OR AB Telecare | 710 |
| S27 | (MM "Treatment Outcome") | 8,065 |
| S26 | (MH "Telerehabilitation") | 754 |
| S25 | (MH "Telemedicine") | 33,078 |
| S24 | (MH "Senior Centers") | 104 |
| S23 | (MM "Program Evaluation") | 11,451 |
| S22 | (MM "Primary Health Care") | 54,906 |
| S21 | (MM "Patient Care Team") | 28,993 |
| S20 | (MM "Patient Care Management") | 3,018 |
| S19 | (MM "Models, Organizational") | 6,482 |
| S18 | (MH "Long-Term Care") | 27,588 |
| S17 | (MH "Home Care Services") | 35,507 |
| S16 | (MM "Home Care Services, Hospital-Based") | 1,579 |
| S15 | (MH "Health Services for the Aged") | 18,137 |
| S14 | (MH "Geriatric Assessment") | 31,005 |
| S13 | (MH "Delivery of Health Care, Integrated") | 13,902 |
| S12 | (MH "Comprehensive Health Care") | 6,747 |
| S11 | (MH "Community Health Nursing") | 19,737 |
| S10 | (MH "Transitional Care") | 1,111 |
| S9 | (MH "Quality of Health Care") | 76,490 |
| S8 | (MH "Program Development") | 30,183 |
| S7 | (MH "Patient-Centered Care") | 21,980 |
| S6 | (MH "Outpatient Clinics, Hospital") | 15,835 |
| S5 | (MH "National Health Programs") | 33,146 |
| S4 | (MH "Health Services") | 26,636 |
| S3 | (MH "Health Services Needs and Demand") | 54,889 |
| S2 | (MH "Health Planning") | 21,898 |
| S1 | (MH "Community Health Services") | 32,760 |
| Limiters: Date of Publication: 20150101-20220531; English Language; Human | | |

| Table S3. Academic Search Medline search strategy | | |
| --- | --- | --- |
| # | **Query** | **Results** |
| #11 | #7 AND #8 AND #9 + limiters | 4,867 |
| #10 | #7 AND #8 AND #9 | 12,197 |
| #9 | #3 OR #6 | 1,626,662 |
| #8 | #2 OR #5 | 898,194 |
| #7 | #1 OR #4 | 917,320 |
| #6 | functioning:ti,kw OR convalescence:ti,kw OR telerehabilitation:ab,ti,kw OR restorative:ti,kw OR rehabi*:ab,ti,kw OR reablement:ab,ti,kw OR re–ablement:ab,ti,kw OR enablement:ab,ti,kw OR 'speech therapy':ab,ti,kw OR 'self management':ti,kw OR 'self-management':ti,kw OR 'self care':ti,kw OR 'self-care':ti,kw OR 'rehabilitation medicine':ab,ti,kw OR recover*:ab,ti,kw OR 'recovery of function':ab,ti,kw OR 'physical therapy':ab,ti,kw OR 'physical function':ti,kw OR 'physical exercise':ti,kw OR 'patient*recovery':ti,kw OR 'occupational therapy':ab,ti,kw OR 'occupational functioning':ab,ti,kw OR 'language therapy':ab,ti,kw OR 'intrinsic capacit*':ab,ti,kw OR 'instrumental adls':ab,ti,kw OR 'instrumental activities of daily living':ab,ti,kw OR 'healthy aging':ab,ti,kw OR 'functional status':ti,kw OR 'functional recovery':ab,ti,kw OR 'functional health':ab,ti,kw OR 'functional gain':ab,ti,kw OR 'functional autonomy':ab,ti,kw OR 'functional abilit*':ab,ti,kw OR 'cognitive function*':ti,kw OR 'activities of dailyliving':ab,ti,kw OR 'active ageing':ab,ti,kw | 1,469,268 |
| #5 | frail:ab,ti,kw OR fragility:ab,ti,kw OR geria*:ab,ti,kw OR retir*:ti,kw OR seni*:ab,ti,kw OR 'old age':ti,kw OR 'physical decline*':ab,ti,kw OR 'cognitive decline*':ab,ti,kw OR 'community-dwelling older adults':ab,ti,kw OR 'function* impairment*':ti,kw OR 'functioning decline':ti,kw OR ageing:ti,kw OR aging:ti,kw OR elder*:ab,ti,kw OR 'older patients':ti,kw OR 'older adult*':ti,kw OR 'older person*':ti,kw OR 'older people':ti,kw | 860,667 |
| #4 | 'health program':ab,ti,kw OR 'health service*':ab,ti,kw OR 'patient care planning':ab,ti,kw OR 'community-based':ab,ti,kw OR 'community health service':ab,ti,kw OR telemonitoring:ab,ti,kw OR 'transional care':ab,ti,kw OR telehealth:ab,ti,kw OR telerehabilitation:ab,ti,kw OR 'long-term care':ab,ti,kw OR program:ti OR 'care model':ab,ti,kw OR 'team approach':ab,ti,kw OR telecare:ab,ti,kw | 479,293 |
| #3 | 'convalescence'/mj OR 'rehabilitation research'/exp OR 'rehabilitation center'/exp OR 'telerehabilitation'/exp OR 'speech therapy'/exp OR 'self care'/mj OR 'rehabilitation'/exp/mj OR 'physiotherapy'/exp OR 'rehabilitation medicine'/exp OR 'occupational therapy'/exp OR 'language therapy'/exp OR 'international classification of functioning, disability and health'/exp OR 'independent living'/exp OR 'healthy aging'/exp OR 'daily life activity'/mj | 329,501 |
| #2 | 'very elderly'/exp/mj OR 'aged'/exp/mj OR 'middle aged'/exp/mj OR 'geriatrics'/exp/mj OR 'sarcopenia'/exp/mj | 108,048 |
| #1 | treatment outcome'/mj OR 'telerehabilitation'/mj OR 'telemedicine'/mj OR 'senior center'/mj OR 'program evaluation'/mj OR 'primary health care'/mj OR 'patient care'/mj OR 'long term care'/mj OR 'home care'/mj OR 'elderly care'/exp/mj OR 'geriatric assessment'/mj OR 'integrated health care system'/mj OR 'health care'/mj OR 'community health nursing'/mj OR 'transitional care'/mj OR 'health care quality'/mj OR 'program development'/exp/mj OR 'outpatient department'/mj OR 'health service'/mj OR 'health care planning'/mj OR 'community care'/mj | 507,343 |
| Limiters: Date of Publication: 20150101-20220531; English Language; Human | | |
