# Supplementary material for: The Role of Nurses in Rehabilitation in Primary Health Care for Ageing Populations: A Secondary Analysis from a Scoping Review
Source: SAGE Open Nurs. 2024 Sep 23;10:23779608241271677. doi: 10.1177/23779608241271677 (PMC11425760; doi:10.1177/23779608241271677)
Supplement: sj-docx-8-son-10.1177_23779608241271677 - Supplemental material for The Role of Nurses in Rehabilitation in Primary Health Care for Ageing Populations: A Secondary Analysis from a Scoping Review [file sj-docx-8-son-10.1177_23779608241271677.docx]

***Appendix A Supplementary file 5***

**Data extraction form**

| # | Name of the variable | Description/instructions |
| --- | --- | --- |
| Characteristics of the studies | | |
|  | Key | According to the “Rayyan Systematic Review Screening Software” |
|  | PMID | Enter PMID number if available |
|  | Title | Copy, paste and enter the title of the study |
|  | Publication year | Enter publication year of the study |
|  | Journal | Enter journal of the study |
|  | Authors | Enter all authors of the study |
|  | Abstract | Enter copied abstract of the study |
|  | Country | Add the country or countries in which the study took place. Use the country’s English name, do not use abbreviations, and use capital letters. |
|  | Study design | Enter the studies’ design as described |
|  | Study design (category) | Select the most appropriate design   - Intervention study - Observational study - Qualitative study |
| Characteristics of the target population | | |
|  | Target population | Copy and paste information about the target population |
|  | Target population (category) | Select the most appropriate one:   - Single health condition - Multimorbidity - Fragility or functional |
|  | Health condition | Write the health conditions’ name. If patients with several single health conditions were included, add them separated by a comma without space. For example: diabetes,stroke,hip fracture. If the target population (transformed) was "Multimorbidity," just add multimorbidity.  If it was fragility or patients with functional decline, add: N/A |
|  | Health condition area | Select the most appropriate:   - Neurological - Cardiovascular - Musculoskeletal - Respiratory - Cancer - Metabolic - Communicable diseases - Pain - Autoimmune disorders - Sensory - Urological - Not reported |
|  | Age-related inclusion (transformed) | Select the most appropriate:   - Older than 18 - Older than 50 - Older than 60 - Older than 65 - Older than 70 - Older than 75 - Ceiling age at 70 - Ceiling age at 75 - Ceiling age at 80 - Ceiling age at 85 - Ceiling age at 90 |
|  | Mean age (transformed) | Report the participant's mean age, up to two decimal points if the mean age was reported only by groups, sum, and divide by the number of groups. For example, if two groups were included, sum the two mean ages and divide them by two.  If necessary: add Not reported. |
|  | Number of participants | Enter the total number of participants, including intervention and control group.  If necessary: add Not reported |
|  | Gender (% male) | Copy and Paste from the studies |
|  | Participants' sex predominance (transformed) | Select the most appropriate:   - If more than 60% of study participants were males: Male predominance - If less than 40% of study participants were males: Female predominance - If males represent between 40-60%: Similar   If necessary: add Not reported |
| Characteristics of the rehabilitation interventions in PHC | | |
|  | Description of the intervention | Copy and paste the description from the methods section. |
|  | Intervention summary | Enter a summary of the most important aspects of the intervention, model, or strategy. |
|  | Intervention type | Select the most appropriate according to the EPOC taxonomy + Universal Health compendium + Researchers |
|  | Rural or urban?* | Describe whether the intervention took place in the urban or rural area. Enter only the words that have been used in the studies.  If necessary, add: Not reported |
|  | Co-design of intervention* | Describe openly whether patients and/or caregivers were involved in the design of the intervention. This absolutely must take place BEFORE the intervention and is about determining if their opinion, preferences, etc. have been considered and included in the intervention. |
|  | How was the intensity of rehabilitation decided?* | Enter the most appropriate one.  Prespecified program= if no adaptations were made  Adapted considering patients' needs= if the patient/provider could freely choose which intervention was provided  Prespecified program,adapted considering patients' needs= if the intensity of the intervention was adapted to the patients' resources and abilities  If necessary: add Not reported |
|  | Name of intervention* | Copy and paste from the study’s description (intervention section). |
|  | Single or multiple types of sessions?* | Report if the interventions provided single or multiple sessions. |
| AB. | Session 1* | If "single session", only briefly describe the content in "session 1". If mutliple sessions took place, briefly describe the several components provided in the intervention in "session 2 to 8". If no information is available indicate "Not reported" and if no session has been provided indicate "Not applicable". |
| AC. | Session 2* | If "single session", only briefly describe the content in "session 1". If mutliple sessions took place, briefly describe the several components provided in the intervention in "session 2 to 8". If no information is available indicate "Not reported" and if no session has been provided indicate "Not applicable". |
| AD. | Session 3* | If "single session", only briefly describe the content in "session 1". If mutliple sessions took place, briefly describe the several components provided in the intervention in "session 2 to 8". If no information is available indicate "Not reported" and if no session has been provided indicate "Not applicable". |
| AE. | Session 4* | If "single session", only briefly describe the content in "session 1". If mutliple sessions took place, briefly describe the several components provided in the intervention in "session 2 to 8". If no information is available indicate "Not reported" and if no session has been provided indicate "Not applicable". |
| AF. | Session 5* | If "single session", only briefly describe the content in "session 1". If mutliple sessions took place, briefly describe the several components provided in the intervention in "session 2 to 8". If no information is available indicate "Not reported" and if no session has been provided indicate "Not applicable". |
| AG. | Session 6* | If "single session", only briefly describe the content in "session 1". If mutliple sessions took place, briefly describe the several components provided in the intervention in "session 2 to 8". If no information is available indicate "Not reported" and if no session has been provided indicate "Not applicable". |
| AH. | Session 7* | If "single session", only briefly describe the content in "session 1". If mutliple sessions took place, briefly describe the several components provided in the intervention in "session 2 to 8". If no information is available indicate "Not reported" and if no session has been provided indicate "Not applicable". |
| AI. | Session 8* | If "single session", only briefly describe the content in "session 1". If mutliple sessions took place, briefly describe the several components provided in the intervention in "session 2 to 8". If no information is available indicate "Not reported" and if no session has been provided indicate "Not applicable". |
| AJ. | Time session 1 (in min)* | Report the time for each session. Write the number in minutes or calculate it. |
| AK. | Time session 2 (in min)* | Report the time for each session. Write the number in minutes or calculate it. |
| AL. | Time session 3 (in min)* | Report the time for each session. Write the number in minutes or calculate it. |
| AM. | Time session 4 (in min)* | Report the time for each session. Write the number in minutes or calculate it. |
| AN. | Time session 5 (in min)* | Report the time for each session. Write the number in minutes or calculate it. |
| AO. | Time session 6 (in min)* | Report the time for each session. Write the number in minutes or calculate it. |
| AP. | Time session 7 (in min)* | Report the time for each session. Write the number in minutes or calculate it. |
| AQ. | Time session 8 (in min)* | Report the time for each session. Write the number in minutes or calculate it. |
| AR. | Time of the longest session (in minutes)* | Report only the time in minutes per session that occurred the for the longest time. For example, if psychotherapy lasted 40 minutes and physiotherapy 30 minutes, write 40. |
| AS. | Average of sessions' time (in minutes)* | Sum of all individual session’s duration in minutes. For example, Nurse visits 10 minutes, guided physiotherapy 30 minutes, occupational therapy 15 minutes. Add here 18.33. |
| AT. | Frequency session 1 (per week)* | Report the frequency of the session per week. Write the number (1, 2 or 3 sessions per week) or select= Not reported. Hand calculations are also possible. For example, the paper reported "1 session every two weeks", divide the number of sessions, by the number of weeks, (1/2 = 0.5), 1 session every month, 1/4,=0.25. Other calculations are also possible, for example, 1 session every 10 weeks, write 1/10=0.1. Use 4 to calculate the number of weeks for simplicity. |
| AU. | Frequency session 2 (per week)* | Report the frequency of the session per week. Write the number (1, 2 or 3 sessions per week) or select= Not reported. Hand calculations are also possible. For example, the paper reported "1 session every two weeks", divide the number of sessions, by the number of weeks, (1/2 = 0.5), 1 session every month, 1/4,=0.25. Other calculations are also possible, for example, 1 session every 10 weeks, write 1/10=0.1. Use 4 to calculate the number of weeks for simplicity. |
| AV. | Frequency session 3 (per week)* | Report the frequency of the session per week. Write the number (1, 2 or 3 sessions per week) or select= Not reported. Hand calculations are also possible. For example, the paper reported "1 session every two weeks", divide the number of sessions, by the number of weeks, (1/2 = 0.5), 1 session every month, 1/4,=0.25. Other calculations are also possible, for example, 1 session every 10 weeks, write 1/10=0.1. Use 4 to calculate the number of weeks for simplicity. |
| AW. | Frequency session 4 (per week)* | Report the frequency of the session per week. Write the number (1, 2 or 3 sessions per week) or select= Not reported. Hand calculations are also possible. For example, the paper reported "1 session every two weeks", divide the number of sessions, by the number of weeks, (1/2 = 0.5), 1 session every month, 1/4,=0.25. Other calculations are also possible, for example, 1 session every 10 weeks, write 1/10=0.1. Use 4 to calculate the number of weeks for simplicity. |
| AX. | Frequency session 5 (per week)* | Report the frequency of the session per week. Write the number (1, 2 or 3 sessions per week) or select= Not reported. Hand calculations are also possible. For example, the paper reported "1 session every two weeks", divide the number of sessions, by the number of weeks, (1/2 = 0.5), 1 session every month, 1/4,=0.25. Other calculations are also possible, for example, 1 session every 10 weeks, write 1/10=0.1. Use 4 to calculate the number of weeks for simplicity. |
| AY. | Frequency session 6 (per week)* | Report the frequency of the session per week. Write the number (1, 2 or 3 sessions per week) or select= Not reported. Hand calculations are also possible. For example, the paper reported "1 session every two weeks", divide the number of sessions, by the number of weeks, (1/2 = 0.5), 1 session every month, 1/4,=0.25. Other calculations are also possible, for example, 1 session every 10 weeks, write 1/10=0.1. Use 4 to calculate the number of weeks for simplicity. |
| AZ. | Frequency session 7 (per week)* | Report the frequency of the session per week. Write the number (1, 2 or 3 sessions per week) or select= Not reported. Hand calculations are also possible. For example, the paper reported "1 session every two weeks", divide the number of sessions, by the number of weeks, (1/2 = 0.5), 1 session every month, 1/4,=0.25. Other calculations are also possible, for example, 1 session every 10 weeks, write 1/10=0.1. Use 4 to calculate the number of weeks for simplicity. |
| BA. | Frequency session 8 (per week)* | Report the frequency of the session per week. Write the number (1, 2 or 3 sessions per week) or select= Not reported. Hand calculations are also possible. For example, the paper reported "1 session every two weeks", divide the number of sessions, by the number of weeks, (1/2 = 0.5), 1 session every month, 1/4,=0.25. Other calculations are also possible, for example, 1 session every 10 weeks, write 1/10=0.1. Use 4 to calculate the number of weeks for simplicity. |
| BB. | Average of sessions' frequency* | Sum all the sessions frequencies and divide by the number of sessions for only the frequencies that were reported. |
| BC. | Frequency of the most frequent session (per week)* | Report only the frequency per week that occurred most frequently. Write the number (1, 2 or 3 sessions per week) or select= Not reported. Hand calculations are also possible. For example, the paper reported "1 session every two weeks", divide the number of sessions, by the number of weeks, (1/2 = 0.5), 1 session every month, 1/4,=0.25. Other calculations are also possible, for example, 1 session every 10 weeks, write 1/10=0.1. Use 4 to calculate the number of weeks for simplicity. |
| BD. | Grouped version of all sessions' duration (in minutes) with the frequency (per week)* | Report the sessions name=duration in minutes (frequency per week). Put * to indicate that sessions only took place once. |
| BE. | Total number of sessions that each patient received* | Sum of all individual sessions. For example, 2 Nurse visits, 2 guided physioterapy, 2 occupational therapy. Add here 6. |
| BF. | Total duration of the intervention (in weeks)* | Report the number of weeks the program lasted or select “Not reported” or “Individualized”.  Select “Individualized” when the time was adjusted according to the patient's needs.  If time is provided in months, multiply by 4 to calculate the number of weeks for simplicity. |
| BG. | Comments on time and intensity | Report additional information |
| BH. | Paper’s conclusion | Report what the authors conclude about the intervention |
| BI. | Paper's conclusion (transformed) | Select one of the options:  1. Authors found the intervention effective  2. Authors found the intervention not effective or not different than the usual care  3. Can't be assessed |
| BJ. | Outcomes | If there is more than one outcome, separate them with commas without spaces.  Ex: Independence in activities of daily living (Barthel index),Quality of life (SF-36) |
| BK. | Rehabilitation provider | Write like this:   - Health care workers - Allied health care workers - Peers and volunteers - Informal caregivers and family - The patient - Not reported/NA - If there is more than one provider, separate them by commas with no spaces. |
| BL. | Multidisciplinary rehabilitation team | Enter Yes: if at least 3 providers |
| BM. | Self-management | Report if the studies aimed at supporting self-management.  Yes  No |
| BN. | Health worker rehabilitation provider (filtered)* | Write like this:  Geriatrician  Nurse  Physical therapist  Occupational therapist  Dieticians  General practitioner  etc  If there is more than one provider, separate them by commas with no spaces. |
| BO. | Level of care (filtered)* | Enter the most appropriate one:   - Primary Health care - Multiple levels of care |
| BP. | Mode of service delivery | Enter the most appropriate one:  Outpatient  Inpatient  Home  Eldercare institution  Telerehabilitation  Community  If there is more than one mode of service delivery, separate them by commas with no spaces. |
| BQ. | Aim healthy ageing? | Enter Yes if the paper says that the aim is to contribute to healthy ageing. Enter No if the paper does not provide information on this. |
| BR. | Integrated care? | Enter Yes or No if the paper includes integrated care.  It is also known as integrated health, coordinated care, comprehensive care, seamless care, or transmural care, is a worldwide trend in health care reforms and new organizational arrangements focusing on more coordinated and integrated forms of care provision.  WHO definition: "Integrated care is a concept bringing together inputs, delivery, management and organization of services related to diagnosis, treatment, care, rehabilitation and health promotion. Integration is a means to improve services in relation to access, quality, user satisfaction and efficiency”. |
| BS. | Role or task shifting | Did role or task shifting take place?  Yes  No |
| BT. | Role or task shifting revised* | Did role or task shifting take place where nurses were involved? Describe openly how and which role or task was shifted  If necessary, add: Not reported  Enter Yes if so, No if not  If necessary, add: Not reported |
| Characteristics of the nurses’ role | | |
| BU. | Nurses’ role* | Look at the type of intervention and check which ones were provided by the nurses. If necessary, add: Not reported  If necessary, add: Not reported |
| BV. | Nurses’ interventions* | Describe openly which tasks were delivered by nurses only, e.g. provided the patient with medication, etc.  If necessary, add: Not reported |
| BW. | Nurses’ role (transformed)* | Categorize the nurses’ roles in managerial, clinical, both, etc. |
| BX. | Nurses’ communication* | Describe openly how the nurses communicated with the other health care workers or community workers or with the patients.  For example: weekly meetings with GPs for two hours |
| BY. | Nurses’ title or designation* | Enter how the nurses were designed in the study, as for example:   - Advanced Practice Nurse - Clinical Nurse Specialist - Community Nurse   Nurse not further specified   - Nurse Practitioner   Registered Nurse |
| BZ. | Nurses’ training* | - Describe openly if the nurses were provided with any training to deliver the intervention. - If necessary, add: Not reported |
| CA. | Nurses’ work independence* | Describe openly which work independence nurses had in providing the intervention.  For example: worked under close oversight and direction by the GP, worked autonomously, etc. led the nursing-home |
| CB. | Additional information about the nurses' role* | Describe openly if there were any further important aspects on how the role of the nurses was organized in the intervention. |
| CC. | Notes | Write any notes that would be important to report |
| CD. | Comments (Examples) | Describe any examples from the studies |
