# Supplementary material for: The Role of Nurses in Rehabilitation in Primary Health Care for Ageing Populations: A Secondary Analysis from a Scoping Review
Source: SAGE Open Nurs. 2024 Sep 23;10:23779608241271677. doi: 10.1177/23779608241271677 (PMC11425760; doi:10.1177/23779608241271677)
Supplement: sj-docx-9-son-10.1177_23779608241271677 - Supplemental material for The Role of Nurses in Rehabilitation in Primary Health Care for Ageing Populations: A Secondary Analysis from a Scoping Review [file sj-docx-9-son-10.1177_23779608241271677.docx]

| Levels of care, Modes of services delivery | **Author(s) Year** | **Country** | **Nurses’**  **title(s)** | **Rehabilitation interventions provided by nurses** | **Other health workers** | **Nurse-led interventions** | **Task shifting** | **Comments about nurses’ role** | |  |
| --- | --- | --- | --- | --- | --- | --- | --- | --- | --- | --- |
|  | **Studies with nurses in a managerial and clinical role (N=52)** | | | | | | | |  |  |
| Multiple, Telerehabilitation | (Bekelman et al., 2015) | United States | Nurse  coordinator | Assessment of emotional functions | General  practitioners | No | No | Each site had a collaborative care team consisting of a nurse coordinator (registered nurse) a primary care physician a cardiologist and a psychiatrist. The nurse was supervised by the site psychiatrist via as-needed calls with the lead psychiatrist and Dr Bekelman. The depression care component of the nurse intervention was standardized in a treatment manual that described detailed procedures for what to discuss with patients. | |  |
|  |  |  |  | Education and skills training for self-care and self-management |  |  |  |  |  |  |
|  |  |  |  | Health status monitoring |  |  |  |  |  |  |
|  |  |  |  | Monitoring of functional ability |  |  |  |  |  |  |
|  |  |  |  | Psychological interventions |  |  |  |  |  |  |
|  |  |  |  | Rehabilitation coordination and management not further specified |  |  |  |  |  |  |
| PHC, Outpatient | (Bleijenberg et al., 2016) | Netherlands | Registered practice nurse | Assessment of frailty | General  practitioners | **Yes** | No | Twenty-one registered practice nurses delivered this care and were extensively trained during a 6-week training program (48 hours total). An expert panel of older adults nurses and GPs participated in the development of this program. | |  |
|  |  |  |  | Case management |  |  |  |  |  |  |
|  |  |  |  | Comprehensive geriatric assessment |  |  |  |  |  |  |
|  |  |  |  | Follow up visits |  |  |  |  |  |  |
|  |  |  |  | Management of urinary incontinence |  |  |  |  |  |  |
|  |  |  |  | Monitoring of functional ability |  |  |  |  |  |  |
|  |  |  |  | Multicomponent rehabilitation care or not specified |  |  |  |  |  |  |
| PHC, Home | (Bleijenberg et al., 2017) | Netherlands | Registered nurse (RN) | Assessment of family and caregivers' needs knowledge and skills | None | **Yes** | No | The UPROFIT intervention was delivered by registered nurses and included a frailty assessment and a comprehensive geriatric assessment (CGA) at home followed by an individualized evidence-based care plan care coordination and follow-up. The HCP intervention was delivered by advanced practice nurses consisting of four home visits and three phone calls and was guided by the principles of health promotion empowerment partnership and family-centeredness. | |  |
|  |  | Switzerland | Advanced practice nurse (APN) | Assessment of person-centered goals |  |  |  |  |  |  |
|  |  |  |  | Case management |  |  |  |  |  |  |
|  |  |  |  | Comprehensive geriatric assessment |  |  |  |  |  |  |
|  |  |  | Community health nurse | Education and skills training for caregivers |  |  |  |  |  |  |
|  |  |  |  | Education and skills training for self-care and self-management |  |  |  |  |  |  |
|  |  |  |  | Follow up visits |  |  |  |  |  |  |
|  |  |  |  | Health status monitoring |  |  |  |  |  |  |
|  |  |  |  | Management of urinary incontinence |  |  |  |  |  |  |
|  |  |  |  | Monitoring of functional ability |  |  |  |  |  |  |
|  |  |  |  | Multicomponent rehabilitation care or not specified |  |  |  |  |  |  |
| Multiple, Outpatient | (Blom et al., 2016) | Netherlands | Practice nurse | Assessment of cognitive functions | General  practitioners | No | Yes | In the Netherlands all community-dwelling persons are registered at a GP. The GP or the practice nurse (under supervision of the GP) made an integrated care plan for participants with complex problems. The GP/practice nurse together with the older person formulated actions to be taken and evaluation plans for follow-up. Other care professionals were involved where needed (multidisciplinary consultation). | |  |
|  |  |  |  | Assessment of functioning |  |  |  |  |  |  |
|  |  |  |  | Assessment of person-centered goals |  |  |  |  |  |  |
|  |  |  | Research nurse | Case management |  |  |  |  |  |  |
|  |  |  |  | Follow up visits |  |  |  |  |  |  |
|  |  |  |  | Multicomponent rehabilitation care or not specified |  |  |  |  |  |  |
| Multiple, Inpatient / Outpatient | (Buurman et al., 2016) | Netherlands | Geriatric-trained registered nurse | Assessment of environment | None | **Yes** | Yes | After the geriatric-trained registered nurse conducted the CGA the CCRN was contacted to visit the hospital to receive a personal handover of the CGA to initiate the personalized care and treatment plan (CTP) and to meet with the participant and informal caregiver to discuss their needs. After discharge the CCRN performed medication reconciliation answered the participant’s questions and completed a needs assessment at a home visit. If a participant was discharged to a nursing home the CCRN also visited the nursing home. | |  |
|  |  |  | Community care registered nurse (CCRN) | Assessment of person-centered goals |  |  |  |  |  |  |
|  |  |  | Experienced trained research nurse | Comprehensive geriatric assessment |  |  |  |  |  |  |
|  |  |  | Advanced practice nurse (APN) | Discharge planning |  |  |  |  |  |  |
| PHC, Telerehabilitation | (Cameron-Tucker et al., 2016) | Australia | Community nurse | Assessment of person-centered goals | None | **Yes** | No | Participants were contacted via telephone by specifically trained community nurses who acted as nurse health-mentors to support the home-walking action plan and any other health behavior plans. | |  |
|  |  |  | Respiratory nurse  specialist | Education and counseling about healthy lifestyle behaviors |  |  |  |  |  |  |
|  |  |  |  | Education and skills training for self-care and self-management |  |  |  |  |  |  |
|  |  |  |  | Follow up visits |  |  |  |  |  |  |
|  |  |  |  | Therapeutic exercises |  |  |  |  |  |  |
| PHC, Eldercare / Institution | (Clevenger et al., 2018) | United States | Advanced practice  registered nurse (APRN) | Assessment of person-centered goals | Geriatricians | **Yes** | Yes | First Nurse-led Patient centered Medical Home model (PCMH) in which Advanced practice registered nurses (APRNs) are the first-line clinicians. Physician-APRN partnership varies; some physicians permit APRNs to manage issues influenced by cognitive impairment (e.g. falls medication management) whereas others authorize management only of dementia-related concerns. Unlike in other dementia care programs (where APRNs play adjunctive or case management roles) IMCC APRNs are the first-line clinicians. They collaborate with a neurologist and a Geriatricians but patients do not have physician appointments except when the APRNs order specialist consultations. | |  |
|  |  |  |  | Education and skills training for caregivers | Social  workers |  |  |  |  |  |
|  |  |  | Geriatric nurse | Social care and support plan |  |  |  |  |  |  |
| Multiple, Outpatient /  Inpatient | (Coskun & Duygulu, 2022) | Turkey | Coordinator nurse | Assessment of health status | Dieticians | **Yes** | Yes | The Coordinator nurse took the patient's medical history hospital and clinic promotion providing training guide daily regular patient visits coordinating the healthcare services. The Clinic Nurses executed healthcare services in line with designated healthcare plan competing of the practices in the clinical pathway. | |  |
|  |  |  | Clinic nurse | Assessment of person-centered goals | Other  physicians |  |  |  |  |  |
|  |  |  |  | Case management |  |  |  |  |  |  |
|  |  |  |  | Discharge planning | Physical  therapists |  |  |  |  |  |
|  |  |  |  | Follow up visits |  |  |  |  |  |  |
|  |  |  |  | Multicomponent rehabilitation care or not specified |  |  |  |  |  |  |
| Multiple, Outpatient | (Coventry et al., 2015) | United Kingdom | Practice nurse | Management in the use of medication | Other  physicians | No | No | To better achieve integrated care a 10 minute collaborative meeting (by telephone or in person) between the patient and the psychological wellbeing practitioner and a practice nurse from the patient’s general practice was scheduled. Psychological wellbeing practitioners also worked with the patient and practice nurse to check that patients adhered to antidepressants as prescribed dealt with concerns about side effects and helped to arrange drug reviews with the General practitioners if necessary. | |  |
|  |  |  |  | Rehabilitation coordination and management not further specified | Psychologists |  |  |  |  |  |
| Multiple, Inpatient /  Home | (Deng et al., 2020) | China | Nurse | Case management | General  practitioners | No | No | A nurse in the community setting was designated as a coordinator to ensure the continuity of care delivery. | |  |
|  |  |  | Community nurse | Education and skills training for self-care and self-management |  |  |  |  |  |  |
|  |  |  |  | Follow up visits | Other  physicians |  |  |  |  |  |
|  |  |  |  | Health status monitoring |  |  |  |  |  |  |
|  |  |  |  | Multicomponent rehabilitation care or not specified | PRM  physicians |  |  |  |  |  |
| Multiple, Inpatient / Outpatient | (Ekelund & Eklund, 2015) | Sweden | Nurse | Assessment of frailty | Occupational therapists | No | Yes | The intervention involved collaboration between a nurse with geriatric competence at the ED the hospital wards and a multi-professional team in the community with a case manager as the hub. Together they created a continuum of care for the older people from the ED through the hospital ward to their homes. At the ED a nurse with geriatric competence carried out a frailty screening. When the screening indicated that the older person was frail the nurse also completed a brief basic geriatric needs assessment and need of rehabilitation. | |  |
|  |  |  | Municipal nurse | Assessment of person-centered goals |  |  |  |  |  |  |
|  |  |  |  | Comprehensive geriatric assessment | Physical  therapists |  |  |  |  |  |
|  |  |  |  | Discharge planning |  |  |  |  |  |  |
|  |  |  |  | Follow up visits | PRM  physicians |  |  |  |  |  |
|  |  |  |  | Multicomponent rehabilitation care or not specified | Social  workers |  |  |  |  |  |
|  |  |  |  | Rehabilitation coordination and management not further specified |  |  |  |  |  |  |
| Multiple, Inpatient | (Everink et al., 2017) | Netherlands | Discharge nurse | Assessment of functioning | General  practitioners | **Yes** | Yes | A discharge nurse preceded dismissal from the hospital and gathered information about the patient’s functional prognosis endurability teachability and trainability and the patient’s and informal caregiver’s needs and abilities. The triage is always performed under the responsibility of an elderly care physician from the geriatric rehabilitation facility. If the discharge nurse has doubts about the patient’s eligibility for geriatric rehabilitation the elderly care physician should be consulted. The discharge nurse (in hospital) should always provide oral and written information about geriatric rehabilitation to the patient and the informal caregiver. The nurses in the geriatric rehabilitation facility should arrange home care prior to discharge of the patient. Once the patient is discharged from the geriatric rehabilitation facility the nurse practitioner or district nurse in primary care should act as the patient’s case manager. | |  |
|  |  |  | Nurse  practitioner | Case management | Occupational therapists |  |  |  |  |  |
|  |  |  | District nurse | Discharge planning |  |  |  |  |  |  |
|  |  |  |  | Education and skills training for caregivers | Physical  therapists |  |  |  |  |  |
|  |  |  |  | Multicomponent rehabilitation care or not specified |  |  |  |  |  |  |
| Multiple, Inpatient | (Everink et al., 2018) | Netherlands | Discharge nurse | Assessment of functioning | General  practitioners | **Yes** | Yes |  |  |  |
|  |  |  | Nurse  practitioner | Case management | Occupational therapists |  |  |  |  |  |
|  |  |  | District nurse | Discharge planning |  |  |  |  |  |  |
|  |  |  |  | Education and skills training for caregivers | Physical  therapists |  |  |  |  |  |
|  |  |  |  | Multicomponent rehabilitation care or not specified |  |  |  |  |  |  |
| Multiple, Telerehabilitation / Outpatient | (Finlayson et al., 2018) | Australia | Gerontic nurse | Follow up visits | None | **Yes** | Yes | APGN will visit intervention patients undertake a health assessment and prepare a comprehensive transitional care plan. This process includes discussing the planned discharge plan with patient’s caregivers doctor and ward nurses to individualize the plan. APGN will visit patients daily thereafter until discharge to establish and implement the program monitor progress and modify transitional care plan if required. After discharge home the APGN provides one in-home visit. | |  |
|  |  |  | Liaison nurse | Therapeutic exercises |  |  |  |  |  |  |
|  |  |  | Ward nurse |  |  |  |  |  |  |  |
|  |  |  | Advanced practice gerontic nurse (APGN) |  |  |  |  |  |  |  |
| Multiple, Community | (Franse et al., 2018) | United Kingdom | Geriatric nurse  practitioner | Assessment of health status | Other  physicians | **Yes** | No | Most positive effects of the UHCE approach were found in Rijeka. Possible explanations were a high morale to engage in activities among participants and regular monitoring of the care process by community nurses who had a personal relationship with the participants and acted as care coordinator in this study. Establishment of a trusted relationship is important for improvement of uptake and adherence to care interventions among older persons. | |  |
|  |  |  |  | Assessment of functioning |  |  |  |  |  |  |
|  |  | Greece | Community nurse | Assessment of person-centered goals |  |  |  |  |  |  |
|  |  | Croatia | Nurse | Assessment of emotional functions |  |  |  |  |  |  |
|  |  | Spain |  | Assessment of fall risk |  |  |  |  |  |  |
|  |  | Netherlands |  | Assessment of frailty |  |  |  |  |  |  |
|  |  |  |  | Case management |  |  |  |  |  |  |
|  |  |  |  | Follow up visits |  |  |  |  |  |  |
|  |  |  |  | Therapeutic exercises |  |  |  |  |  |  |
|  |  |  |  | Social care and support plan |  |  |  |  |  |  |
| Multiple, Inpatient /  Outpatient | (He et al., 2018) | China | Nurse | Multicomponent rehabilitation care or not specified | General  practitioners | No | No | The leader team comprised a medical specialist as well as nursing and allied health staff with stroke expertise. | |  |
|  |  |  |  |  | Physical  therapists |  |  |  |  |  |
| Multiple, Home / Community / Outpatient | (Hoedemakers et al., 2022) | Netherlands | Nurse  practitioner | Assessment of functioning | General  practitioners | Yes | No | The CCFE is financed by a bundled payment aiming to stimulate collaboration between professionals. The bundled payment is a fixed amount of money per patient that covers all services provided by the General practitioners (GP) nurse–practitioner and physician assistant regardless of diagnoses medication review by the pharmacist telephone consultation by the Geriatricians non-individual-patient-related activities such as building a community network and overhead. To be included the health insurer does not require a specific diagnosis or the use of a screening tool. They trust that the GP and nurse practitioner. | |  |
|  |  |  | District nurse | Assessment of person-centered goals | Geriatricians |  |  |  |  |  |
|  |  |  |  | Case management | Social  workers |  |  |  |  |  |
|  |  |  |  | Comprehensive geriatric assessment | Physical  therapists |  |  |  |  |  |
|  |  |  |  | Multicomponent rehabilitation care or not specified | Occupational therapists |  |  |  |  |  |
|  |  |  |  | Therapeutic exercises |  |  |  |  |  |  |
| Multiple, Inpatient / Outpatient | (K. Y. F. Wong & Yeung, 2015) | China | Nurse case manager | Assessment of functioning | None | No | No | In this study appointed holistic care managers (HCMs) acted as care providers and coordinators in the process of care. Nurses usually assume the role of a case manager supported by a multidisciplinary team in providing direct nursing interventions and negotiating care with multiple providers (Hammer 2005 Wong et al. 2011). These direct interventions include teaching self-administered treatment techniques counselling on appropriate health behavior reinforcing medication adherence and symptom management (Hammer 2005 Wong et al. 2008 Chow & Wong 2010). The negotiation and coordination of care by the nurse is crucial to ensure that the patients receive the appropriate type and level of care. The nurse case manager is a pivotal person in the programme providing direct interventions and coordinating input from the multidisciplinary team if needed. | |  |
|  |  |  |  | Assessment of environment |  |  |  |  |  |  |
|  |  |  |  | Behavioral interventions |  |  |  |  |  |  |
|  |  |  |  | Case management |  |  |  |  |  |  |
|  |  |  |  | Discharge planning |  |  |  |  |  |  |
|  |  |  |  | Education and counseling about healthy lifestyle behaviors |  |  |  |  |  |  |
|  |  |  |  | Education and skills training for caregivers |  |  |  |  |  |  |
|  |  |  |  | Education and skills training for self-care and self-management |  |  |  |  |  |  |
|  |  |  |  | Follow up visits |  |  |  |  |  |  |
|  |  |  |  | Multicomponent rehabilitation care or not specified |  |  |  |  |  |  |
|  |  |  |  | Therapeutic exercises |  |  |  |  |  |  |
| Multiple, Inpatient / Home | (K. Y. F. Wong et al., 2022) | China | Intervention nurse | Assessment of functioning | Neurologists | **Yes** | No | A nurse case manager played a pivotal role in care delivery and coordination under the multidisciplinary approach. The collaboration efforts were realized by having the MDT participate from the stage of protocol design to taking referrals from the nurse case manager (NCM) in the intervention phase. The NCM delivered and coordinated planned events during the 12-week program to ensure continuity of care. A nurse-managed hotline was available throughout the 12 weeks. The intervention team involved the MDT and a nurse assigned as case manager who had the primary responsibility for conducting home visits and care coordination. As routine practice the discharged patients also received a nurse-initiated telephone call to follow up on their condition and reinforce the self-care and rehabilitation. The nurse would prepare patients for the home-based follow-up including goal-setting throughout the rehabilitation journey. | |  |
|  |  |  | Nurse case manager | Assessment of person-centered goals | Occupational therapists |  |  |  |  |  |
|  |  |  |  | Case management |  |  |  |  |  |  |
|  |  |  |  | Discharge planning | Physical  therapists |  |  |  |  |  |
|  |  |  |  | Education and skills training for caregivers |  |  |  |  |  |  |
|  |  |  |  | Education and skills training for self-care and self-management | PRM  physicians |  |  |  |  |  |
|  |  |  |  | Follow up visits | Speech and language therapists |  |  |  |  |  |
|  |  |  |  | Health status monitoring |  |  |  |  |  |  |
|  |  |  |  | Home visits |  |  |  |  |  |  |
|  |  |  |  | Problem solving skills training |  |  |  |  |  |  |
|  |  |  |  | Rehabilitation coordination and management not further specified |  |  |  |  |  |  |
|  |  |  |  | Therapeutic |  |  |  |  |  |  |
|  |  |  |  | Training for activities of daily living |  |  |  |  |  |  |
| PHC, Home | (Kidd et al., 2015) | United Kingdom | Stroke nurse | Assessment of person-centered goals | None | **Yes** | No | The stroke nurses were specialist community-based practitioners whose roles were to visit stroke survivors at home or in the community following discharge to support the transition between acute and primary care and address the long-term needs of stroke survivors post-discharge. The intervention took the form of a ‘tailored self-management action plan’ designed in a booklet format and created by nurses and stroke survivors working in partnership using a structured self-management assessment questionnaire and goal setting. | |  |
|  |  |  |  | Education and skills training for self-care and self-management |  |  |  |  |  |  |
|  |  |  |  | Home visits |  |  |  |  |  |  |
|  |  |  |  | Motivational interventions |  |  |  |  |  |  |
| PHC, Eldercare  institution / Telerehabilitation | (Kim et al., 2021) | South Korea | Nurse | Assessment of functioning | Social  workers | **Yes** | No | The intervention was conducted by a care team led by onsite SPEC coordinators typically a nurse–social worker pair who were trained and coached by the SPEC consultant. The consultant a nurse trained by the research team was responsible for facilitating and monitoring the implementation process including educating the onsite coordinators to do CGA and CP; demonstrating how to use the SPEC information system; coaching the care team to do ICCs; and being a resource for inquiries about implementation via onsite meetings and also a free messaging app. | |  |
|  |  |  |  | Assessment of frailty |  |  |  |  |  |  |
|  |  |  |  | Assessment of person-centered goals |  |  |  |  |  |  |
|  |  |  |  | Case management |  |  |  |  |  |  |
|  |  |  |  | Education and skills training for caregivers |  |  |  |  |  |  |
| PHC, Community | (King et al., 2018) | New Zealand | Gerontology nurse  specialist | Assessment of functioning | General  practitioners | **Yes** | Yes | The clinical nurse specialist (CNS) role is included as one of the four types of APN disciplines. Data analysis revealed two central themes from the older people perspective: “holistic expertise” and “communication”. Two main themes were identified from the health professional perspective: “competency” and “service delivery.” Results showed the gerontology nurse specialist role was highly regarded by both older people and the health professionals. The in-home CGA was identified as greatly beneficial. | |  |
|  |  |  |  | Assessment of medication |  |  |  |  |  |  |
|  |  |  | Practice nurse | Case management |  |  |  |  |  |  |
|  |  |  |  | Comprehensive geriatric assessment |  |  |  |  |  |  |
|  |  |  |  | Follow up visits |  |  |  |  |  |  |
|  |  |  |  | Monitoring of functional ability |  |  |  |  |  |  |
| Multiple, Outpatient / Inpatient | (Ko et al., 2019) | South Korea | Nurse | Activities of daily living skills training | None | **Yes** | Yes | The nurse was trained on the exercises protocol and safety measures of the individualized transitional care program (ITCP) to help participants repeat exercises daily that is from 1 day after hip arthroplasty to right before discharge. | |  |
|  |  |  |  | Assessment of emotional functions |  |  |  |  |  |  |
|  |  |  |  | Assessment of health status |  |  |  |  |  |  |
|  |  |  |  | Assessment of fall risk |  |  |  |  |  |  |
|  |  |  |  | Assessment of frailty |  |  |  |  |  |  |
|  |  |  |  | Discharge planning |  |  |  |  |  |  |
|  |  |  |  | Education and skills training for self-care and self-management |  |  |  |  |  |  |
|  |  |  |  | Emotional support |  |  |  |  |  |  |
|  |  |  |  | Motivational interventions |  |  |  |  |  |  |
|  |  |  |  | Therapeutic exercises |  |  |  |  |  |  |
| PHC, Home | (Kono et al., 2016) | Japan | Community care nurse | Assessment of cognitive functions | Other | No | No | Routine Preventive home visits (PHVs) were provided every 3 months for 24 months by community care nurses social workers or care managers who worked at all six community-based integrated centers in the three municipalities. | |  |
|  |  |  |  | Assessment of functioning | Social  workers |  |  |  |  |  |
|  |  |  |  | Assessment of person-centered goals |  |  |  |  |  |  |
|  |  |  |  | Monitoring of functional ability |  |  |  |  |  |  |
|  |  |  |  | Rehabilitation coordination and management not further specified |  |  |  |  |  |  |
| Multiple, Outpatient /  Inpatient | (Koolen et al., 2020) | Netherlands | Respiratory nurse | Assessment of person-centered goals | General  practitioners | No | No | The respiratory nurse concentrated on the psychosocial functioning such as mood and social conditions interfering with coping the disease and self-management behaviors like medication use lifestyle factors and coping with exacerbations. | |  |
|  |  |  |  | Behavioral interventions | Occupational therapists |  |  |  |  |  |
|  |  |  |  | Case management | Physical  therapists |  |  |  |  |  |
|  |  |  |  | Education and skills training for self-care and self-management |  |  |  |  |  |  |
|  |  |  |  | Health status monitoring | Psychologists |  |  |  |  |  |
|  |  |  |  | Motivational interventions | Social  workers |  |  |  |  |  |
| PHC, Community | (Leung et al., 2016) | China (Hong Kong) | Rheumatology nurse | Assessment of person-centered goals | None | **Yes** | Yes | A community based ASMP led by trained lay leaders. | |  |
|  |  |  |  | Education and skills training for self-care and self-management |  |  |  |  |  |  |
|  |  |  |  | Peer support or peer support group |  |  |  |  |  |  |
|  |  |  |  | Therapeutic exercises |  |  |  |  |  |  |
| Multiple, Outpatient | (Looman, Huijsman, et al., 2016) | Netherlands | Nurse  practitioner | Assessment of functioning | Other  physicians | No | No | After screening, frail older patients in the experimental group were visited by a nurse practitioner who assessed their functional cognitive mental and psychological functioning using EASYcare. The GP and nurse practitioner decided on treatment goals in consultation with the older people and their informal caregivers which were translated into a preliminary multidisciplinary treatment plan. This plan was determined in a multidisciplinary meeting attended by at least the GP the nurse practitioner and a secondary-line geriatric nurse practitioner. Case management was provided from the GP-practice by the nurse practitioner or by a secondary-line geriatric nursing practitioner depending on the complexity of the older people person’s problems. The model required task reassignment and delegation between nurses and doctors and among GPs nursing home doctors and geriatricians. | |  |
|  |  |  |  | Assessment of person-centered goals |  |  |  |  |  |  |
|  |  |  |  | Monitoring of functional ability |  |  |  |  |  |  |
|  |  |  |  | Rehabilitation coordination and management not further specified |  |  |  |  |  |  |
|  |  |  |  | Multicomponent rehabilitation care or not specified |  |  |  |  |  |  |
| PHC, Outpatient | (Looman, Fabbricotti, et al., 2016) | Netherlands | Nurse  practitioner | Assessment of cognitive functions | General  practitioners | No | No |  |  |  |
|  |  |  | Geriatric nurse  practitioner | Assessment of emotional functions |  |  |  |  |  |  |
|  |  |  |  | Assessment of fall risk | Other |  |  |  |  |  |
|  |  |  | District nurse | Assessment of functioning | Physical  therapists |  |  |  |  |  |
|  |  |  |  | Assessment of medication |  |  |  |  |  |  |
|  |  |  |  | Assessment of person-centered goals |  |  |  |  |  |  |
|  |  |  |  | Case management |  |  |  |  |  |  |
|  |  |  |  | Management of urinary incontinence |  |  |  |  |  |  |
|  |  |  |  | Multicomponent rehabilitation care or not specified |  |  |  |  |  |  |
|  |  |  |  | Rehabilitation coordination and management not further specified |  |  |  |  |  |  |
| PHC, Telerehabilitation | (Lycholip et al., 2018) | Netherlands | Heart failure nurse | Assessment of health status | None | **Yes** | No | The heart failure nurses provided education and guidance of patients. | |  |
|  |  |  |  | Education and skills training for self-care and self-management |  |  |  |  |  |  |
|  |  |  |  | Health status monitoring |  |  |  |  |  |  |
| Multiple, Outpatient | (Mann et al., 2020) | Australia | Enablement Officer | Case management | General  practitioners | No | No | Each patient has been assigned to an Enablement Officer (EO) (allied health or nursing). The service goes to where the client is; that is to the person’s home or the GP clinic. OPEN ARCH provides a comprehensive assessment within the home environment to facilitate depth of understanding of personal circumstances and creation of a personalized care plan. | |  |
|  |  |  |  | Comprehensive geriatric assessment | Occupational therapists |  |  |  |  |  |
|  |  |  |  |  | Other  physicians |  |  |  |  |  |
| PHC, Community / Home | (Markle-Reid et al., 2018) | Canada | Registered nurse (RN) | Assessment of person-centered goals | Dieticians | No | No | The registered nurses did home and telephone visits care coordination and system navigation to link clients to other health care professionals and community services as needed completed and send alerts (e.g. medication depressive symptoms diabetes complications) to communicate concerns with the primary care physician or other providers. | |  |
|  |  |  |  | Case management | Other |  |  |  |  |  |
|  |  |  |  | Education and skills training for self-care and self-management |  |  |  |  |  |  |
|  |  |  |  | Follow up visits |  |  |  |  |  |  |
|  |  |  |  | Home visits |  |  |  |  |  |  |
|  |  |  |  | Motivational interventions |  |  |  |  |  |  |
|  |  |  |  | Peer support or peer support group |  |  |  |  |  |  |
| PHC, Home | (Metzelthin et al., 2015) | Netherlands | Practice nurse | Assessment of frailty | General  practitioners | No | No | After a frailty screening (Step 1) people receive an in-home assessment by the practice nurse (Step 2). In a bilateral (i.e. GP and practice nurse) or extended team meeting (e.g. GP, practice nurse, occupational therapist, and physiotherapist) a preliminary treatment plan is formulated (Step 3). | |  |
|  |  |  |  | Assessment of person-centered goals | Occupational therapists |  |  |  |  |  |
|  |  |  |  | Multicomponent rehabilitation care or not specified | Physical  therapists |  |  |  |  |  |
|  |  |  |  | Rehabilitation coordination and management not further specified |  |  |  |  |  |  |
| Multiple, Inpatient / Outpatient | (Meunier et al., 2016) | United States | Registered nurse (RN) | Assessment of person-centered goals | Dieticians | **Yes** | No | The PACE site was heavily focused on nursing care and staffed with a full-time NP three RNs two licensed practical nurses (LPNs) and several certified nursing assistants (CNAs). The full-time nurse practitioner (NP) who served as a primary care provider. | |  |
|  |  |  | Nurse-practitioner (NP) | Management in the use of medication | General  practitioners |  |  |  |  |  |
|  |  |  | Licensed practical nurse (LPN) | Rehabilitation coordination and management not further specified | Occupational therapists |  |  |  |  |  |
|  |  |  | Certified nursing assistant (CNA) | Social care and support plan | Physical  therapists |  |  |  |  |  |
|  |  |  |  |  | Social  workers |  |  |  |  |  |
| Multiple, Home / Outpatient / Inpatient | (Morri et al., 2021) | Italy | Nurse | Assessment of functioning | Physical  therapists | No | Yes | In the hospital the nurses worked in a multidisciplinary team to evaluate the needs of each patient in terms of physiotherapy and care. The team then establishes the best post-discharge care pathway for each patient. The nurse-researcher collected the basic data of the individuals participating in the study. In home care the community nurses promoted independence in daily living activities such as eating bathing and getting dressed. | |  |
|  |  |  | Community nurse | Multicomponent rehabilitation care or not specified |  |  |  |  |  |  |
|  |  |  | Nurse researcher |  |  |  |  |  |  |  |
| Multiple, Home / Community / Outpatient / Telerehabilitation | (Noh et al., 2021) | Korea | Nurse | Assessment of person-centered goals | Other  physicians | No | Yes | The service was provided by the general manager care manager (nurse or social worker) care navigator (community-dwelling older adults) or cooperative physician at least once depending on the needs of participants and the type of service. | |  |
|  |  |  |  | Case management |  |  |  |  |  |  |
|  |  |  |  | Cognitive training | Social  workers |  |  |  |  |  |
|  |  |  |  | Education and skills training for self-care and self-management |  |  |  |  |  |  |
|  |  |  |  | Emotional support |  |  |  |  |  |  |
|  |  |  |  | Therapeutic recreation |  |  |  |  |  |  |
| Multiple, Inpatient /  Eldercare institution /  Home | (Pannill, 2016) | Netherlands | Community care registered nurse (CCRN) | Comprehensive geriatric assessment | General  practitioners | No | No | The transitional care bridge program included transfer of patient management to a community care registered nurse (CCRN) with in-hospital in-person handover of care plans; an in-hospital CCRN visit with patients; CCRN home or nursing home visits with patients and caregivers after discharge; and handover to General practitionerss at 24 weeks. | |  |
|  |  |  |  | Discharge planning |  |  |  |  |  |  |
|  |  |  |  | Follow up visits |  |  |  |  |  |  |
| Multiple, Inpatient / Home | (Rasmussen et al., 2016) | Denmark | Nurse | Activities of daily living skills training | Occupational therapists | No | No | The nurse participated in the home training if nursing intervention was needed. At least once in the home training period a follow-up visit by the team’s nurse was done giving advice and information to the patient and/or relatives about subjects like stroke sequelae lifestyle (smoking alcohol diet hypertension and more) medication fatigue depression incontinence etc. The nurse helped patients contacting their General practitioners to facilitate follow-up and control visits when needed. | |  |
|  |  |  |  | Assessment of cognitive functions |  |  |  |  |  |  |
|  |  |  |  | Assessment of environment | Other  physicians |  |  |  |  |  |
|  |  |  |  | Assessment of functioning |  |  |  |  |  |  |
|  |  |  |  | Assessment of person-centered goals | Physical  therapists |  |  |  |  |  |
|  |  |  |  | Discharge planning |  |  |  |  |  |  |
|  |  |  |  | Education and skills training for caregivers |  |  |  |  |  |  |
|  |  |  |  | Multicomponent rehabilitation care or not specified |  |  |  |  |  |  |
|  |  |  |  | Provision and training in the use of assistive products |  |  |  |  |  |  |
|  |  |  |  | Rehabilitation coordination and management not further specified |  |  |  |  |  |  |
|  |  |  |  | Social care and support plan | General  practitioners | No | No | A case manager (either a nurse or social worker) was assigned to each participant. Case managers were responsible for the planning and logistics regarding the team meetings and for coordinating and monitoring care. For each participant using 5 chronically prescribed drugs a yearly medication review was held by the GP the nurse and a pharmacist. | |  |
| PHC, Outpatient | (Ruikes et al., 2016) | Netherlands | Practice nurse | Assessment of person-centered goals |  |  |  |  |  |  |
|  |  |  | Community nurse | Assessment of medication | Geriatricians |  |  |  |  |  |
|  |  |  | Nurse | Case management |  |  |  |  |  |  |
|  |  |  |  | Follow up visits |  |  |  |  |  |  |
|  |  |  |  | Health status monitoring |  |  |  |  |  |  |
|  |  |  |  | Monitoring of functional ability |  |  |  |  |  |  |
|  |  |  |  | Rehabilitation coordination and management not further specified |  |  |  |  |  |  |
|  |  |  |  | Social care and support plan |  |  |  |  |  |  |
| PHC, Community | (Shinkai et al., 2016) | Japan | Public health nurse | Comprehensive geriatric assessment | General  practitioners | No | No | The municipal public health professionals (four public health nurses and one nutritionist) shared a common goal and carried out routine tasks such as health education and consultations on healthy aging. The support of the leader in the local government was essential because of the long duration of this type of project and health professionals in the local government such as public health nurses and nutritionists had key roles in the community intervention. | |  |
|  |  |  |  | Education and counseling about healthy lifestyle behaviors |  |  |  |  |  |  |
|  |  |  |  | Education and counseling on physical activity or exercises |  |  |  |  |  |  |
|  |  |  |  | Monitoring of functional ability |  |  |  |  |  |  |
|  |  |  |  | Therapeutic exercises |  |  |  |  |  |  |
|  |  |  |  | Social care and support plan |  |  |  |  |  |  |
| Multiple, Eldercare institution / Home | (Smith & Fields, 2020) | Australia | Registered nurse (RN) | Case management | Dieticians | **Yes** | No | Clinical care provided as part of the transition care program where required is to be carried out by a registered nurse or under the direct or indirect supervision of a registered nurse or other professional appropriate to the service delivery and in accordance with professional standards and guidelines. The role of nurses is clearly defined and described in the "Transition Care Program Guidelines" (2019) by the Australian Government. | |  |
|  |  |  |  | Discharge planning | Occupational therapists |  |  |  |  |  |
|  |  |  |  | Education and skills training for self-care and self-management | Psychologists |  |  |  |  |  |
|  |  |  |  | Multicomponent rehabilitation care or not specified | Social  workers |  |  |  |  |  |
|  |  |  |  | Provision and training in the use of assistive products | Speech and language therapists |  |  |  |  |  |
| PHC, Community | (Sok et al., 2021) | Korea | Geriatric nurse specialist | Cognitive training | None | **Yes** | Yes | The program was verified by an expert group consisting of 1 professor of rehabilitation medicine 1 professor of geriatric internal medicine 1 geriatric nurse specialist and 3 head nurses in the geriatric ward for the validity of contents and process of the cognitive/exercises dual-task program. | |  |
|  |  |  | Head nurse in the geriatric ward | Motivational interventions |  |  |  |  |  |  |
|  |  |  |  | Therapeutic exercises |  |  |  |  |  |  |
| PHC, Home | (Taube et al., 2018) | Sweden | Registered nurse (RN) | Assessment of emotional functions | Physical  therapists | No | No | Case managers (nurses and physiotherapists) provided an intervention of general case management general information specific information and continuity and safety. | |  |
|  |  |  |  | Assessment of fall risk |  |  |  |  |  |  |
|  |  |  |  | Assessment of functioning |  |  |  |  |  |  |
|  |  |  |  | Assessment of health status |  |  |  |  |  |  |
|  |  |  |  | Assessment of medication |  |  |  |  |  |  |
|  |  |  |  | Assessment of person-centered goals |  |  |  |  |  |  |
|  |  |  |  | Case management |  |  |  |  |  |  |
|  |  |  |  | Emotional support |  |  |  |  |  |  |
|  |  |  |  | Follow up visits |  |  |  |  |  |  |
|  |  |  |  | Health status monitoring |  |  |  |  |  |  |
|  |  |  |  | Monitoring of functional ability |  |  |  |  |  |  |
|  |  |  |  | Social care and support plan |  |  |  |  |  |  |
|  |  |  |  | Social skills training |  |  |  |  |  |  |
|  |  |  |  | Therapeutic exercises |  |  |  |  |  |  |
| Multiple, Inpatient / Home | (Tseng et al., 2016) | China | Geriatric nurse | Assessment of emotional functions | Geriatricians | No | No | Geriatric assessment was first delivered by a geriatric nurse to assess and detect potential problems. Rehabilitation included in-hospital rehabilitation starting on the first day following surgery and 4 months of in-home rehabilitation both delivered by a geriatric nurse. The geriatric nurse provided a structured discharge assessment of caregiver competence resources family function elderly subject’s self-care ability elderly subjects’ and their family caregivers’ need for community or long-term care services assessment of the home environment and referrals to community resources referrals. The geriatric nurse assessed fall risks and provided corresponding interventions at each home visit. High risk patients were followed up by a geriatric nurse who provided consultation according to suggestions of the Geriatricians and dietician. The nurse also assessed nutritional outcomes using the MNA at each home visit. Geriatric nurses assessed subjects’ depressive symptoms before hospital discharge and at each home visit. At the same time the geriatric nurse provided individualized consultation and emotional support for these at-risk subjects. | |  |
|  |  |  |  | Assessment of environment |  |  |  |  |  |  |
|  |  |  |  | Assessment of fall risk |  |  |  |  |  |  |
|  |  |  |  | Assessment of functioning |  |  |  |  |  |  |
|  |  |  |  | Assessment of medication |  |  |  |  |  |  |
|  |  |  |  | Comprehensive geriatric assessment |  |  |  |  |  |  |
|  |  |  |  | Discharge planning |  |  |  |  |  |  |
|  |  |  |  | Education and counseling on nutrition |  |  |  |  |  |  |
|  |  |  |  | Follow up visits |  |  |  |  |  |  |
|  |  |  |  | Multicomponent rehabilitation care or not specified |  |  |  |  |  |  |
|  |  |  |  | Psychological interventions |  |  |  |  |  |  |
|  |  |  |  | Social care and support plan |  |  |  |  |  |  |
|  |  |  |  | Therapeutic exercises |  |  |  |  |  |  |
| Multiple, Inpatient / Home | (Tseng et al., 2015) | China | Geriatric nurse | Assessment of family and caregivers' needs knowledge and skills | Geriatricians | No | No | Continuous rehabilitation was delivered by geriatric nurses and Physical therapists to facilitate early postoperative mobility and to provide rehabilitation in the patient’s home setting. The discharge planning component was delivered by geriatric nurses to assure continuity of care. The patient’s adherence to follow ups in clinics was also monitored by the geriatric nurse. During the hospital stay each participant in the intervention program was seen once by a Geriatricians and a rehabilitation physician twice by a Physical therapists and six times by a geriatric nurse. | |  |
|  |  |  |  | Assessment of functioning | Physical  therapists |  |  |  |  |  |
|  |  |  |  | Assessment of person-centered goals |  |  |  |  |  |  |
|  |  |  |  | Comprehensive geriatric assessment | PRM  physicians |  |  |  |  |  |
|  |  |  |  | Discharge planning |  |  |  |  |  |  |
|  |  |  |  | Education and skills training for caregivers |  |  |  |  |  |  |
|  |  |  |  | Education and skills training for self-care and self-management |  |  |  |  |  |  |
|  |  |  |  | Follow up visits |  |  |  |  |  |  |
|  |  |  |  | Multicomponent rehabilitation care or not specified |  |  |  |  |  |  |
|  |  |  |  | Rehabilitation coordination and management not further specified |  |  |  |  |  |  |
|  |  |  |  | Social care and support plan |  |  |  |  |  |  |
|  |  |  |  | Therapeutic exercises |  |  |  |  |  |  |
| Multiple, Inpatient / Home | (Tseng et al., 2021) | Taiwan | Geriatric nurse | Assessment of person-centered goals | None | Yes | Yes | Geriatric assessment: delivered by geriatric nurse and Geriatricians with involvement of family caregivers. Discharge planning: geriatric nurse made a pre-discharge assessment with necessary referrals and after discharge home environment assessment. Rehabilitation program: including both in-hospital and in-home progressed rehabilitation supervised by geriatric nurse with involvement of family caregivers. The geriatric nurse taught the in-home rehabilitation. Family caregiver training: delivered by geriatric nurse to help the family caregivers providing hip fracture and dementia care. | |  |
|  |  |  |  | Comprehensive geriatric assessment |  |  |  |  |  |  |
|  |  |  |  | Discharge planning |  |  |  |  |  |  |
|  |  |  |  | Education and skills training for caregivers |  |  |  |  |  |  |
|  |  |  |  | Home visits |  |  |  |  |  |  |
|  |  |  |  | Therapeutic exercises |  |  |  |  |  |  |
| PHC, Community | (Uittenbroek et al., 2017) | Netherlands | Nurse | Assessment of person-centered goals | General  practitioners | No | No | For Embrace a GP-led Elderly Care Team was assembled in which the GP remained responsible for writing prescriptions and implementing the interventions. | |  |
|  |  |  | District nurse | Education and skills training for self-care and self-management |  |  |  |  |  |  |
|  |  |  | Community nurse | Follow up visits | Geriatricians |  |  |  |  |  |
|  |  |  |  | Health status monitoring | Social  workers |  |  |  |  |  |
|  |  |  |  | Monitoring of functional ability |  |  |  |  |  |  |
|  |  |  |  | Rehabilitation coordination and management not further specified |  |  |  |  |  |  |
|  |  |  |  | Social care and support plan |  |  |  |  |  |  |
| PHC, Telerehabilitation | (Valdivieso et al., 2018) | Spain | Nurse | Education and skills training for caregivers | None | Yes | No | The telephone support program is lead collaboratively by primary care teams and by hospital case manager nurses working in a Telemedicine Unit and is based on the preparation of personalized care plans for every patient that includes monitoring medication control surveys specific educational information for patients and caregivers and a health agenda. | |  |
|  |  |  | Hospital case manager nurse | Education and skills training for self-care and self-management |  |  |  |  |  |  |
|  |  |  |  | Follow up visits |  |  |  |  |  |  |
|  |  |  | Recruiting nurse | Health status monitoring |  |  |  |  |  |  |
|  |  |  |  | Social care and support plan |  |  |  |  |  |  |
| Multiple, Telerehabilitation | (Wolf et al., 2016) | Sweden | Registered nurse (RN) | Education and skills training for self-care and self-management | Other  physicians | No | No | An introductory demonstration which required the patient to test the eHealth tools was provided by a registered nurse who was familiar with the study so that patients could start using the tools freely during their hospital stay. | |  |
|  |  |  |  | Health status monitoring |  |  |  |  |  |  |
|  |  |  |  | Monitoring of functional ability |  |  |  |  |  |  |
|  |  |  |  | Rehabilitation coordination and management not further specified |  |  |  |  |  |  |
| PHC, Community / Home | (A. K. C. Wong et al., 2019) | China (Hong Kong) | Registered nurse case manager (NCM) | Assessment of environment | Community health worker | **Yes** | No | The providers included a health-social care team led by a registered nurse case manager (NCM) and supported by community workers (CWs) and social workers (SWs). The health-social partnership team in the community promotes ageing in place. It was recognized that the NCM can provide strong leadership and help to integrate others’ work. | |  |
|  |  |  |  | Assessment of functioning |  |  |  |  |  |  |
|  |  |  |  | Assessment of person-centered goals | Social  workers |  |  |  |  |  |
|  |  |  |  | Case management |  |  |  |  |  |  |
|  |  |  |  | Education and skills training for self-care and self-management |  |  |  |  |  |  |
|  |  |  |  | Follow up visits |  |  |  |  |  |  |
| PHC, Outpatient | (Zakrisson et al., 2016) | Sweden | Asthma / COPD nurse | Education and counseling about healthy lifestyle behaviors | Dieticians | **Yes** | No | Almost all PHC centers in Sweden have an asthma / COPD clinic led by a specialized asthma / COPD nurse and mainly provide individual consultations. Nurses led meetings about disease and medication anatomy and physiology and about nutritional advice. This paper adds that one program is not enough there is a need for regular follow-ups in PHC. Also there is a difference in which nurses deliver interventions in which settings (more specialized health workers in the hospital). | |  |
|  |  |  | Primary health care nurse | Education and skills training for self-care and self-management | Occupational therapists |  |  |  |  |  |
|  |  |  |  | Education and skills training for caregivers | Physical  therapists |  |  |  |  |  |
|  |  |  |  |  | Social  workers |  |  |  |  |  |
| PHC,  Home / Community | (L. Zhang et al., 2017) | China | Nurse | Assessment of emotional functions | Dieticians | No | No | Nurses or community physicians had the responsibility for designing the training modules and providing review and guidance to the program administrators. | |  |
|  |  |  | Rehabilitation nurse | Assessment of functioning | Other  physicians |  |  |  |  |  |
|  |  |  | Cardiac nurse | Education and counseling about healthy lifestyle behaviors | Physical  therapists |  |  |  |  |  |
|  |  |  | Community nurse | Education and skills training for caregivers |  |  |  |  |  |  |
|  |  |  |  | Follow up visits | Psychologists |  |  |  |  |  |
|  |  |  |  | Peer support or peer support group |  |  |  |  |  |  |
|  |  |  |  | Therapeutic exercises |  |  |  |  |  |  |
| Multiple, Outpatient /  Inpatient | (P. Zhang et al., 2018) | China | Community nurse | Assessment of functioning | None | Yes | No | Nurse-led transitional care program in addition to usual care consisting of two phases: 1) predischarge phase (one week before discharge) with special assessments and health education and a booklet with information to consolidate knowledge and 2) post discharge phase (7 months) with four intervention schemes addressing health behaviors and health promotion. | |  |
|  |  |  | Nurse | Case management |  |  |  |  |  |  |
|  |  |  | Cardiovascular nurse | Discharge planning |  |  |  |  |  |  |
|  |  |  | Postgraduated nursing student | Education and counseling about healthy lifestyle behaviors |  |  |  |  |  |  |
|  |  |  |  | Education and skills training for self-care and self-management |  |  |  |  |  |  |
|  |  |  |  | Follow up visits |  |  |  |  |  |  |
|  | **Studies with nurses in a clinical role (N=12)** | | | | | | | | | |
| PHC, Outpatient | (Barker et al., 2016) | Australia | Rehabilitation nurse | Multicomponent rehabilitation care or not specified | Dieticians | No | No | Community Rehabilitation northern Queensland (CRnQ) service. | |  |
|  |  |  |  |  | Occupational therapists |  |  |  |  |  |
|  |  |  |  |  | Physical  therapists |  |  |  |  |  |
|  |  |  |  |  | Social  workers |  |  |  |  |  |
|  |  |  |  |  | Speech and language therapists |  |  |  |  |  |
| Multiple, Inpatient /  Outpatient | (Duncan et al., 2018) | United States | Nurse  coordinator | Assessment of person-centered goals | General  practitioners | No | No | The nurse administered the web-based PRO questionnaires to the patient or proxy at 2 time points over the phone and in person. | |  |
|  |  |  | Nurse  practitioner |  | Occupational therapists |  |  |  |  |  |
|  |  |  | Nurse |  | Physical  therapists |  |  |  |  |  |
|  |  |  |  |  | Speech and language therapists |  |  |  |  |  |
| PHC, Community | (Godtfredsen et al., 2018) | Denmark | Nurse | Education and counseling on physical activity or exercises | Dieticians | No | No | Healthcare centers were led by multidisciplinary staff without doctors. | |  |
|  |  |  |  |  | General  practitioners |  |  |  |  |  |
|  |  |  |  |  | Physical  therapists |  |  |  |  |  |
| PHC, Outpatient /  Community | (Inzitari et al., 2018) | Spain | Primary care nurse | Follow up visits | General  practitioners | No | No | Nurses promoted the achievement of shared goals and fostered empowerment during the follow-up visits in agreement of the community care approach. | |  |
|  |  |  |  |  | Geriatricians |  |  |  |  |  |
|  |  |  |  |  | Physical  therapists |  |  |  |  |  |
|  |  |  |  |  | Social  workers |  |  |  |  |  |
| Multiple, Inpatient / Outpatient | (Lindhardt et al., 2019) | Denmark | Municipality nurse | Behavioral interventions | General  practitioners | No | No | Follow-up was carried out in the patient’s home by a nurse. The patient’s GP and the municipality preventive consultant received a structured communication report with the assessment results prepared by the nurses who carried out the intervention. The municipality preventive consultant phoned the patients at home and provided information about relevant municipality activities they could join. Patients in this group took part in a motivational interview with an experienced municipality nurse (S.M.L.) skilled in this technique | |  |
|  |  |  | Staff nurse | Education and skills training for self-care and self-management |  |  |  |  |  |  |
|  |  |  |  | Follow up visits |  |  |  |  |  |  |
|  |  |  |  | Motivational interventions |  |  |  |  |  |  |
| PHC, Home | (Mas et al., 2016) | Spain | Nurse | Multicomponent rehabilitation care or not specified | Occupational therapists | No | No | Medical care was provided by Geriatricians. Nursing intervention was pivoting on geriatric care needs (based on cognitive nutritional and skin care protocols). | |  |
|  |  |  |  |  | Other  physicians |  |  |  |  |  |
|  |  |  |  |  | Physical  therapists |  |  |  |  |  |
| PHC, Outpatient | (Mosleh et al., 2015) | United Kingdom (Scotland) | Cardiac rehabilitation nurse | Education and skills training for self-care and self-management | Dieticians | No | No | The program is run by a multidisciplinary team (a cardiac rehabilitation nurse a physiotherapist and a Dieticians) and a psychologist and physician are available if needed. | |  |
|  |  |  |  | Therapeutic exercises | Physical  therapists |  |  |  |  |  |
|  |  |  |  |  | Psychologists |  |  |  |  |  |
| PHC, Outpatient | (Tarazona-Santabalbina et al., 2016) | Spain | Nurse | Health status monitoring | Physical  therapists | No | No | The intervention was performed by 8 experienced physiotherapists or nurses. | |  |
|  |  |  |  | Therapeutic exercises |  |  |  |  |  |  |
| PHC, Telerehabilitation | (van der Weegen et al., 2015) | Netherlands | Practice nurse (PN) | Assessment of person-centered goals | None | No | No | In the first consultation the PN raised awareness about the risks of physical inactivity and the PA level of the patient was discussed using the previously completed SQUASH questionnaire. In addition participants received a general and a disease-specific pamphlet about PA. During the second consultation a personal goal was set in minutes of activity per day based on the pre-measurement and the PN encouraged the participants to set up an activity plan to reach personal goals. In the last consultation activity results barriers facilitators and PA habits were evaluated and how the PN and patient would continue the lifestyle coaching was discussed. | |  |
|  |  |  |  | Education and counseling on physical activity or exercises |  |  |  |  |  |  |
|  |  |  |  | Education and skills training for self-care and self-management |  |  |  |  |  |  |
|  |  |  |  | Motivational interventions |  |  |  |  |  |  |
|  |  |  |  | Monitoring of functional ability |  |  |  |  |  |  |
| PHC, Outpatient | (van Dijk-de Vries et al., 2015) | Netherlands | Practice nurse | Assessment of emotional functions | None | No | Yes | After the training sessions Practice nurses (PNs) started to integrate SMS into their routine care practice. They applied SMS in all their consultations with patients with diabetes. SMS included a detection and follow-up phase. In European countries most patients with diabetes receive follow-up care in the primary care setting by nurses.10 Practice nurses (PNs) in the Netherlands work according to guidelines that focus on medical and behavioral management. | |  |
|  |  |  |  | Assessment of functioning |  |  |  |  |  |  |
|  |  |  |  | Assessment of person-centered goals |  |  |  |  |  |  |
|  |  |  |  | Education and skills training for self-care and self-management |  |  |  |  |  |  |
|  |  |  |  | Emotional support |  |  |  |  |  |  |
|  |  |  |  | Problem solving skills training |  |  |  |  |  |  |
| PHC, Community | (van Lieshout et al., 2018) | Netherlands | Nurse | Cognitive training | Dieticians | No | No | A community nurse performed the meetings with additional psychosocial training at a local community center. | |  |
|  |  |  | Community nurse | Education and skills training for self-care and self-management | Physical  therapists |  |  |  |  |  |
|  |  |  | Psychosocial nurse | Problem-solving skills training |  |  |  |  |  |  |
|  |  |  |  | Social skills training |  |  |  |  |  |  |
| PHC, Outpatient /  Community | (Woo et al., 2021) | China (Hong Kong) | Nurse | Assessment of fall risk | Dieticians | No | No | The medical component for these services (doctors nurses allied health) is variable and may be absent. Screening consultation (optometry physiotherapy nutritionist nurse) group program activities rehabilitation and day care are paid for by users. | |  |
|  |  |  |  | Assessment of person-centered goals | Optometrists |  |  |  |  |  |
|  |  |  |  | Cognitive training | Physical therapists |  |  |  |  |  |
|  |  |  |  | Social care and support plan |  |  |  |  |  |  |
|  |  |  |  | Therapeutic exercises |  |  |  |  |  |  |
|  |  |  |  | Training for activities of daily living |  |  |  |  |  |  |
| **Studies where nurses’ role was not specified (N=4)** | | | | | | | | | |  |
| PHC, Community | (Lou et al., 2015) | China | Respiratory nurse | Multicomponent rehabilitation care or not specified | Dieticians | No | No | Not specified | |  |
|  |  |  |  |  | General  practitioners |  |  |  |  |  |
|  |  |  |  |  | PRM  physicians |  |  |  |  |  |
| PHC, Home /  Outpatient | (Oh et al., 2021) | South Korea | Nurse | Multicomponent rehabilitation care or not specified | Dieticians | No | No | The multidisciplinary team consisting of doctors, physical education professionals, nurses, nutritionists and exercises experts. | |  |
|  |  |  |  |  | Exercises professionals |  |  |  |  |  |
|  |  |  |  |  | Other  physicians |  |  |  |  |  |
| Multiple, Tele-rehabilitation | (Piette et al., 2015) | United States | Nurse | Multicomponent rehabilitation care or not specified | Other  physicians | No | Yes | The IVR calls were developed by a panel including primary care physicians cardiologists nurses and experts in health behavior change and mHealth. | |  |
| Multiple, Outpatient /  Inpatient | (Quigley et al., 2021) | Australia | Enablement Officer | Multicomponent rehabilitation care or not specified | Geriatricians | No | No | Older people are referred from general practice and assigned an enablement officer (EO; nursing or allied health professional) and Geriatricians. | |  |
|  |  |  |  |  | General  practitioners |  |  |  |  |  |

**References**

Barker, R. N., Sealey, C. J., Polley, M. L., Mervin, M. C., & Comans, T. (2016). Impact of a person-centred community rehabilitation service on outcomes for individuals with a neurological condition. *Disability and Rehabilitation*, *39*(11), 1136–1142. https://doi.org/10.1080/09638288.2016.1185803

Bekelman, D. B., Plomondon, M. E., Carey, E. P., Sullivan, M. D., Nelson, K. M., Hattler, B., McBryde, C. F., Lehmann, K. G., Gianola, K., Heidenreich, P. A., & Rumsfeld, J. S. (2015). Primary Results of the Patient-Centered Disease Management (PCDM) for Heart Failure Study: A Randomized Clinical Trial. *JAMA Internal Medicine*, *175*(5), 725–732. https://doi.org/10.1001/jamainternmed.2015.0315

Bleijenberg, N., Drubbel, I., Schuurmans, M. J., Dam, H. T., Zuithoff, N. P., Numans, M. E., & de Wit, N. J. (2016). Effectiveness of a Proactive Primary Care Program on Preserving Daily Functioning of Older People: A Cluster Randomized Controlled Trial. *Journal of the American Geriatrics Society*, *64*(9), 1779–1788. https://doi.org/10.1111/jgs.14325

Bleijenberg, N., Imhof, L., Mahrer-Imhof, R., Wallhagen, M. I., de Wit, N. J., & Schuurmans, M. J. (2017). Patient Characteristics Associated With a Successful Response to Nurse-Led Care Programs Targeting the Oldest-Old: A Comparison of Two RCTs. *Worldviews on Evidence-Based Nursing*, *14*(3), 210–222. https://doi.org/10.1111/wvn.12235

Blom, J., den Elzen, W., van Houwelingen, A. H., Heijmans, M., Stijnen, T., Van den Hout, W., & Gussekloo, J. (2016). Effectiveness and cost-effectiveness of a proactive, goal-oriented, integrated care model in general practice for older people. A cluster randomised controlled trial: Integrated Systematic Care for older People—the ISCOPE study. *Age and Ageing*, *45*(1), 30–41. https://doi.org/10.1093/ageing/afv174

Buurman, B. M., Parlevliet, J. L., Allore, H. G., Blok, W., van Deelen, B. A., Moll van Charante, E. P., de Haan, R. J., & de Rooij, S. E. (2016). Comprehensive Geriatric Assessment and Transitional Care in Acutely Hospitalized Patients: The Transitional Care Bridge Randomized Clinical Trial. *JAMA Internal Medicine*, *176*(3), 302–309. https://doi.org/10.1001/jamainternmed.2015.8042

Cameron-Tucker, H. L., Wood-Baker, R., Joseph, L., Walters, J. A., Schuz, N., & Walters, E. H. (2016). A randomized controlled trial of telephone-mentoring with home-based walking preceding rehabilitation in COPD. *International Journal of Chronic Obstructive Pulmonary Disease/International Journal of COPD*, *11*, 1991–2000. https://doi.org/10.2147/COPD.S109820

Clevenger, C. K., Cellar, J., Kovaleva, M., Medders, L., & Hepburn, K. (2018). Integrated Memory Care Clinic: Design, Implementation, and Initial Results. *Journal of the American Geriatrics Society*, *66*(12), 2401–2407. https://doi.org/10.1111/jgs.15528

Coskun, S., & Duygulu, S. (2022). The effects of Nurse Led Transitional Care Model on elderly patients undergoing open heart surgery: A randomized controlled trial. *European Journal of Cardiovascular Nursing*, *21*(1), 46–55. https://doi.org/10.1093/eurjcn/zvab005

Coventry, P., Lovell, K., Dickens, C., Bower, P., Chew-Graham, C., McElvenny, D., Hann, M., Cherrington, A., Garrett, C., Gibbons, C. J., Baguley, C., Roughley, K., Adeyemi, I., Reeves, D., Waheed, W., & Gask, L. (2015). Integrated primary care for patients with mental and physical multimorbidity: Cluster randomised controlled trial of collaborative care for patients with depression comorbid with diabetes or cardiovascular disease. *BMJ*, *350*, h638–h638. https://doi.org/10.1136/bmj.h638

Deng, A., Yang, S., & Xiong, R. (2020). Effects of an integrated transitional care program for stroke survivors living in a rural community: A randomized controlled trial. *Clinical Rehabilitation*, *34*(4), 524–532. https://doi.org/10.1177/0269215520905041

Duncan, P. W., Abbott, R. M., Rushing, S., Johnson, A. M., Condon, C. N., Lycan, S. L., Lutz, B. J., Cummings, D. M., Pastva, A. M., D’Agostino, R. B., Stafford, J. M., Amoroso, R. M., Jones, S. B., Psioda, M. A., Gesell, S. B., Rosamond, W. D., Prvu-Bettger, J., Sissine, M. E., Boynton, M. D., … Compass Investigative Team. (2018). COMPASS-CP: An Electronic Application to Capture Patient-Reported Outcomes to Develop Actionable Stroke and Transient Ischemic Attack Care Plans. *Circulation. Cardiovascular Quality and Outcomes*, *11*(8). https://doi.org/10.1161/CIRCOUTCOMES.117.004444

Ekelund, C., & Eklund, K. (2015). Longitudinal effects on self-determination in the RCT “Continuum of care for frail elderly people”. *Quality in Ageing and Older Adults*, *16*(3), 165–176. https://doi.org/10.1108/QAOA-12-2014-0045

Everink, I. H. J., van Haastregt, J. C. M., Maessen, J. M. C., Schols, J. M. G. A., & Kempen, G. I. J. M. (2017). Process evaluation of an integrated care pathway in geriatric rehabilitation for people with complex health problems. *BMC Health Services Research*, *17*(1), 34. https://doi.org/10.1186/s12913-016-1974-5

Everink, I. H. J., van Haastregt, J. C. M., Tan, F. E. S., Schols, J. M. G. A., & Kempen, G. I. J. M. (2018). The effectiveness of an integrated care pathway in geriatric rehabilitation among older patients with complex health problems and their informal caregivers: A prospective cohort study. *BMC Geriatrics*, *18*(1), 285. https://doi.org/10.1186/s12877-018-0971-4

Finlayson, K., Chang, A. M., Courtney, M. D., Edwards, H. E., Parker, A. W., Hamilton, K., Pham, T. D. X., & O’Brien, J. (2018). Transitional care interventions reduce unplanned hospital readmissions in high-risk older adults. *BMC Health Services Research*, *18*(1). https://doi.org/10.1186/s12913-018-3771-9

Franse, C. B., van Grieken, A., Alhambra-Borrás, T., Valía-Cotanda, E., van Staveren, R., Rentoumis, T., Markaki, A., Bilajac, L., Vasiljev Marchesi, V., Rukavina, T., Verma, A., Williams, G., Koppelaar, E., Martijn, R., Voorham, A. J. J., Mattace Raso, F., Garcés-Ferrer, J., & Raat, H. (2018). The effectiveness of a coordinated preventive care approach for healthy ageing (UHCE) among older persons in five European cities: A pre-post controlled trial. *International Journal of Nursing Studies*, *88*, 153–162. https://doi.org/10.1016/j.ijnurstu.2018.09.006

Godtfredsen, N., Sorensen, T. B., Lavesen, M., Pors, B., Dalsgaard, L. S., Dollerup, J., & Grann, O. (2018). Effects of community-based pulmonary rehabilitation in 33 municipalities in Denmark—Results from the KOALA project. *International Journal of Chronic Obstructive Pulmonary Disease/International Journal of COPD*, *14*, 93–100. https://doi.org/10.2147/COPD.S190423

He, M., Wang, J., Dong, Q., Ji, N., Meng, P., Liu, N., Geng, S., Qin, S., Xu, W., Zhang, C., Li, D., Zhang, H., Zhu, J., Qin, H., Hui, R., & Wang, Y. (2018). Community-based stroke system of care improves patient outcomes in Chinese rural areas. *Journal of Epidemiology and Community Health*, *72*(7), 630–635. https://doi.org/10.1136/jech-2017-210185

Hoedemakers, M., Karimi, M., Leijten, F., Goossens, L., Islam, K., Tsiachristas, A., & Rutten-van Molken, M. (2022). Value-based person-centred integrated care for frail elderly living at home: A quasi-experimental evaluation using multicriteria decision analysis. *BMJ Open*, *12*(4), e054672. https://doi.org/10.1136/bmjopen-2021-054672

Inzitari, M., Pérez, L. M., Enfedaque, M. B., Soto, L., Díaz, F., Gual, N., Martín, E., Orfila, F., Mulero, P., Ruiz, R., & Cesari, M. (2018). Integrated primary and geriatric care for frail older adults in the community: Implementation of a complex intervention into real life. *European Journal of Internal Medicine*, *56*, 57–63. https://doi.org/10.1016/j.ejim.2018.07.022

Kidd, L., Lawrence, M., Booth, J., Rowat, A., & Russell, S. (2015). Development and evaluation of a nurse-led, tailored stroke self-management intervention. *BMC Health Services Research*, *15*, 359. https://doi.org/10.1186/s12913-015-1021-y

Kim, H., Jung, Y. I., Kim, G. S., Choi, H., & Park, Y. H. (2021). Effectiveness of a Technology-Enhanced Integrated Care Model for Frail Older People: A Stepped-Wedge Cluster Randomized Trial in Nursing Homes. *The Gerontologist*, *61*(3), 460–469. https://doi.org/10.1093/geront/gnaa090

King, A. I. I., Boyd, M. L., Dagley, L., & Raphael, D. L. (2018). Implementation of a gerontology nurse specialist role in primary health care: Health professional and older adult perspectives. *Journal of Clinical Nursing*, *27*(3–4), 807–818. https://doi.org/10.1111/jocn.14110

Ko, Y., Lee, J., Oh, E., Choi, M., Kim, C., Sung, K., & Baek, S. (2019). Older Adults With Hip Arthroplasty: An Individualized Transitional Care Program. *Rehabil Nurs*, *44*(4), 203–212. https://doi.org/10.1097/rnj.0000000000000120

Kono, A., Izumi, K., Yoshiyuki, N., Kanaya, Y., & Rubenstein, L. Z. (2016). Effects of an Updated Preventive Home Visit Program Based on a Systematic Structured Assessment of Care Needs for Ambulatory Frail Older Adults in Japan: A Randomized Controlled Trial. *J Gerontol A Biol Sci Med Sci*, *71*(12), 1631–1637. https://doi.org/10.1093/gerona/glw068

Koolen, E. H., van den Borst, B., de Man, M., Antons, J. C., Robberts, B., Dekhuijzen, P. N. R., Vercoulen, J. H., van den Heuvel, M., Spruit, M. A., van der Wees, P. J., & van ’t Hul, A. J. (2020). The clinical effectiveness of the COPDnet integrated care model. *Respiratory Medicine*, *172*, 106–152. https://doi.org/10.1016/j.rmed.2020.106152

Leung, Y. Y., Kwan, J., Chan, P., Poon, P. K., Leung, C., Tam, L. S., Li, E. K., & Kwok, A. (2016). A pilot evaluation of Arthritis Self-Management Program by lay leaders in patients with chronic inflammatory arthritis in Hong Kong. *Clin Rheumatol*, *35*(4), 935–941. https://doi.org/10.1007/s10067-014-2791-z

Lindhardt, T., Loevgreen, S. M., Bang, B., Bigum, C., & Klausen, T. W. (2019). A targeted assessment and intervention at the time of discharge reduced the risk of readmissions for short-term hospitalized older patients: A randomized controlled study. *Clin Rehabil*, *33*(9), 1431–1444. https://doi.org/10.1177/0269215519845032

Looman, W. M., Fabbricotti, I. N., de Kuyper, R., & Huijsman, R. (2016). The effects of a pro-active integrated care intervention for frail community-dwelling older people: A quasi-experimental study with the GP-practice as single entry point. *BMC Geriatrics*, *16*, 43. https://doi.org/10.1186/s12877-016-0214-5

Looman, W. M., Huijsman, R., Bouwmans-Frijters, C. A. M., Stolk, E. A., & Fabbricotti, I. N. (2016). Cost-effectiveness of the ‘Walcheren Integrated Care Model’ intervention for community-dwelling frail elderly. *Family Practice*, *33*(2), 154–160. https://doi.org/10.1093/fampra/cmv106

Lou, P., Chen, P., Zhang, P., Yu, J., Wang, Y., Chen, N., Zhang, L., Wu, H., & Zhao, J. (2015). A COPD health management program in a community-based primary care setting: A randomized controlled trial. *Respir Care*, *60*(1), 102–112. https://doi.org/10.4187/respcare.03420

Lycholip, E., Thon Aamodt, I., Lie, I., Simbelyte, T., Puronaite, R., Hillege, H., de Vries, A., Kraai, I., Stromberg, A., Jaarsma, T., & Celutkiene, J. (2018). The dynamics of self-care in the course of heart failure management: Data from the IN TOUCH study. *Patient Prefer Adherence*, *12*, 1113–1122. https://doi.org/10.2147/PPA.S162219

Mann, J., Quigley, R., Harvey, D., Tait, M., Williams, G., & Strivens, E. (2020). OPEN ARCH: Integrated care at the primary–secondary interface for the community-dwelling older person with complex needs. *Australian Journal of Primary Health*, *26*(2), 104. https://doi.org/10.1071/PY19184

Markle-Reid, M., Ploeg, J., Fraser, K. D., Fisher, K. A., Bartholomew, A., Griffith, L. E., Miklavcic, J., Gafni, A., Thabane, L., & Upshur, R. (2018). Community Program Improves Quality of Life and Self-Management in Older Adults with Diabetes Mellitus and Comorbidity. *J Am Geriatr Soc*, *66*(2), 263–273. https://doi.org/10.1111/jgs.15173

Mas, M. A., Closa, C., Santaeugenia, S. J., Inzitari, M., Ribera, A., & Gallofre, M. (2016). Hospital-at-home integrated care programme for older patients with orthopaedic conditions: Early community reintegration maximising physical function. *Maturitas*, *88*, 65–69. https://doi.org/10.1016/j.maturitas.2016.03.005

Metzelthin, S. F., van Rossum, E., Hendriks, M. R., De Witte, L. P., Hobma, S. O., Sipers, W., & Kempen, G. I. (2015). Reducing disability in community-dwelling frail older people: Cost-effectiveness study alongside a cluster randomised controlled trial. *Age Ageing*, *44*(3), 390–396. https://doi.org/10.1093/ageing/afu200

Meunier, M. J., Brant, J. M., Audet, S., Dickerson, D., Gransbery, K., & Ciemins, E. L. (2016). Life after PACE (Program of All-Inclusive Care for the Elderly): A retrospective/prospective, qualitative analysis of the impact of closing a nurse practitioner centered PACE site. *J Am Assoc Nurse Pract*, *28*(11), 596–603. https://doi.org/10.1002/2327-6924.12379

Morri, M., Forni, C., Guberti, M., Chiari, P., Pecorari, A., Orlandi, A. M., Gazineo, D., Bozzo, M., & Ambrosi, E. (2021). Post-hospital care pathway for individuals with hip fracture: What is the optimal setting and rehabilitation intensity? An observational study. *Disabil Rehabil*, 1–8. https://doi.org/10.1080/09638288.2021.1897692

Mosleh, S. M., Bond, C. M., Lee, A. J., Kiger, A., & Campbell, N. C. (2015). Effects of community based cardiac rehabilitation: Comparison with a hospital-based programme. *Eur J Cardiovasc Nurs*, *14*(2), 108–116. https://doi.org/10.1177/1474515113519362

Noh, E.-Y., Park, Y.-H., Cho, B., Huh, I., Lim, K.-C., Ryu, S. I., Han, A.-R., & Lee, S. (2021). Effectiveness of a community-based integrated service model for older adults living alone: A nonrandomized prospective study. *Geriatric Nursing*, *42*(6), 1488–1496. https://doi.org/10.1016/j.gerinurse.2021.10.006

Oh, S.-L., Kim, D.-Y., Bae, J.-H., & Lim, J.-Y. (2021). Effects of rural community-based integrated exercise and health education programs on the mobility function of older adults with knee osteoarthritis. *Aging Clinical and Experimental Research*, *33*(11), 3005–3014. https://doi.org/10.1007/s40520-020-01474-7

Pannill, F. C. (2016). In older hospitalized patients, adding transitional care to in-hospital geriatric assessment did not improve ADL. *Ann Intern Med*, *164*(12), JC63. https://doi.org/10.7326/ACPJC-2016-164-12-063

Piette, J. D., Striplin, D., Marinec, N., Chen, J., Trivedi, R. B., Aron, D. C., Fisher, L., & Aikens, J. E. (2015). A Mobile Health Intervention Supporting Heart Failure Patients and Their Informal Caregivers: A Randomized Comparative Effectiveness Trial. *J Med Internet Res*, *17*(6), e142. https://doi.org/10.2196/jmir.4550

Quigley, R., Russell, S., Harvey, D., & Mann, J. (2021). OPEN ARCH integrated care model: Experiences of older Australians and their carers. *Aust J Prim Health*, *27*(3), 236–242. https://doi.org/10.1071/PY20203

Rasmussen, R. S., Ostergaard, A., Kjaer, P., Skerris, A., Skou, C., Christoffersen, J., Seest, L. S., Poulsen, M. B., Ronholt, F., & Overgaard, K. (2016). Stroke rehabilitation at home before and after discharge reduced disability and improved quality of life: A randomised controlled trial. *Clin Rehabil*, *30*(3), 225–236. https://doi.org/10.1177/0269215515575165

Ruikes, F. G. H., Zuidema, S. U., Akkermans, R. P., Assendelft, W. J. J., Schers, H. J., & Koopmans, R. T. C. M. (2016). Multicomponent Program to Reduce Functional Decline in Frail Elderly People: A Cluster Controlled Trial. *The Journal of the American Board of Family Medicine*, *29*(2), 209–217. https://doi.org/10.3122/jabfm.2016.02.150214

Shinkai, S., Yoshida, H., Taniguchi, Y., Murayama, H., Nishi, M., Amano, H., Nofuji, Y., Seino, S., & Fujiwara, Y. (2016). Public health approach to preventing frailty in the community and its effect on healthy aging in Japan. *Geriatr Gerontol Int*, *16 Suppl 1*, 87–97. https://doi.org/10.1111/ggi.12726

Smith, H. N., & Fields, S. M. (2020). Changes in older adults’ impairment, activity, participation and wellbeing as measured by the AusTOMs following participation in a Transition Care Program. *Aust Occup Ther J*, *67*(6), 517–527. https://doi.org/10.1111/1440-1630.12667

Sok, S., Shin, E., Kim, S., & Kim, M. (2021). Effects of Cognitive/Exercise Dual-Task Program on the Cognitive Function, Health Status, Depression, and Life Satisfaction of the Elderly Living in the Community. *International Journal of Environmental Research and Public Health*, *18*(15), 7848. https://doi.org/10.3390/ijerph18157848

Tarazona-Santabalbina, F. J., Gómez-Cabrera, M. C., Pérez-Ros, P., Martínez-Arnau, F. M., Cabo, H., Tsaparas, K., Salvador-Pascual, A., Rodriguez-Mañas, L., & Viña, J. (2016). A Multicomponent Exercise Intervention that Reverses Frailty and Improves Cognition, Emotion, and Social Networking in the Community-Dwelling Frail Elderly: A Randomized Clinical Trial. *Journal of the American Medical Directors Association*, *17*(5), 426–433. https://doi.org/10.1016/j.jamda.2016.01.019

Taube, E., Kristensson, J., Midlov, P., & Jakobsson, U. (2018). The use of case management for community-dwelling older people: The effects on loneliness, symptoms of depression and life satisfaction in a randomised controlled trial. *Scand J Caring Sci*, *32*(2), 889–901. https://doi.org/10.1111/scs.12520

Tseng, M.-Y., Liang, J., Shyu, Y.-I. L., Wu, C.-C., Cheng, H.-S., Chen, C.-Y., & Yang, S.-F. (2016). Effects of interventions on trajectories of health-related quality of life among older patients with hip fracture: A prospective randomized controlled trial. *BMC Musculoskeletal Disorders*, *17*, 114. https://doi.org/10.1186/s12891-016-0958-2

Tseng, M.-Y., Shyu, Y.-I. L., Liang, J., & Tsai, W.-C. (2015). Interdisciplinary intervention reduced the risk of being persistently depressive among older patients with hip fracture. *Geriatr Gerontol Int*, *16*(10), 1145–1152. https://doi.org/10.1111/ggi.12617

Tseng, M.-Y., Yang, C.-T., Liang, J., Huang, H.-L., Kuo, L.-M., Wu, C.-C., Cheng, H.-S., Chen, C.-Y., Hsu, Y.-H., Lee, P.-C., & Shyu, Y.-I. L. (2021). A family care model for older persons with hip-fracture and cognitive impairment: A randomized controlled trial. *International Journal of Nursing Studies*, *120*, 103995. https://doi.org/10.1016/j.ijnurstu.2021.103995

Uittenbroek, R. J., Kremer, H. P. H., Spoorenberg, S. L. W., Reijneveld, S. A., & Wynia, K. (2017). Integrated Care for Older Adults Improves Perceived Quality of Care: Results of a Randomized Controlled Trial of Embrace. *J Gen Intern Med*, *32*(5), 516–523. https://doi.org/10.1007/s11606-016-3742-y

Valdivieso, B., Garcia-Sempere, A., Sanfelix-Gimeno, G., Faubel, R., Librero, J., Soriano, E., Peiro, S., & GeChronic, G. (2018). The effect of telehealth, telephone support or usual care on quality of life, mortality and healthcare utilization in elderly high-risk patients with multiple chronic conditions. A prospective study. *Med Clin (Barc)*, *151*(8), 308–314. https://doi.org/10.1016/j.medcli.2018.03.013

van der Weegen, S., Verwey, R., Spreeuwenberg, M., Tange, H., Van Der Weijden, T., & De Witte, L. (2015). It’s LiFe! Mobile and Web-Based Monitoring and Feedback Tool Embedded in Primary Care Increases Physical Activity: A Cluster Randomized Controlled Trial. *Journal of Medical Internet Research*, *17*(7). https://doi.org/10.2196/jmir.4579

van Dijk-de Vries, A., van Bokhoven, M. A., Winkens, B., Terluin, B., Knottnerus, J. A., van der Weijden, T., & van Eijk, J. T. M. (2015). Lessons learnt from a cluster-randomised trial evaluating the effectiveness of Self-Management Support (SMS) delivered by practice nurses in routine diabetes care. *BMJ Open*, *5*(6), e007014. https://doi.org/10.1136/bmjopen-2014-007014

van Lieshout, M. R. J., Bleijenberg, N., Schuurmans, M. J., & de Wit, N. J. (2018). The Effectiveness of a PRoactive Multicomponent Intervention Program on Disability in Independently Living Older People: A Randomized Controlled Trial. *The Journal of Nutrition, Health & Aging*, *22*(9), 1051–1059. https://doi.org/10.1007/s12603-018-1101-x

Wolf, A., Fors, A., Ulin, K., Thorn, J., Swedberg, K., & Ekman, I. (2016). An eHealth Diary and Symptom-Tracking Tool Combined With Person-Centered Care for Improving Self-Efficacy After a Diagnosis of Acute Coronary Syndrome: A Substudy of a Randomized Controlled Trial. *J Med Internet Res*, *18*(2), e40. https://doi.org/10.2196/jmir.4890

Wong, A. K. C., Wong, F. K. Y., & Chang, K. (2019). Effectiveness of a community-based self-care promoting program for community-dwelling older adults: A randomized controlled trial. *Age Ageing*, *48*(6), 852–858. https://doi.org/10.1093/ageing/afz095

Wong, K. Y. F., Wang, S. L., Ng, S. S. M., Lee, P. H., Wong, A. K. C., Li, H., Wang, W., Wu, L., Zhang, Y., & Shi, Y. (2022). Effects of a transitional home-based care program for stroke survivors in Harbin, China: A randomized controlled trial. *Age and Ageing*, *51*(2), afac027. https://doi.org/10.1093/ageing/afac027

Wong, K. Y. F., & Yeung, S. M. (2015). Effects of a 4-week transitional care programme for discharged stroke survivors in Hong Kong: A randomised controlled trial. *Health & Social Care in the Community*, *23*(6), 619–631. https://doi.org/10.1111/hsc.12177

Woo, J., Yu, R., Leung, G., Chiu, C., Hui, A., & Ho, F. (2021). An Integrated Model of Community Care for Older Adults: Design, Feasibility and Evaluation of Impact and Sustainability. *Aging Medicine and Healthcare*, *12*(3), 105–113. https://doi.org/10.33879/AMH.123.2021.07067

Zakrisson, A. B., Hiyoshi, A., & Theander, K. (2016). A three-year follow-up of a nurse-led multidisciplinary pulmonary rehabilitation programme in primary health care: A quasi-experimental study. *J Clin Nurs*, *25*(7–8), 962–971. https://doi.org/10.1111/jocn.13132

Zhang, L., Zhang, L., Wang, J., Ding, F., & Zhang, S. (2017). Community health service center-based cardiac rehabilitation in patients with coronary heart disease: A prospective study. *BMC Health Serv Res*, *17*(1), 128. https://doi.org/10.1186/s12913-017-2036-3

Zhang, P., Xing, F. M., Li, C. Z., Wang, F. L., & Zhang, X. L. (2018). Effects of a nurse-led transitional care programme on readmission, self-efficacy to implement health-promoting behaviours, functional status and life quality among Chinese patients with coronary artery disease: A randomised controlled trial. *J Clin Nurs*, *27*(5–6), 969–979. https://doi.org/10.1111/jocn.14064
