# Supplementary material for: The Role of Nurses in Rehabilitation in Primary Health Care for Ageing Populations: A Secondary Analysis from a Scoping Review
Source: SAGE Open Nurs. 2024 Sep 23;10:23779608241271677. doi: 10.1177/23779608241271677 (PMC11425760; doi:10.1177/23779608241271677)
Supplement: sj-docx-10-son-10.1177_23779608241271677 - Supplemental material for The Role of Nurses in Rehabilitation in Primary Health Care for Ageing Populations: A Secondary Analysis from a Scoping Review [file sj-docx-10-son-10.1177_23779608241271677.docx]

***Appendix B Supplementary file 2***

| Key words Training about… | Authors  (year) | Trainings described in the studies (copy / pasted from the studies) |
| --- | --- | --- |
| Assessments | Markle‐Reid et al. (2018) | Nurses were trained to learn the needs assessment. Training not further specified. |
| Care plans | Blom,et al.  (2016) | Training for both GPs and practice nurses together: Session 1 with theory on care plans using a functional integrative approach. Practicing care plans for own patient and discussing care plans in the group. Planning for the 10 care plans in the study. Session 2 for discussing care plans for own patients. Plan organizing the intervention in own practice, i.e. allocate responsibilities, care plan making and registering, organizing multidisciplinary meetings, evaluating care plans, organizing a list of community resources for older people. Session 3 for developing an overview of local resources for the own region together with practice nurses. Discussion on fall interventions with occupational therapists. |
| Case management and shared decision making | Uittenbroek, et al. (2017) | Social workers and district nurses received specific training in areas such as case management and shared decision making during an 8-day initial training program. |
| Clinical experience | Coskun and Duygulu (2022) | Coordinator Nurse had clinical experience as a nurse for 5 years in the cardiovascular surgery clinic. |
|  | He et al.  (2018) | Nurses had stroke experience. Training not further specified. |
|  | Ko et al.  (2019) | Nurses had experience in caring for hospitalized older adults for several years. Training not further specified. |
|  | Ekelund and Eklund (2015) | Nurses had geriatric competence at the emergency department. Training not further specified. |
|  | Rasmussen et al. (2016) | Nurses had experience in stroke treatment. |
| Clinical experience and discharge planning | Zhang et al.  (2018) | Nurses had at least 10 years of medical nursing and discharge planning experiences. Some nurses undertook training in understanding and following the guidelines of this program. A postgraduate nursing student assisted the nurses in this project. |
| Clinical experience and fall-related physical activity programs | Tarazona-Santabalbina et al. (2016) | All the group members had at least 10 years of experience. They had previously participated in 2 physical activity-related falls prevention programs. |
| Clinical experience and training | Zhang et al.  (2017) | Cardiac nurses had expertise in cardiac rehabilitation, community nurses were trained and designated to promote the group activities and responsible for providing consultation for them during the whole period of the study. |
| Clinical experience, training on Omaha system, transitional care, assessment environment, holistic care intervention protocol | Wong et al.  (2015) | Nurses who had relevant experience in stroke care and in the community setting received a 3-day training workshop on the Omaha system, transitional care, Assessment of environment, telephone follow-up and the holistic care intervention protocols. The workshop included theoretical input and training cases. |
| Clinical specializations | Clevenger et al. (2018) | Advanced practice registered nurses (APRNs) had specializations in neurology, geriatrics, and palliative care. Training not further specified. |
| Depression and behavioral activation | Bekelman et al. (2015) | Before enrollment began, the nurse coordinator from each site participated in a 2-day training on depression and behavioral activation by the lead study psychiatrist (M.D.S.) and 4 weekly follow-up calls with the lead study psychiatrist. |
| SMS skills | Van Dijk-de Vries et al. (2015) | During three 8 h training sessions, Practice nurses (PNs) in the intervention arm were trained to integrate the detection and follow-up phase of SMS into their daily practice. The training sessions were followed up by booster sessions to maintain and improve PNs’ skills in SMS. PNs attended these booster sessions three or four times during the year of follow-up. |
| Education and training on COPDnet model | Koolen et al.  (2020) | Education and training sessions were offered depending on the specific needs indicated by the health care professionals. The topics of the education and training may have been varying, but they were always related to the COPDnet model. |
| General training | Godtfredsen et al. (2018) | Community nurses received general training, which was not further specified. |
| Geriarics and community care | Buurman et al.  (2016) | The nurses received training in geriatrics and community care. |
| Geriatrics and gerontology training | Bleijenberg et al. (2017) | U-PROFIT: Registered Nurses were extensively trained in a 6-week post-Bachelor level geriatrics / gerontology training of 48 hours. The nurses had monthly training meetings and feedback. HCP: Advanced practice nurses were specially trainned in primary care, with 1 week training session of 42 hours/ongoing detailed documentation combined with regular clinical briefing sessions during the intervention period. |
| Goal setting, motivational interviewing, use of PAM and the intervention, patient vignettes, role play with volunteers | Kidd et al.  (2015) | The training incorporated an educational session on the components of the intervention (e.g. goal setting, motivational interviewing, use of the PAM), reading materials and a copy of the study protocol, which outlined relevant study details including purpose and nature of the intervention, sample inclusion/exclusion criteria and intervention procedures, a list of motivational interviewing prompts that could be used during the intervention, face to face training in using the intervention, including patient vignettes, and role play to test out the intervention with volunteer stroke survivors who were specifically invited to take part in the training session through one of the clinical leads (not involved in the development or evaluation of the intervention itself). |
| Not specified | Ruikes et al.  (2016) | Not applicable. |
|  | Cameron-Tucker et al. (2016) | Not applicable. |
|  | Bleijenberg et al. (2016) | Nurses were extensively trained during a 6-week training program (48 hours total). Content of the training not further specified. |
| Personal instruction on "Five A's model", counseling techniques, and instructions for consultations | Van der Weegen et al. (2015) | For mastering the execution of the intervention, practice nurses in groups 1 and 2 received an online web lecture and consecutively a personal instruction session at their workplace. In addition, they received on paper, an explanation of the Five A’s model, the associated counseling techniques, and detailed instruction charts for each consultation. Nurses in group 1 were able to try out the tool before the start of the consultations. |
| Rehabilitation nursing course, competence test and intervention in a practice case | Kam Yuet Wong et al. (2022) | Since the intervention nurse had the specific role of stroke case manager, she was sponsored to attend a national post-basic stroke rehabilitation nursing course (72 hours), co-organized by a Guangdong hospital and a Hong Kong Foundation. The nurse passed a competence test and intervened satisfactorily in a practice case before the commencement of the study intervention. |
| Self-management and knowledge on arthritis | Leung et al.  (2016) | The lay leaders were trained in small group within the "Train the trainers program”. They were taught leadership and basic principles of self-management and provided with knowledge of chronic inflammatory arthritis by a designated rheumatologist. |
| Teaching experience | Sok et al.  (2021) | Nurses had more than 3 years of teaching experience at a university. Training not further specified. |
| Work in general practice, effective liaison and delivery of integrated physical and mental health care | Coventry et al.  (2015) | Practice nurses attended a half day workshop, where they met the psychological wellbeing practitioners tasked to work in their general practice and were introduced to the COINCIDE care model with an emphasis on effective liaison and delivering integrated physical and mental health care. |
