# Supplementary material for: The Role of Nurses in Rehabilitation in Primary Health Care for Ageing Populations: A Secondary Analysis from a Scoping Review
Source: SAGE Open Nurs. 2024 Sep 23;10:23779608241271677. doi: 10.1177/23779608241271677 (PMC11425760; doi:10.1177/23779608241271677)
Supplement: sj-docx-11-son-10.1177_23779608241271677 - Supplemental material for The Role of Nurses in Rehabilitation in Primary Health Care for Ageing Populations: A Secondary Analysis from a Scoping Review [file sj-docx-11-son-10.1177_23779608241271677.docx]

***Appendix B Supplementary file 3***

| **Domains and competencies** | **Studies’ rehabilitation interventions provided by nurses** | **N** | **%** | **N(%)** |
| --- | --- | --- | --- | --- |
| **Domain 1: Nurse-led interventions** | | **250** | **70** | **250(70)** |
| **1.1 Use supportive technology for improved QOL** | Provision and training in the use of assistive products | 2 | 1 | 2(1) |
| **1.2 Implement interventions based on best evidence** | Assessments (functioning, CGA, emotional functions, fall risk, frailty, environment, health status, medication, cognitive functions, family and caregivers' needs, knowledge and skills) | 74 | 21 | 74(21) |
|  | Restorative and compensatory interventions (all interventions) | 72 | 20 | 72(20) |
| **1.3 Provide patient & family education** | Education and counselling (all interventions) | 51 | 14 | 51(14) |
| **1.4 Understanding worldview of culturally different individuals** | Not applicable | NA | NA | NA |
| **1.5 Deliver patient & family-centered care** | Assessments of person-centered goals | 32 | 9 | 32(9) |
|  | Social care and support (all interventions) | 19 | 5 | 19(5) |
| **Domain 2: Promotion of health & successful living** | | **341** | **96** | **341(96)** |
| **2.1 Promote health & prevent disability** | Assessments (all interventions) | 106 | 30 | 106(30) |
|  | Restorative and compensatory interventions (all interventions) | 72 | 20 | 72(20) |
|  | Social care and support (all interventions) | 19 | 5 | 19(5) |
| **2.2 Foster self-management** | Education and skills training for self-care and self-management | 26 | 7 | 26(7) |
|  | Education and skills training for caregivers | 13 | 4 | 13(4) |
| **2.3 Promote and facilitate safe and effective care transitions** | Coordination and management of the rehabilitation process (all interventions) | 105 | 30 | 105(30) |
| **Domain 3: Leadership** | | **NA** | **NA** | **NA** |
| **3.1 Promote accountability for care** | Not applicable | NA | NA | NA |
| **3.2 Disseminate rehabilitation nursing knowledge** | Not applicable | NA | NA | NA |
| **3.3. Impact of health policy for persons with disability and / or chronic illness** | Not applicable | NA | NA | NA |
| **3.4 Empower patients to self-advocate** | Not applicable | NA | NA | NA |
| **Domain 4: Intra / interprofessional team** | | **NA** | **NA** | **NA** |
| **4.1 Develop intra / interprofessional relationships** | Not applicable | NA | NA | NA |
| **4.2 Implement an intra / interprofessional holistic POC** | Not applicable | NA | NA | NA |
| **4.3 Foster effective intra / interprofessional collaboration** | Not applicable | NA | NA | NA |
